# Electron transfer in biological systems

**DOI:** 10.1007/s00775-024-02076-8

**Published:** 2024-10-18

**Authors:** Helder M. Marques

**Affiliations:** Molecular Sciences Institute, School of Chemistry, University of the Witwatersrand, Johannesburg, 2050 South Africa

**Keywords:** Bioinorganic chemistry, Electron transfer, Cellular respiration, Photosynthesis, Defence against reactive oxygen species, Electron transport chain, Extracellular electron transfer

## Abstract

Examples of how metalloproteins feature in electron transfer processes in biological systems are reviewed. Attention is focused on the electron transport chains of cellular respiration and photosynthesis, and on metalloproteins that directly couple electron transfer to a chemical reaction. Brief mention is also made of extracellular electron transport. While covering highlights of the recent and the current literature, this review is aimed primarily at introducing the senior undergraduate and the novice postgraduate student to this important aspect of bioinorganic chemistry.

**Graphical abstract:**

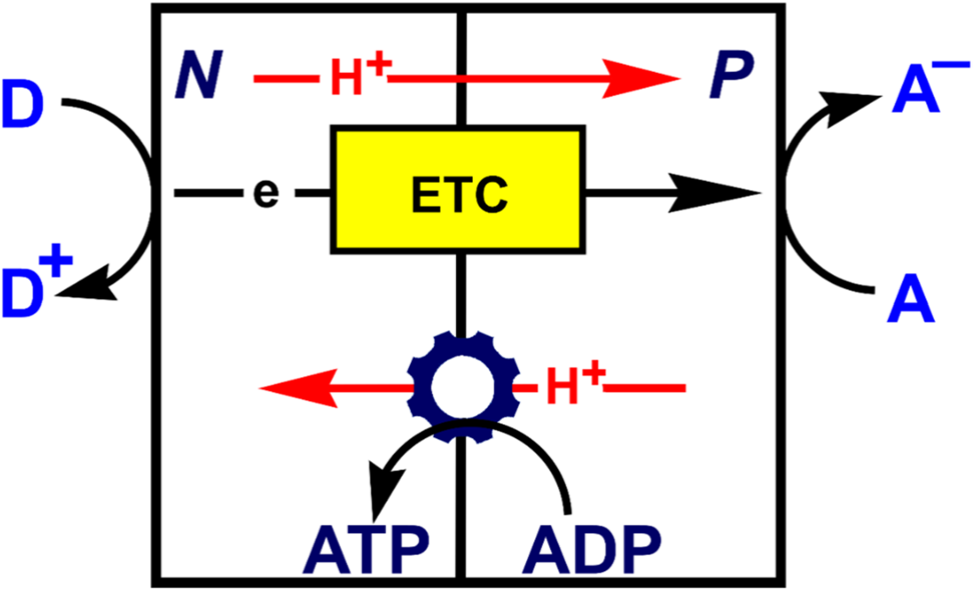

## Introduction

**Fig. 1:**
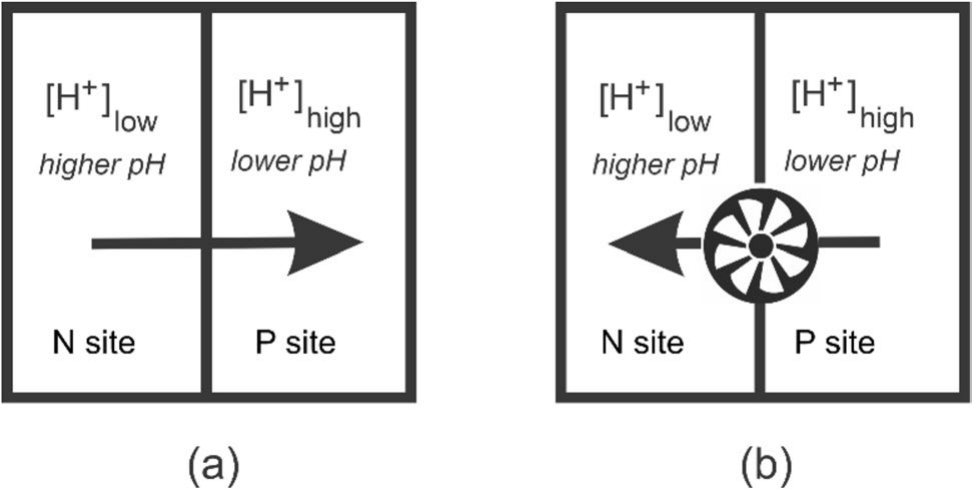
**a** Energy released from the reduction of an electron acceptor by an electron donor is used to pump protons across a membrane against a proton gradient, from the negative (N) side of the membrane to the positive (P) side. **b** Generated proton-motive force is used by molecular machinery to drive the non-spontaneous synthesis of a molecule such as ATP

Electron transport is central to life. In cellular respiration the thermodynamically spontaneous reduction of a terminal electron acceptor (such as O_2_) by an electron donor (such as NADH) drives the non-spontaneous pumping of protons across a membrane against a concentration gradient, setting up what is referred to as a proton-motive force [1]. This chemiosmotic coupling is used by molecular machinery for the synthesis of ATP, the energy currency of a cell (Fig. 1). Carrying electrons from the electron donor to the electron acceptor requires an electron transport chain (ETC).

The transfer of electrons is of fundamental importance in many other processes as well, including photosynthesis, metabolic regulation and redox reactions. Extensive studies of photosynthesis and respiration in prokaryotic and eukaryotic systems, processes that provide energy for living organisms, have provided us with much insight into these processes [2]. Less is known about extracellular electron transport processes but significant progress has been made relatively recently [3].

Electron transport relies upon the transfer of an electron(s) from one species to another down a chain driven by a series of donors and acceptors with successively increasing midpoint reduction potentials, *E*_1/2_, which provide the thermodynamic driving force for electron transfer. The variable oxidation state of metal ions such as Fe and Cu are invaluable in, for example, photosynthesis and cellular respiration. These electron transfer processes occur through an outer sphere mechanism, where the electron transfer occurs without the electron donor and electron acceptor being bridged by a common ligand. Factors that control the rate of outer sphere electron transfer (see Sect. "Maximising the rate of outer sphere electron transfer") are variables that nature can use to tune these chains.

In many reactions catalysed by metal ions, the substrate binds to the metal ion, or cluster of metal ions, and electron transfer occurs through an inner sphere mechanism, from the metal(s) to the substrate. There are many examples of this in biology, and several will be looked at in this article.

Cellular respiration begins with glycolysis which takes place in the cytoplasm of a cell. In the process, glucose is converted to pyruvate, producing some ATP (Eq. 1): 1

Pyruvate is then converted into acetyl coenzyme A (Eq. 2); this is transported into the mitochondria of cells (other processes apply to cells without mitochondria such as prokaryotes and erythrocytes, and to cells that contain mitochondria but are under anaerobic conditions) and enters the tricarboxylic acid cycle, a simplified view of which is shown in Fig. 2.2

**Fig. 2:**
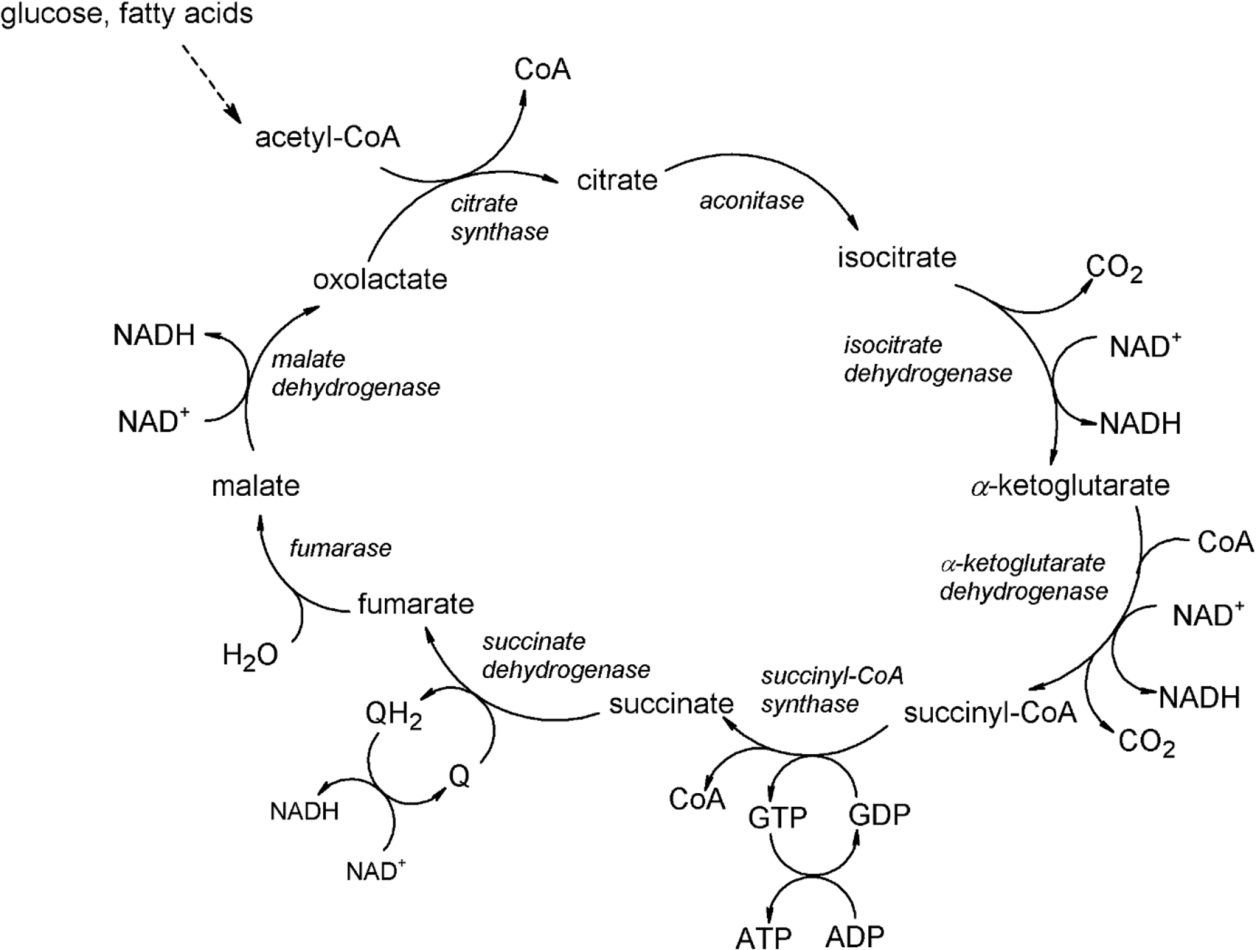
Krebs (citric acid) or tricarboxylic acid cycle. (Q is ubiquinone, or coenzyme Q.)

Electrons are then carried by NADH and ubiquinol (QH_2_) through an electron transport chain to cytochrome *c* oxidase (Complex IV) where O_2_ is reduced to H_2_O. The overall effect is, therefore, the oxidation of glucose (or fatty acids, or other metabolites) by O_2_. The energy produced is used to pump protons across the inner mitochondrial membrane, setting up a proton-motive force that is used by ATP synthase (Complex V) to drive the synthesis of ATP.

**Fig. 3:**
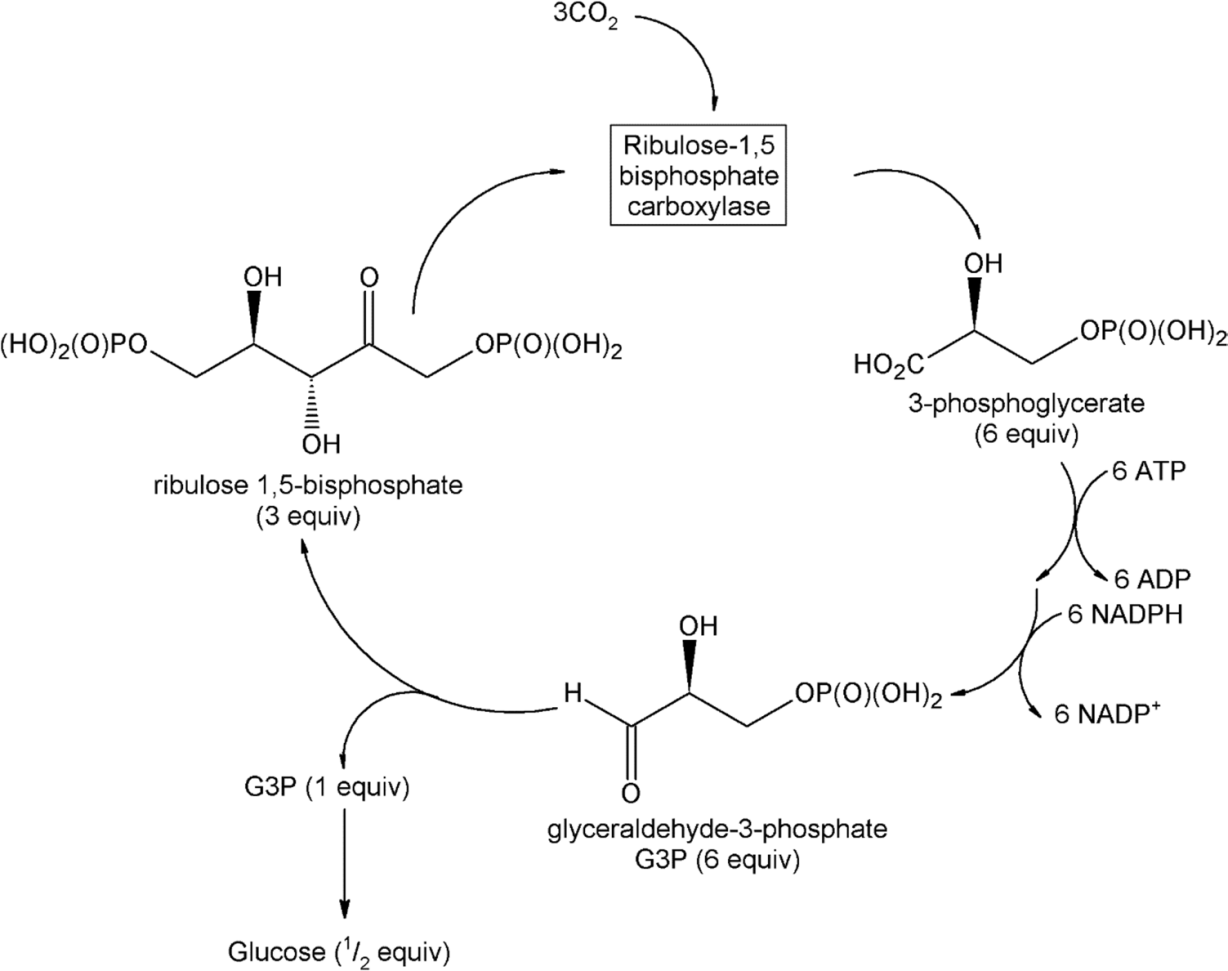
Calvin cycle

Photosynthesis occurs in green plants, algae and some bacteria. In plants it occurs in the thylakoid membrane of chloroplasts, a membrane-bound organelle (plastid). Light energy is captured by light-harvesting pigments; the energy so gathered is used to drive electrons through an electron transport chain. A proton-motive force is used to synthesise ATP from ADP, and to produce NADPH, an important energy carrier, from NADP^+^. ATP and NADPH are used in the Calvin cycle (Fig. 3) to covert CO_2_ into organic compounds such as glucose, eventually leading to the synthesis of carbohydrates.

Among the techniques used to investigate electron transfer process are in situ and *operando* electrochemical methods, often coupled with spectroscopic, spectrometric and microscopic techniques [4] and dynamic electrochemical methods [5] such as protein monolayer voltammetry [6, 7]. An understanding of the behaviour of redox-active enzymes, organelles or living microbes at an electrode surface is important for the development of bioelectrocatalysis for biofuel cells, biosensors and a variety of bioelectrochemical devices [8–12].

Electron transport in biological systems is an immense field. Therefore, this article is necessarily limited in its scope and is aimed at introducing and providing an overview of electron transport in biological systems to the senior undergraduate—or novice postgraduate—student interested in bioinorganic chemistry.

## Metalloproteins in *electron* transfer chains

The long-range transfer of electrons by systems such as the cytochromes, the blue copper proteins and the ferredoxins, metalloproteins that are responsible for accepting an electron from a reductant and passing it on to an oxidant, plays an important role in biology [13]. Factors that affect the efficiency of these metalloproteins in fulfilling their function include the ligands that coordinate the metal ion; the coordination geometry of the metal; the location of the metal binding site within the protein which controls the electron transfer distance between the electron donor and the electron acceptor; and the redox potential—itself controlled by many factors including the identity of the metal, the structure and geometry of its coordination sphere, the structure of the protein itself, and the extent of the exposure of the metal complex to solvent [13]. How the redox potential, *E*_1/2_, pH 7 (vs SHE) of an iron porphyrin depends on the electron donor ability (softness) of the proximal ligand is illustrated by catalase (Tyr, *E*_1/2_ < –500 mV), P450cam (Cys, – 170 mV) and myoglobin (His, 50 mV) [14] (see Sect. "Examples of metalloproteins that directly couple electron transfer to chemical reactions").

### Getting hold of *iron*

Iron plays a key role in biology and the biological redox potential of iron spans a very wide range. For example, *E*_1/2_ < –500 mV for the human iron transport protein transferrin, used for carrying iron in the body. Animals acquire iron from their food. Nature’s ambient “electrochemical window” is limited to potentials between the oxidation of H_2_O with evolution of O_2_ and the reduction of H^+^ to produce H_2_. Under standard conditions at pH 7, this is + 817 mV and – 413 mV, respectively. However, before iron could be fully exploited for its biological role, the solubilising of Fe^3+^ (*K*_sp_ ≈ 10^–39^ at pH 7 [15]) was a problem that life had to solve after the “Great Oxidation Event” c. 2.7 billion years ago which changed Earth’s atmosphere from mildly reducing to oxidising due to the action of blue–green algae which displaced atmospheric methane with oxygen [16–19]. Chelators were the answer found.

**Fig. 4:**
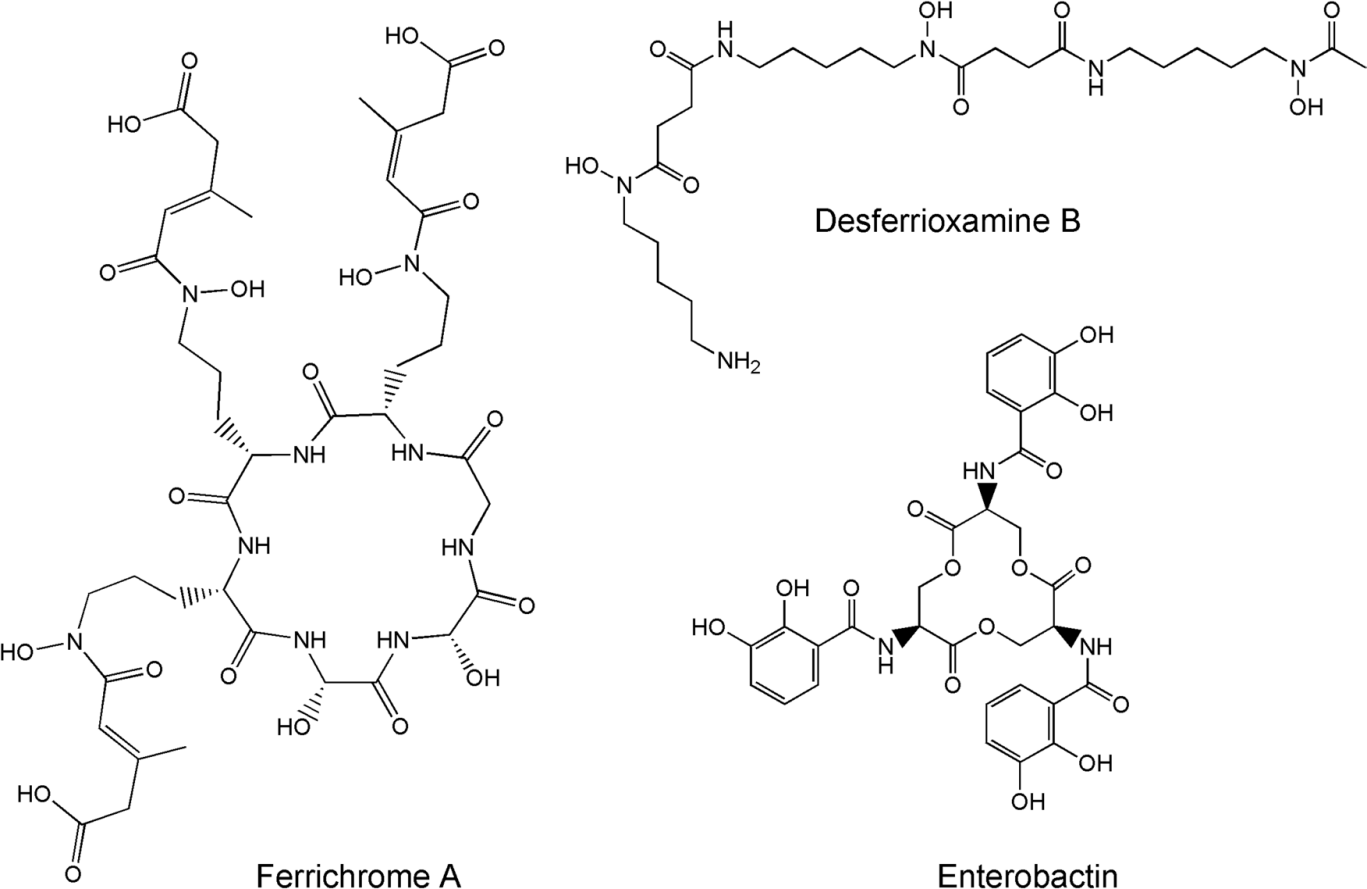
Structure of some siderophores

Ferrichromes and other siderophores are ligands used by prokaryotes for binding Fe^3+^, thus solubilising it for metabolic incorporation [20–22]. For example, ferriochrome A and desferrioxamine B are found among fungi, yeasts and moulds; bacteria tend to use chelates based on catechols, for example, enterobactin from *E. coli* (Fig. 4). As expected, these ligands use hard donors to complex hard Fe^3+^.

Once captured, iron must be released. Cyclic voltammetry of Fe^3+^ complexes of ferrichrome A and ferrioxamine B show reversible one-electron waves with pH-independent formal potentials of – 446 and – 454 mV, respectively, well within the range of physiological reductants [23]. By contrast, *E*_1/2_ for the Fe^3+^ complexes of enterobactin ≈ – 750 mV; release of Fe^3+^ from this chelator cannot occur by reduction to Fe^2+^ under physiological conditions, and requires the hydrolysis of the chelator to effect release of the metal ion.

### Reactive oxygen (and other) species

An oxidizing atmosphere presented life with another problem. The use of oxygen to oxidise the fuels that drive the production of energy in living systems was an important step, but the formation of reactive oxygen species (ROS) such as singlet oxygen ^1^O_2_, superoxide O_2_^•–^, hydrogen peroxide H_2_O_2_ and (especially) the hydroxyl radical OH^•^ was a major issue that had to be overcome to enable the evolution of aerobic multicellular organisms [24]. ROS cause damage to cellular constituents, including proteins, lipids and DNA [25].

Of particular importance is preventing the formation of OH^•^, which will react rapidly and indiscriminately with virtually all biomolecules. The storage of Fe^3+^ as ferrihydrate by the blood protein ferritin minimises the occurrence of “free iron” and hence minimises the production of ROS. The production of ROS is often (mistakenly) attributed to the Haber–Weiss cycle, catalysed by Fe^3+^. (It should be emphasised that “free iron” refers to an Fe^3+^ complex such as ferric citrate since at physiological pH Fe^3+^ has negligible solubility [26]. In the equations that follow it is understood that Fe^3+^ and Fe^2+^ are present as a complex):

3 $$\left[ {{\text{Fe}}^{{{3} + }} } \right] \, + {\text{ O}}_{{2}}^{{ \bullet {-}}} \to \, \left[ {{\text{Fe}}^{{{2} + }} } \right] \, + {\text{ O}}_{{2}}$$

4 $$\left[ {{\text{Fe}}^{{{2} + }} } \right]^{{\phantom{a}}} + {\text{ H}}_{{2}} {\text{O}}_{{2}} \to \, \left[ {{\text{Fe}}^{{{3} + }} } \right] \, + {\text{ OH}}^{ \bullet } + {\text{ OH}}^{-} \left( {\text{Fenton reaction}} \right)$$

5 $${\text{O}}_{{2}}^{{ \bullet {-}}} + {\text{ H}}_{{2}} {\text{O}}_{{2}} \rightleftharpoons {\text{O}}_{{2}} + {\text{ OH}}^{ \bullet } + {\text{ OH}}^{-} \left( {{\text{net reaction}},{\text{ the Haber}} - {\text{Weiss reaction}}} \right)$$

As has been pointed out [26, 27], O_2_^•–^ reacts faster with itself than with H_2_O_2_ [28]. The Fenton reaction, however, *is* important in a biological system because it initiates a chain reaction (Eqs. 6, 7), which is then terminated (Eq. 8):

6 $${\text{OH}}^{ \bullet } + {\text{ H}}_{{2}} {\text{O}}_{{2}} \to {\text{ O}}_{{2}}^{{ \bullet {-}}} + {\text{ H}}_{{2}} {\text{O }} + {\text{ H}}^{ + }$$

7 $${\text{O}}_{{2}}^{{ \bullet {-}}} + {\text{ H}}_{{2}} {\text{O}}_{{2}} + {\text{ H}}^{ + } \to {\text{ O}}_{{2}} + {\text{ H}}_{{2}} {\text{O}}_{{\phantom{a}}} + {\text{ OH}}^{ \bullet }$$

8 $$\left[ {{\text{Fe}}^{{{2} + }} } \right] \, + {\text{ OH}}^{ \bullet } + {\text{ H}}^{ + }_{{\phantom{a}}} \to \, \left[ {{\text{Fe}}^{{{3} + }} } \right] \, + {\text{ H}}_{{2}} {\text{O}}$$

In addition, the Fenton reaction may produce higher oxidation states of iron such as FeO^2+^ (see [26] and references therein.)

In the presence of bicarbonate in neutral solutions, the Fenton reaction also produces carbonate radical anions, CO_3_^•–^ [29] (Eqs. 9–11), which, it has been suggested, may be even more damaging than OH^•^ to DNA [30]. Coordinated CO_3_^2–^ lowers the redox potential of Fe^3+^ [31], permitting formation of Fe^4+^, key for the splitting of the O–O bond of coordinated HO_2_^–^. (We shall see later the importance of high oxidation states of metal ions for cleaving the bond of dioxygen.)

9 $$\left[ {{\text{Fe}}^{{{\text{II}}}} \left( {{\text{H}}_{{2}} {\text{O}}} \right)_{{6}} } \right]^{{{2} + }} + {\text{ HCO}}_{{3}}^{-} \rightleftharpoons \left[ {{\text{Fe}}^{{{\text{II}}}} \left( {{\text{CO}}_{{3}} } \right)\left( {{\text{H}}_{{2}} {\text{O}}} \right)_{{3}} } \right] \, + {\text{ H}}_{{3}} {\text{O}}^{ + } + {\text{ 2H}}_{{2}} {\text{O}}$$

10 $$\left[ {{\text{Fe}}^{{{\text{II}}}} \left( {{\text{CO}}_{{3}} } \right)\left( {{\text{H}}_{{2}} {\text{O}}} \right)_{{3}} } \right]_{{\phantom{a}}} + {\text{ H}}_{{2}} {\text{O}}_{{2}} \rightleftharpoons \left[ {{\text{Fe}}^{{{\text{II}}}} \left( {{\text{CO}}_{{3}} } \right)\left( {{\text{HO}}_{{2}} } \right)\left( {{\text{H}}_{{2}} {\text{O}}} \right)_{{2}} } \right]^{-} + {\text{ H}}_{{3}} {\text{O}}^{ + }$$

11 $$\left[ {{\text{Fe}}^{{{\text{II}}}} \left( {{\text{CO}}_{{3}} } \right)\left( {{\text{HO}}_{{2}} } \right)\left( {{\text{H}}_{{2}} {\text{O}}} \right)_{{2}} } \right]^{-} \to \, \left[ {{\text{Fe}}^{{{\text{IV}}}} \left( {{\text{CO}}_{{3}} } \right)\left( {{\text{OH}}} \right)_{{3}} \left( {{\text{H}}_{{2}} {\text{O}}} \right)} \right]^{-} \to \, [{\text{Fe}}^{{{\text{III}}}} \left( {{\text{OH}}} \right)_{{3}} \left( {{\text{H}}_{{2}} {\text{O}}} \right] \, + {\text{ CO}}_{{3}}^{{ \bullet {-}}}$$

Carbonate radical anions are also produced in the oxidation of [Fe^2+^]_aq_ by O_2_ in the presence of bicarbonate [32]. Moreover, bicarbonate reacts with OH^•^ to form CO_3_^•–^ [32] (Eq. 12). CO_3_^•–^ causes oxidative damage to biomolecules such as DNA [33, 34]:

12 $${\text{OH}}^{ \bullet } + {\text{ HCO}}_{{3}}^{-} \to {\text{ CO}}_{{3}}^{{ \bullet {-}}} + {\text{ H}}_{{2}} {\text{O}}$$

Many enzymes, including catalases, peroxidases, superoxide dismutases, superoxide reductases, peroxiredoxins and glutathione peroxidases, have evolved to minimise the inevitable formation of ROS (and OH^•^ in particular) in an oxidising environment by scavenging H_2_O_2_ and O_2_^•–^ [26, 35]. Elevated levels of hydrogen peroxide and superoxide in the mitochondrial matrix have been implicated in many diseases [36]. The generating of ROS and their control is looked at in more detail in Sect. "The Catalases, Peroxidases, Superoxide Dismutases and Superoxide Reductases"

**Fig. 5:**
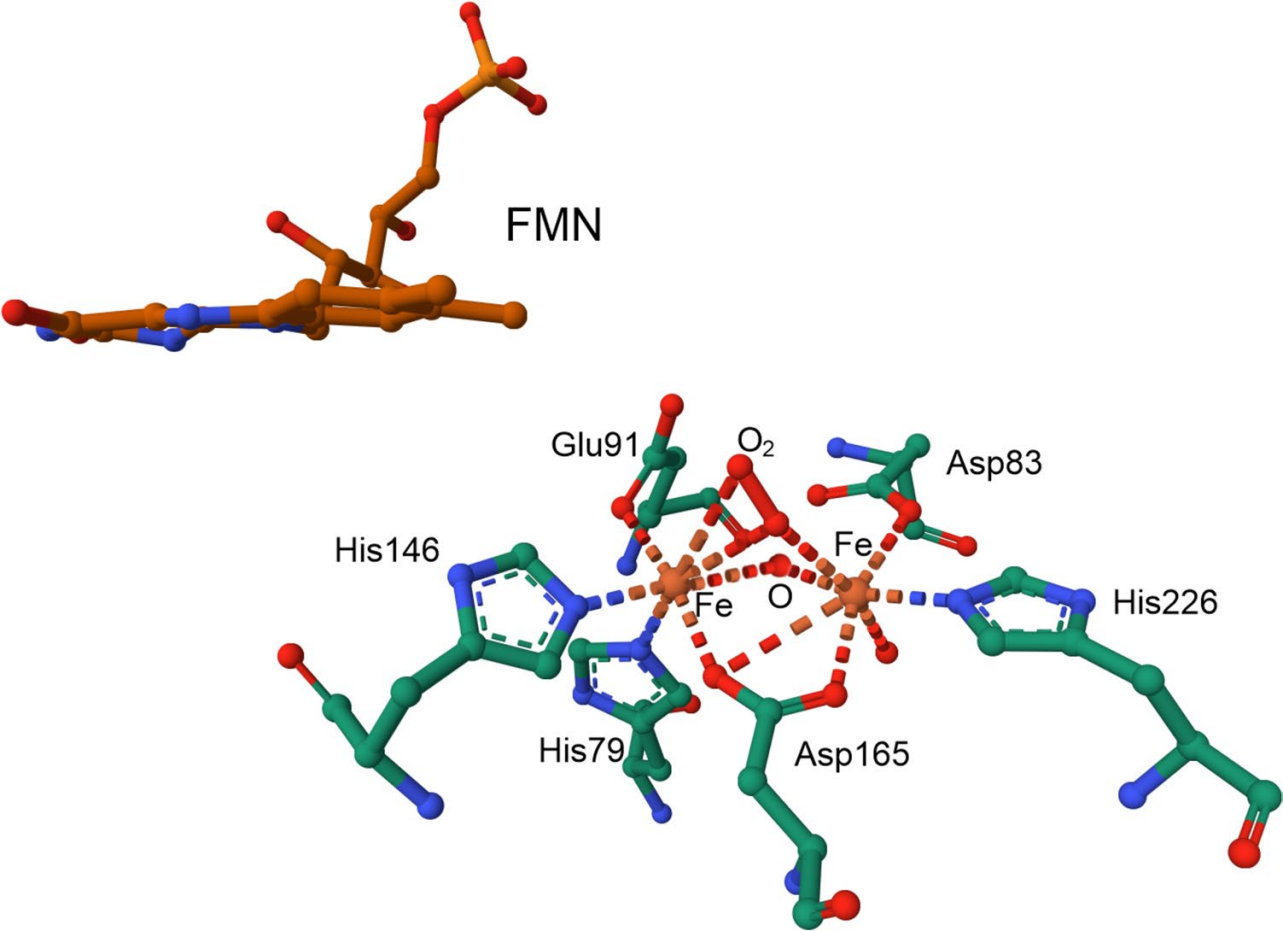
Diiron O_2_ binding site and neighbouring FMN in the rubredoxin:oxygen oxidoreductase from *Desulfovibrio gigas* (PDB code 1E5D [39])

Some organisms (archaea, bacteria, some protozoan pathogens) have flavodiiron proteins (FDPs) for protection against NO and, in the case of anaerobes, O_2_ itself. The proteins have a non-haem diiron centre which is the site of NO and/or O_2_ reduction [37–39]. A well-characterised system is that from *Desulfovibrio gigas* [39]. Its electron transport chain consists of three proteins: NADH:rubredoxin oxidoreductase, rubredoxin, and rubredoxin:oxygen oxidoreductase (ROO). In brief, the electron transport chain is NADH → rubredoxin → ROO → O_2_. ROO contains a flavin mononucleotide (FMN) which is reduced by rubredoxin. This in turn transfers electrons to the diiron site where reduction of O_2_ to H_2_O occurs. The diiron-site with bound O_2_ and μ-O (or possibly OH^–^), and the nearby FMN, is shown in Fig. 5.

**Fig. 6:**
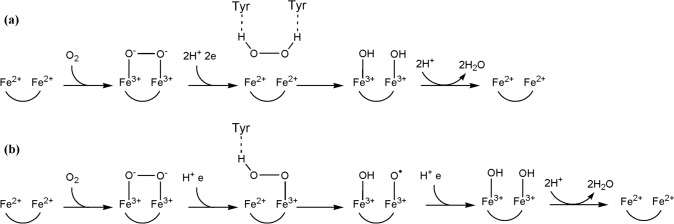
Two possible mechanisms suggested by DFT calculations for the formation of H_2_O from O_2_ at the diiron site of ROO [38] depending in whether **a** PCET from FMN is fast or **b** slow. Tyr residues near the active site play an important role in enzyme turnover

DFT calculations (B3LP-D3) suggest that the mechanism depends on the rate of proton-coupled electron transfer (PCET) from FMN [38] (Fig. 6).

In addition to ROS, reactive nitrogen species (RNS) and reactive sulfur species (RSS) are deleterious to living systems and need to be kept under control.

RNS are a group of highly reactive species derived from NO and O_2_^•–^ that damage proteins, nucleic acids and lipids. Elevated levels of RNS lead to cell injury and death by inducing nitrosative stress [40]. NO is produced from L-arginine and O_2_ by nitric oxide synthases (mammals have three isoforms of these enzymes) and performs important functions as a signalling molecule involved in, for example, the regulation of blood pressure and vasodilation [41]. The reaction of NO and O_2_^•–^ produces peroxynitrite, ONOO^–^, a highly reactive species implicated in oxidative stress [42]. As discussed later (see Sect. "The Catalases, Peroxidases, Superoxide Dismutases and Superoxide Reductases"), ONOO^–^ reacts with CO_2_ to produce CO_3_^•–^ [30, 43]. Control of O_2_^•–^ is, therefore, imperative. Other RNS include NO_2_, formed from the reaction of NO with O_2_ or O_3_, which can cause damage to the human respiratory tract [44] and N_2_O_3_, formed from the reaction of NO and NO_2_, which may play a role in hypoxic vasodilation and protein S-nitrosylation, and has been implicated in a broad spectrum of human diseases when dysregulated [45, 46].

Like NO (and CO), H_2_S is an important bioactive signalling agent produced by cystathionine-*β*-synthase, cystathionine-*γ*-lyase and 3-mercapto-sulfurtransferase in mammals [47]. It plays a neuroprotective function in Alzheimer’s disease and in traumatic brain injury [48]. Its reaction with disulfide bonds in proteins or low molecular weight thiols produces persulfides (RSSH) [49], and its oxidation or interaction with other sulfur-containing species, produces a variety of polysulfides (H_2_S_*x*_, *x* > 1) [50]. These are all reactive sulfur species (RSS). Other RSS include sulfenic acid (RSOH) formed from the oxidation of protein Cys residues [51], and sulfite generated during the metabolic processing of sulfur-containing compounds [52]. Like ROS and RNS, RSS can cause oxidative damage to proteins, nucleic acids and lipids, leading to cell dysfunction and death [53, 54].

### The mitochondrial *electron* transport chain

**Fig. 7:**
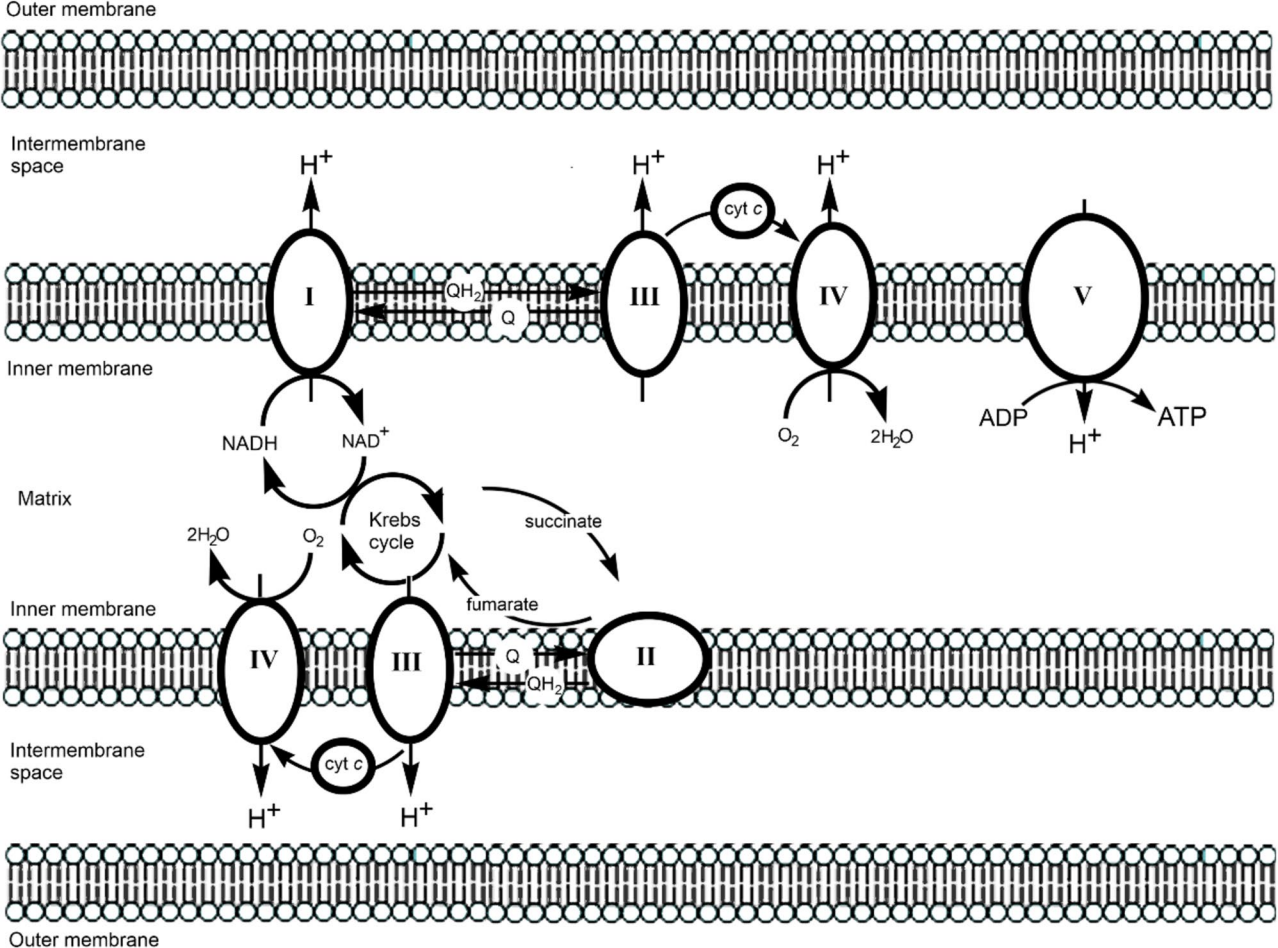
Mitochondrial electron transport chain. Adapted from [60]

**Fig. 8:**
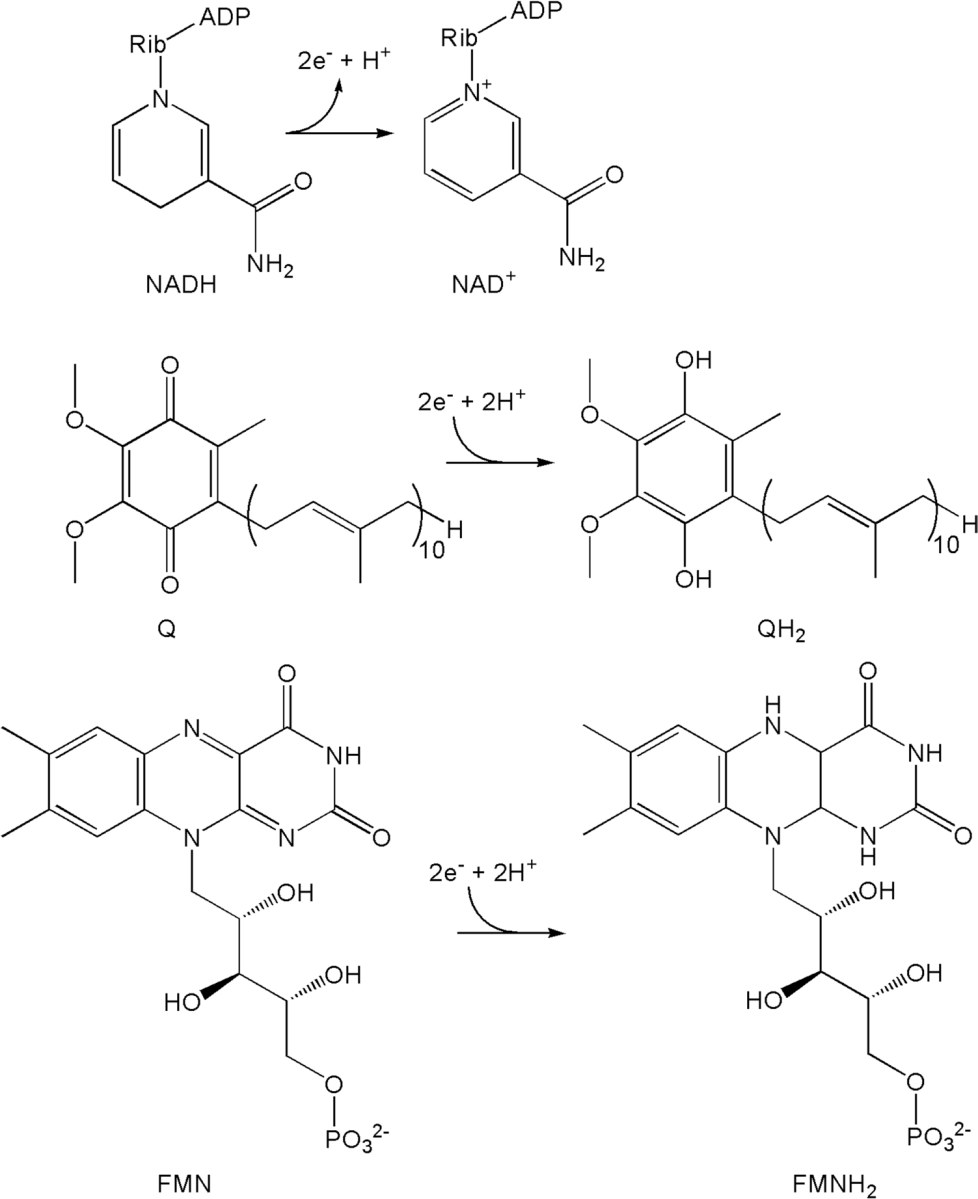
Redox reactions of Complex I of the respiratory chain of the mitochondria

The classic example of an electron transport chain is that found in the inner membrane of the mitochondria, the “power house” of the cell, speculated to be the consequence of an ancient endosymbiotic event [55]; a simplified view of the chain is given in Fig. 7. The overall reaction sequence is given in Eq. 13. (See Fig. 8 for the abbreviations used in what follows.)

13 $${\text{NADH }} \to {\text{ Complex I }} \to {\text{ QH}}_{{2}} \to {\text{ Complex III }} \to {\text{ cytochrome c }} \to {\text{ Complex IV }} \to {\text{ O}}_{{2}}$$

Complex I (NADH:ubiquinone oxidoreductase), which has been called the engine of the cell [56], catalyses the transfer of electrons from NADH, generated in the Krebs cycle (Fig. 2), to ubiquinone, Q [57] (Eq. 14):

14 $${\text{NADH }} + {\text{ H}}^{ + } + {\text{ Q }} + {\text{ 4H}}^{ + }_{in} \to {\text{ NAD}}^{ + } + {\text{ QH}}_{{2}} + {\text{ 4H}}^{ + }_{out}$$

The subscripts *in* and *out* refer to the matrix and the intermembrane space, respectively. Proton pumping is also carried out by Complex III (cytochrome *bc*_1_) and Complex IV (cytochrome *c* oxidase.) This chemiosmotic coupling [58, 59] sets up a transmembrane potential generating a proton-motive force that is used by Complex V (ATP synthase) in the synthesis of ATP. The reactants and products of Complex I are shown in Fig. 8.

The ETC is an elegant example of how suitable iron is for performing biological functions. It is a geologically abundant element (the fourth most abundant element in the earth’s crust [61]) and its concentration as Fe^2+^ in the oceans, under a mildly reducing atmosphere when life originated some 3.5 billion years ago, would have been high. It has thermodynamically stable, yet kinetically labile Fe^2+^ and Fe^3+^ oxidation states, can have different spin states (high, low, and intermediate) and readily accommodates 4, 5 or 6 ligands. Pseudo square–pyramidal complexes are ideally suited for accommodating a substrate as a sixth ligand (such as O_2_ in haemoglobin and myoglobin, and for the activation of oxygen and its derivatives in the oxidases, catalases and peroxidases).

An important concept in inorganic chemistry, introduced in 1962 by Gray [62–64] is the “oxo wall”. It is founded on the observation that metal–oxo tetragonal species with the metal in the + 4 and higher oxidation states are limited to elements of up to group 8 of the periodic table. There appears to be a wall, the oxo wall, between group 8 and group 9, the existence of which was rationalised using ligand field theory. Early first row d block metals readily form unreactive M=O species (such as [VO]^2+^) but after Fe, higher exudation states are difficult to attain. Thus, tetragonal Fe^4+^=O and Mn^5+^≡O species can be formed (and play an important role in biology) but not, for example, Co^4+^=O. (However, high valent states of cobalt (Co^4+^–oxo and Co^5+^–oxo) have been invoked to occur as intermediates in reactions catalysed by, for example, cobalt–oxo cubane clusters [65, 66]. A recent computational study—using both DFT and ab initio methods—shows that there is a mixing between Co^4+^=O and Co^3+^–O^•^ in a cobalt–oxo complex, with the former a breach of the oxo wall, and the extent of which can be tuned by varying the ligand architecture [67].) Metal–oxo complexes with the metal in a high oxidation state (as in Fe^4+^O and Mn^5+^O) cannot be stabilised by multiple bonding to an oxo ligand [63]. They are strongly electrophilic, making them exceptional catalytic intermediates; this is key to the reactions catalysed by enzymes with metal–oxo intermediates.

In Complex I of the mitochondrial ETC, iron is 4-coordinate in the iron–sulfur complexes, a coordination geometry dictated by the relatively large radius of sulfide. Six-coordinate iron porphyrins are ideally suited for electron transport, as exemplified by the two haems of cytochrome *b* and cytochrome *c*_1_ of Complex III (*E*_1/2_ = 120 mV, –  30 mV and 240 mV, respectively [68]). Both the high potential haem and the low potential haem have His as their two axial ligands [69]; but the first is located in a solvent-filled cavity, while the latter is protected from the solvent by cytochrome *c*_1_, an illustration of how the environment of a redox centre can be used to control its potential.

Haems (iron porphyrin complexes) feature prominently in ETCs, in oxygen transport and storage proteins, and in catalysts such at catalase, peroxidase and the cytochromes P450. They are more oxygen-tolerant than the iron–sulfur clusters and probably evolved later, in response to an oxidising atmosphere [70]. In *b-*type haems, the iron porphyrin is non-covalently housed within a hydrophobic crevice of the porphyrin and its axial ligand(s) are crucial for its correct positioning. In the *c-*type cytochromes, the haem group is covalently attached through thioether bonds between Cys residues and the vinyl groups of the porphyrin [71]. A C(X)_*n*_CH motif in the amino acid sequence is crucial for covalent attachment of the haem to the protein, providing covalent attachment sites as well as the proximal His ligand. Often *n* = 2, but, for example, a case where *n* = 15 [72] and one where *n* = 1 [73] are known. Some peptides carry multiple CXXCH motifs, allowing for the assembly of multi-haem proteins [74, 75]. (For more information on porphyrins, see the introduction to Sect. "Examples of metalloproteins that directly couple electron transfer to chemical reactions".)

#### Maximising the rate of outer sphere *electron* transfer

To maximise the rate of outer sphere electron transfer, changes in oxidation state should be accompanied by a minimal change in structure; this ensures a low reorganisation energy, one of the barriers to the process, as elucidated by the Marcus theory of electron transfer [76]. The rate of electron transfer, *k*_ET_, between an electron donor D and an electron acceptor A is given by the following equation:

15 $$k_{ET} \quad = \quad \frac{{2\left\langle {H_{DA}^{o} } \right\rangle^{2} e^{ - \beta r} }}{h}\left( {\frac{{\pi^{3} }}{4\lambda RT}} \right)^{1/2} e^{{ - \Delta G^{\ddag } /RT}}$$

where $\left\langle {H_{{{\text{DA}}}}^{{\text{o}}} } \right\rangle$ is the Hamiltonian that describes the coupling between the wavefunctions of D and A; *β* is a parameter that measures the sensitivity of the coupling to the edge-to-edge distance, *r*, between D and A; *λ* is the reorganisation energy, which is the energy required to change the equilibrium geometry of the reactants and products to a geometry where their potential energy surfaces intersect; and $\Delta G^{\ddag }$ is the Gibbs energy of activation, as defined in the following equation:

16 $$\Delta G^{\ddag } \; = \frac{1}{4}\lambda \left( {1 + \frac{{\Delta G^{{\text{o}}} }}{\lambda }} \right)^{2}$$

where Δ*G*^o^ is the change in Gibbs energy for an electron transfer from the ground state of D to the ground state of A and is, therefore, related to the standard reduction potentials of D and A by Δ*G*^o^ = –*nFE*^o^. The reorganisation energy is dependent on the medium surrounding the redox sites, and aqueous environments lead to larger values of *λ* [2]. The structure of the protein is also crucial, and *β* sheets (which are stiff) are more effective at coupling electron transfer processes than are *α* helices (which are much more flexible) [2].

Ensuring that* λ* is small, and hence, electron transfer is fast, is illustrated by the [2Fe–2S] centre of some ferredoxins, the [4Fe–4S] centre of others, and the Fe(Cys)_4_ centre of rubredoxins, iron–sulfur proteins that mediate one-electron transfer in many reactions in biology. Iron–sulfur clusters play other vital roles in biological systems; their biogenesis has been reviewed [77]. The similar energies of Fe(3d) and S(3p) orbitals allows for significant covalent character in the clusters, undoubtedly contributing to their easy change of oxidation state [78]. The change in oxidation state of each iron centre does not significantly affect its, in essence, tetrahedral structure [79] (see Sect. "A note on the iron-sulfur clusters"). The way fast electron transfer is carried out by copper-containing proteins is discussed in Sect. "The cytochrome b6f complex – and a note on the blue copper proteins". For a comprehensive review of mechanisms used for the reduction of *λ* in biological systems, see [80].

**Fig. 9:**
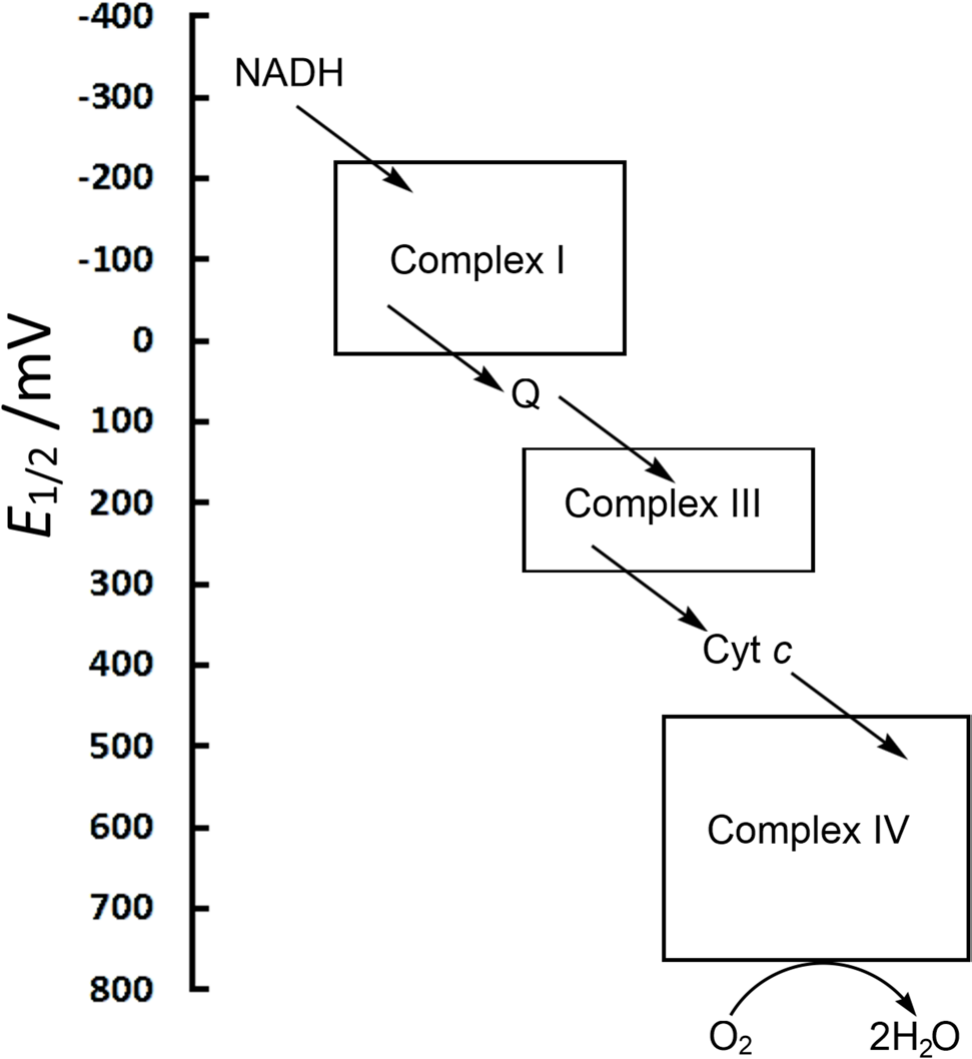
ETC of the mitochondria, showing the cascade of electrons from NADH to O_2_

The entire electron transport chain of the mitochondria features electron donors and acceptors whose midpoint reduction potential, *E*_1/2_, gradually increases, providing the thermodynamic driving force for electron transfer (Fig. 9).

The control of *E*_1/2_ is clearly vital in constructing a viable ETC, and a variety of factors are used to achieve this. The identity of the metal ion is of prime importance [14]. Hence copper proteins feature at the upper end of the biological redox range (350–800 mV for the blue copper proteins) while iron–sulfur proteins tend to feature at the lower end of the range (–700–100 mV, although the high potential iron–sulfur proteins (HiPIPs) operate between 50 and 400 mV). The iron–porphyrin-containing cytochromes occupy the middle ground (–400–300 mV). The potential of the iron–sulfur proteins depends on the oxidation states that are accessible. Thus, the HiPIPs cycle between [4Fe–4S]^2+/3+^, whereas the ferredoxins cycle between [4Fe–4S]^1+/2+^. The actual oxidation state of the individual components of the cluster is difficult to determine and clusters are probably best described as being valence-delocalised [78, 81].

The ligands that coordinate the metal ions clearly affect *E*_1/2_. Thus, replacement of two Cys ligands by two harder His ligands in the Rieske centre of complex III raises its potential to around 150 mV [82] from – 240 mV for the [2Fe–2S] centres of Complex I. A comparison of the structures of the Rieske iron–sulfur protein determined *in vacuo* using DFT with the B3LYP functional, and the structure observed in a protein from *Rhodobacter sphaeroides*, show significant differences, suggesting that the protein matrix modifies the structure of the centre, and uses this to tune its redox potential [83].

#### Complex I

**Fig. 10:**
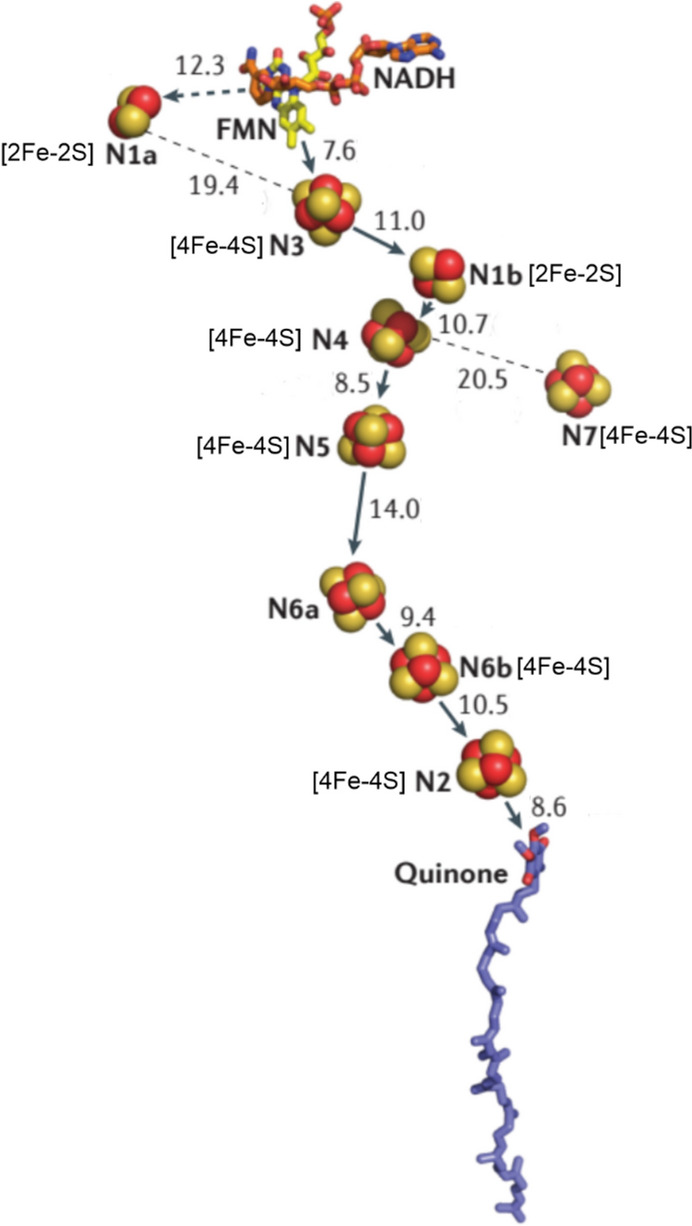
Main electron transport chain of Complex I, showing the passage of electrons from NADH, through FMN, a range of iron–sulfur centres, to Q. Edge-to-edge distances are in Å. The initial and the final step in the chain involve the transfer of two electrons, but the transfer down the iron–sulfur centres of the chain is a one electron transfer process. The diversions to clusters N1a and N7 are shown as dotted arrows. Adapted from [60, 96]

The entry point to Complex I (which consists of 45 polypeptides with a combined mass of c. 1 MDa) is the reduction of FMN to FMNH_2_ by NADH (*E*_1/2_ = –320 mV, Fig. 8), producing NAD^+^. The *E*_1/2_ value of FMN in Complex I is c. – 340 mV; this is significantly lower than that of free FMN (–207 mV) [84], indicative of how the potential of a redox-active species can be controlled and modified by the protein in which it is embedded. Passage of electrons through eight iron sulfur clusters [85, 86] (Fig. 10) ends up with the reduction of ubiquinone (coenzyme Q, *E*_1/2_ = 100 mV) to ubiquinol (QH_2_, Fig. 8). QH_2_ is the reductant used by Complex III to reduce cytochrome *c* (*E*_1/2_ = 260 mV) in the intermembrane space, which is then used by Complex IV to reduce O_2_ (*E*_1/2_ = 820 mV) to H_2_O (Fig. 9). Various proposals have been advanced for the coupling of electron transfer to proton pumping (often referred to as proton-coupled electron transfer, PCET) [58, 87–94].

The iron–sulfur clusters N3, N4 and N6a (Fig. 10) have *E*_1/2_ values of c. – 250 mV, while N1b, N5 and Nb6 have somewhat lower potentials because of electrostatic interactions with the neighbouring reduced clusters. (Cluster N1a has a potential of – 380 mV as determined potentiometrically [84].) This results in a so-called “roller-coaster” profile along the ETC which is thought to control the rate of electron transfer [95]. The terminal cluster N2 has the highest potential, c. – 100 mV, as expected for the terminal cluster of the chain. Cluster N5 has one His and three Cys residues. The long distance between it and N6a means it is a bottleneck in the ETC and given this, and the fact that it is surrounded by charged and polar residues, it has been speculated [60] that it may sense the state of the downfield clusters and hence is the gate keeper to the rate of electron flow. The reduction of quinone triggers a series of conformational changes in the Complex which lead to the ejection of protons into the intermembrane space [60].

#### A note on the iron–sulfur clusters

**Fig. 11:**
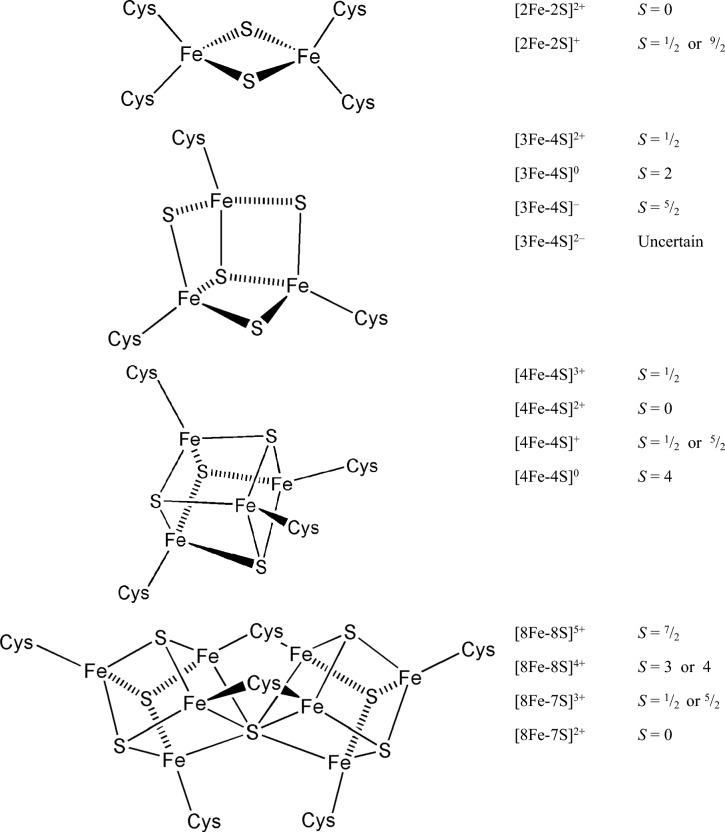
Some iron–sulfur clusters found in biological systems (adapted from [98])

Iron–sulfur clusters, comprising of (formally) Fe^3+^ and Fe^2+^, and sulfide anions, and which probably hark back to pre-biotic chemistry [97], are widely used as electron transfer agents in biology, especially in the respiratory and photosynthesis chains [98–100]. They are anchored to proteins through bonds between amino acid residues (especially Cys, sometime His) and iron. They are sensitive to oxygen so in aerobic organisms they are found buried deep inside proteins. They have a wide range of structure and spin states and span a potential range of between c. – 600 mV and 460 mV. Examples of iron–sulfur clusters, and their common oxidation and spin states, are shown in Fig. 11.

Within a protein, the clusters usually have only two accessible oxidation states and, therefore, function as one-electron transfer agents. They do serve other functions in biology, including as catalysts (such as the hydrolase enzyme, aconitase), signalling and, in birds, modulating sensitivity to magnetic sensing [101, 102]. Their physical characterisation is often achieved using a variety of spectroscopic techniques including UV–Vis, EPR, ENDOR, ESEEM, resonance Raman and Mössbauer methods [103].

Cys residues usually provide the terminal ligands of the cluster, although, as in the Rieske centre of Complex III of the mitochondrial ETC, two His residues act as ligands [104]. The protonation state of the two His ligands render *E*_1/2_ of the cluster pH-dependent and the number of hydrogen bonds between the cluster and the surrounding protein may be another way in which the redox potential of the cluster can be tuned [105]. Rhomboidal [2Fe–2S] clusters coordinated by Cys have *E*_1/2_ values that are largely independent of pH as their ligands are always negatively charged within the normal physiological pH range. Appropriate QM/MM calculations provide a way of at least estimating the redox potential of an iron–sulfur cluster and hence provide some insight into the oxidation state of the cluster [106]. In principle, [1Fe–0S], in which a single iron atom is coordinated by four Cys residues, [Fe(Cys)_4_], found in the rubredoxins of sulfur-metabolising archae, could be considered as the simplest iron–sulfur cluster. Interconversion between iron–sulfur clusters, which depends on the relative concentration of sulfide and iron, can occur (for example, between 2[Fe_2_S_2_] and cuboidal [Fe_4_S_4_] in mitochondria [107]).

Although iron–sulfur clusters are typically one-electron transfer agents, the double cubane P cluster, [8Fe–(7/8)S], found in nitrogenase, could act as a two-electron carrier [108, 109]. Some nitrogenase enzymes contain a [8Fe–9S] double cubane structure with two [4Fe–4S] subclusters bridged by a sulfide [110, 111].

The role of iron–sulfur clusters in the pathogenesis of disease is beginning to be understood [112]. High levels of ROS produced at these sites can lead to cell death; but, at low levels, ROS serve as signalling molecules. A subunit of Complex I that contains the N2 site serves as a redox-sensitive oxygen sensor that plays an important role in the homeostatic oxygen-sensing system in the pulmonary vasculature and carotid body [112].

The ability of nature to control and manipulate the *E*_1/2_ values of a metal ion centre to tune the potential for a range of functions is neatly exemplified by the iron–sulfur proteins. The Rieske clusters contain two His ligands, which will be protonated at low pH and deprotonated at high pH, p*K*_a_ being dependent on the electrostatic environment of the cluster. The decrease in *E*_1/2_ that occurs when both His ligands are deprotonated can be as high as 450 mV [113]. Iron–sulfur clusters with four Cys ligands have a low potential that arises from the effect of the four negatively charged ligands, a potential like that of a fully deprotonated Rieske centre.

The Rieske centres from the respiratory chain (cytochrome *bc*_1_ complex, or Complex III (see Sect. "Complexes III and IV")) and the photosynthesis chains (cytochrome *b*_6_*f* complex, Sect. "Electron Transport and Photosynthesis") have high, pH-dependent *E*_1/2_ values (typically around 350 mV at pH 7), whereas the ferredoxins of the bacterial oxygenases, involved in the catabolism of aromatic compounds, have lower values of *E*_1/2_ (around – 150 mV) which appear to be independent of pH.

If a redox centre, R^*n*+^, has an ionisable group (such as a ligand), or a neighbouring amino acid residue with a side chain containing an ionisable group, the uptake of an electron will couple with the uptake of a proton (Eq. 17):

17 $${\text{R}}^{n + } \cdot\cdot\cdot\cdot{\text{X }} + {\text{ e }} + {\text{ H}}^{ + } \rightleftharpoons {\text{R}}^{{\left( {n - {1}} \right) + }} \cdot\cdot\cdot\cdot{\text{XH}}^{ + }$$

The p*K*_a_ of X will depend on the oxidation state of R, i.e., p*K*_red_ > p*K*_ox_ because of the electrostatic interaction between R and X [88, 89, 114]. If *E*^o^_1/2_′ is the reduction potential in the high pH limit, then for a one-electron process, Eq. 18 applies:

18 $$E_{1/2} = E_{1/2}^{o^{\prime}} + \frac{RT}{F}\ln \left( {\frac{{1 + [{\text{H}}^{ + } ]/K_{{{\text{red}}}} }}{{1 + [{\text{H}}^{ + } ]/K_{{{\text{ox}}}} }}} \right)$$

**Fig. 12:**
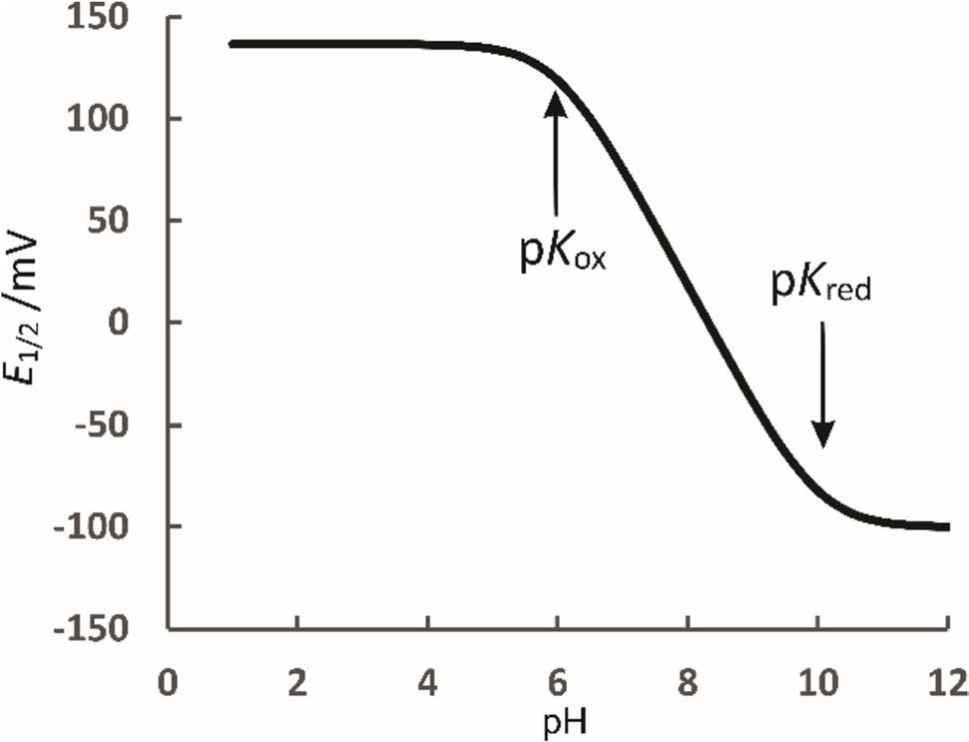
pH dependence of *E*_1/2_ if reduction is coupled to the ionisation of a neighbouring functional group, or a ligand of the redox centre. (For illustration, values of *E*^o^′ = – 100 mV in the alkaline limit, p*K*_ox_ = 6 and p*K*_red_ = 10 are used.)

This gives a sigmoidal response of *E*_1/2_ with pH, illustrated in Fig. 12 with arbitrary values of *E*^o^_1/2_′ = –100 mV, p*K*_ox_ = 6 and p*K*_red_ = 10. In this case, *E*_1/2_ is virtually independent of pH below pH 5 and above pH 11 and has a maximal dependence on pH of – 2.303(*RT*/*F*)/pH at pH 8, midway between the values of p*K*_ox_ and p*K*_red_.

An example is the pH dependence of the type-1 copper site of amicyanin [115], the primary electron acceptor of methylamine hydrogenase [116]. The site of protonation is one of the His ligands of the copper centre. In this system p*K*_ox_ ≤ 3.2 and p*K*_red_ = 6.3. This can be readily extended to systems that have more than one site of protonation which are coupled to the uptake of an electron. An example is the Rieske centre from *Thermus thermophiles* where both His ligands of the [2Fe–2S] cluster have p*K*_a_s that depend on the oxidation state of the iron–sulphur centre [113].

Protein-film voltammetry has been used to compare the pH-dependence of the potentials of Rieske proteins from *Rhodobacter sphaeroides* (*Rs*Rp), a high-potential, pH-dependent Rieske protein from a ubiquinol oxidizing enzyme; *Thermus thermophilus* (*Tt*Rp), a menaquinol oxidizing cytochrome *bc*_1_ which, therefore, has a lower, pH-dependent reduction potential since menaquinol is more readily oxidised than ubiquinol; and the low-potential Rieske ferredoxin from a strain of *Burkholderia* (BphF) [82]. The technique relies on examining the electrochemical behaviour of a protein immobilised on an electrode [114]. The pH dependence arises from the coupling of electron transfer to deprotonation of the His ligands. The lower potential of BpHF arises from the absence of a cluster of hydrogen bonds to neighbouring Ser residues. The study showed that the potential of all three centres is in fact pH-dependent, but p*K*_a_s of the His ligands in BphF are much higher than in the other two [9.8(2) and 11.5(4) compared to 7.6(1) and 9.6(1) in *Rs*Rp and 7.9(2) and 9.7(1) in *Tt*Rp], thus rendering its behaviour essentially pH independent under normal physiological conditions.

The potential depends on electrostatic effects between the iron–sulfur centre and the surrounding protein, which affects *E*_1/2_ and p*K*_a_ of the centre’s His ligands, and also the degree of coupling between the oxidation state of the ligand and the protonation state of its His ligands; the p*K*_a_ of the oxidised state may be up to five units lower than that of the reduced state [113, 117]. These effects provide nature with the tools needed to fine tune the *E*_1/2_ values of the Rieske centres to be able to fit them into electron transport chains.

#### Complexes III and IV

The reaction catalysed by Complex III is given by Eq. 19, and that catalysed by Complex IV by Eq. 20:

19 $${\text{QH}}_{{2}} + {\text{ 2cyt}}c^{{{3} + }} + {\text{ 2H}}^{ + }_{{{{in}}}} \to {\text{ Q }} + {\text{ 2cyt}}c^{{{2} + }} + {\text{ 4H}}^{ + }_{{{{out}}}}$$

20 $${\text{O}}_{{2}} + {\text{ 4cyt}}c^{{{2} + }} + {\text{ 8H}}^{ + }_{in} \to {\text{ 2H}}_{{2}} {\text{O }} + {\text{ 4cyt}}c^{{{3} + }} + {\text{ 4H}}^{ + }_{out}$$

In some organisms (actinobacteria, for example) Complex III and Complex IV are associated to form a supercomplex [118]. Complex II (succinate–quinone oxidoreductase) is another entry point of electrons into the electron transport chain.

**Fig. 13:**
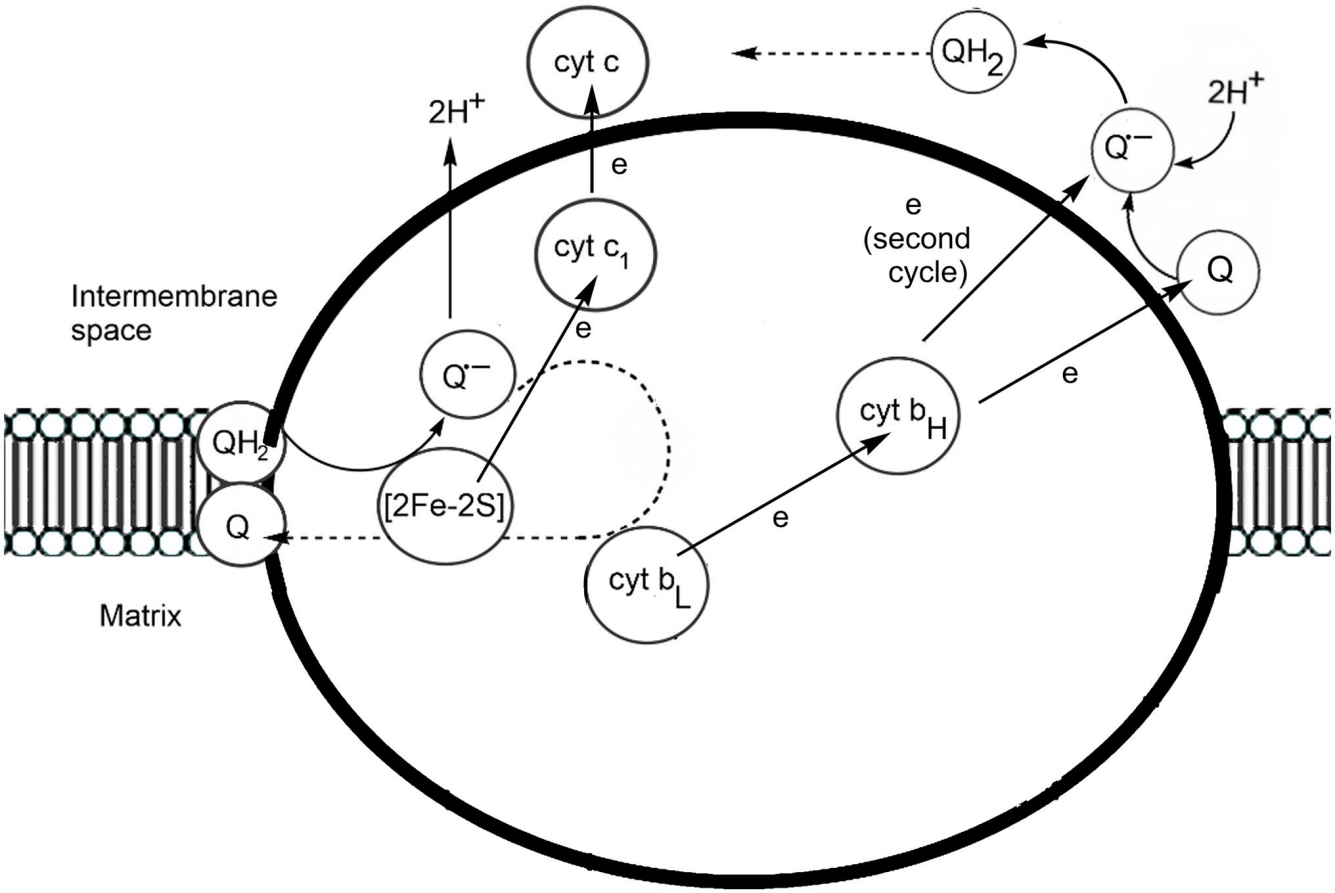
Schematic of the reactions of Complex III

As mentioned, one of the challenges that living organisms face in an oxidising environment is the formation of ROS (Sect. "Reactive Oxygen (and Other) Species"). The functioning of Complex III of the mitochondrial ETC, the function of which, thanks to comprehensive mathematical modelling [119], is known in some detail, provides an illustration of the problem. An outline of the mechanism of Complex III is shown in Fig. 13 [118, 120, 121]. During its normal state, Complex III is in a low ROS-producing steady state. However under a deficiency of oxygen condition (anoxia), the complex switches to a different steady state in which ROS production is high [119]. This steady state persists for some time even after the return of the system to oxic conditions.

The reaction of Complex III begins with the [2Fe–2S] centre of the Rieske iron–sulfur protein accepting an electron from QH_2_, producing a semiquinone radical Q^•–^; 2 protons are released into the intermembrane space. The Rieske protein reduces cyt *c*_1_, which passes the electron to cyt *c*. Q^•–^ passes an electron to the low potential cyt *b*_L_ of cytochrome *b*, which then reduces the high potential cyt *b*_H_. Q, bound on the matrix side, receives an electron from cyt *b*_H_, generating Q^•–^which, on a second cycle, is reduced by another electron from cyt *b*_H_, generating QH_2_, the binding of which on the intermembrane space side leads to another cycle. The semiquinone radicals generated, however, can react with O_2_, producing O_2_^•–^ [122].

**Fig. 14:**
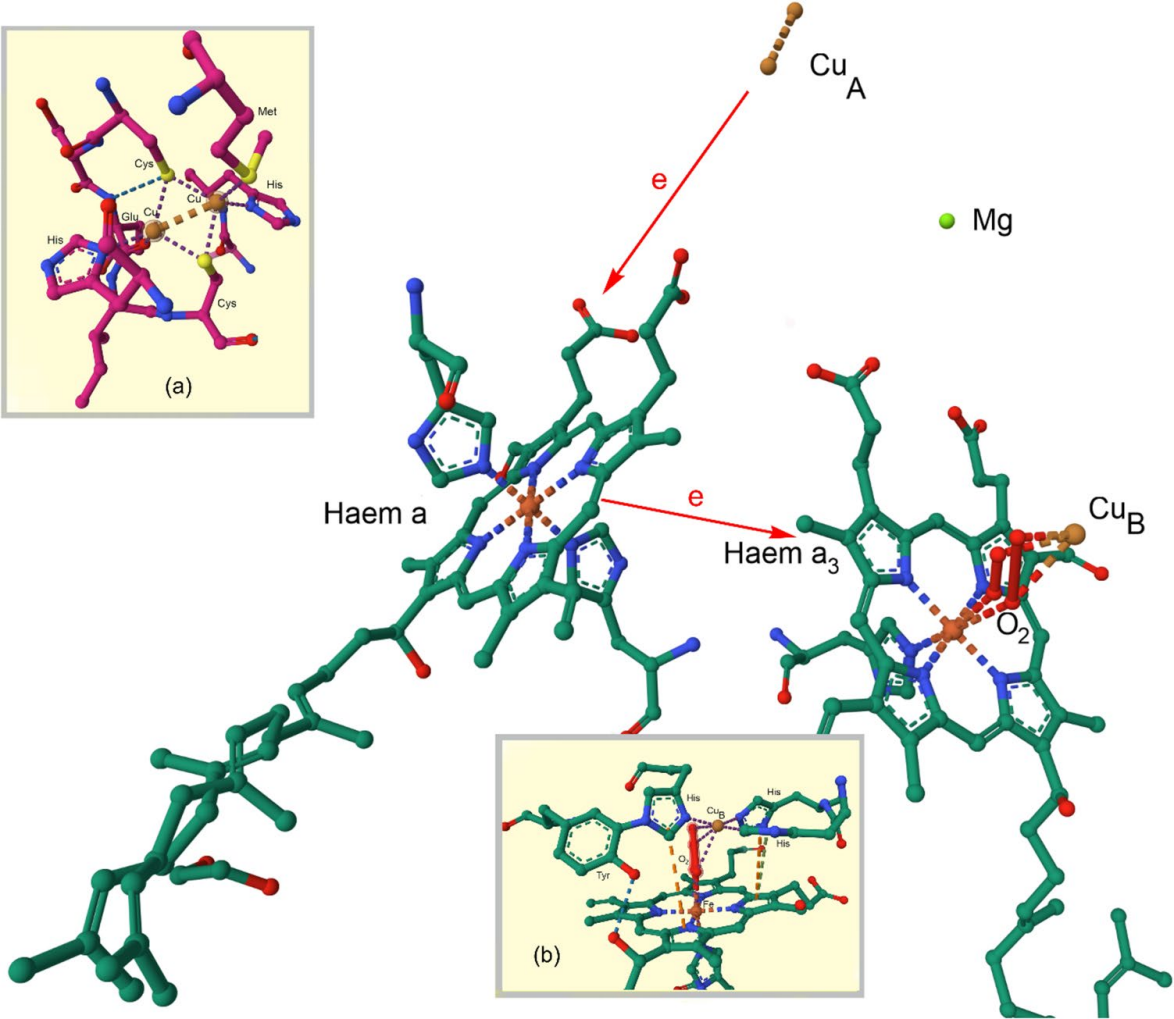
Flow of electrons through Complex IV, from the dicopper centre to haem *a*, and then to haem *a*_3_ with nearby Cu_B_. The structure shown is from bovine heart cytochrome* c* oxidase in the fully oxidized state (PDB code 5XDQ) [124]. Bound O_2_ (probably best described as peroxide), disordered over two positions, is coordinated by iron of haem *a*_3_ and Cu_B_. There is a catalytically important and highly conserved Tyr reside that is covalently linked to one of the His ligands of Cu_B_ (see Fig. 15, species P_M_). Insert (**a**) shows the structure of the dicopper centre and insert (**b**) O_2_ bound to the haem *a*_3_-Cu_B_ site. Fe···O 2.35–2.42 Å; O···Cu 2.18–2.24 Å

The haem–copper oxidases are a superfamily of enzymes that catalyse the consumption of O_2_. There are three sub-classes (A, B, C); class A includes cytochrome *c* oxidase in mitochondria and some bacteria. The B- and C-type haem–copper oxidases are found only in bacteria and archae. Mitochondrial cytochrome *c* oxidase (Complex IV) contains a dicopper centre, coordinated by Cys, Met, His and Glu residues, that receives an electron from cytochrome *c* (redox potential of c. 250 mV). This is transferred to haem *a* which is turn passes it on to a haem *a*_3_ (haem *b* in some reductase families) which has a nearby Cu_B_ centre, coordinated by three His residues (redox potential of 800 mV). O_2_ straddles Cu_B_ and Fe of haem *a*_3_ when bound, in effect being reduced by two equivalents to O_2_^2−^ by Fe^2+^ and Cu^+^ (Fig. 14). Under physiological conditions, the reduction of O_2_ to 2H_2_O yields c. 210 kJ but requires a catalyst because the reductions of O_2_ (*S* = 1) by an organic substrate (*S* = 0) is a spin-forbidden process. Holding the products of the reduction of O_2_ tightly between the two metal ions minimises the leaking of ROS. This strategy is also employed by other enzymes, including multi-copper oxidases such as CueO from *E. coli* which uses a trinuclear cluster as the site of O_2_ reduction [123] (see Sect. "Multi-Copper Oxidases").

**Fig. 15:**
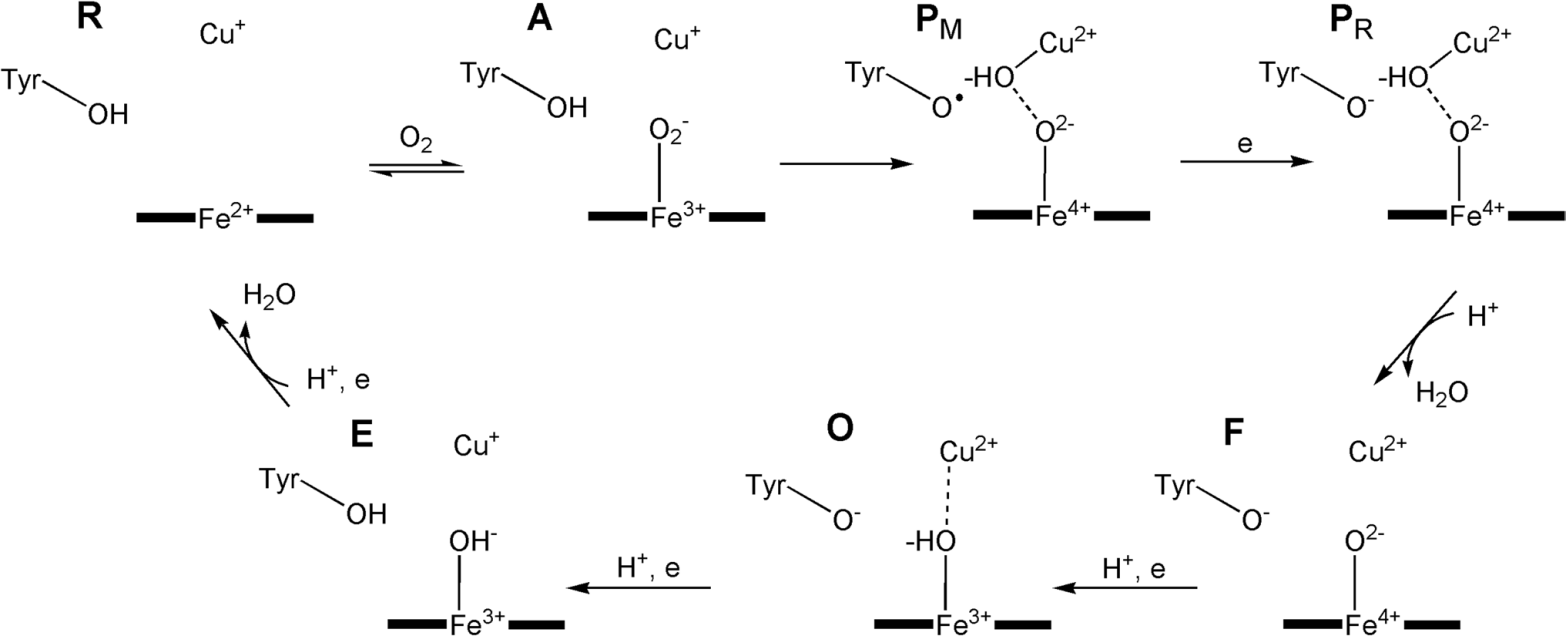
Simplified version of the catalytic cycle of cytochrome *c* oxidase (see [125, 126] for greater detail). The uptake of each H^+^ at the cytochrome *a*_3_–Cu_B_ site is coupled to the pumping of a H^+^ across the membrane. Note the formation of an oxo wall-allowed oxyferryl complex. The tyrosyl radical (in species P_M_) plays an important role in the process

**Fig. 16:**
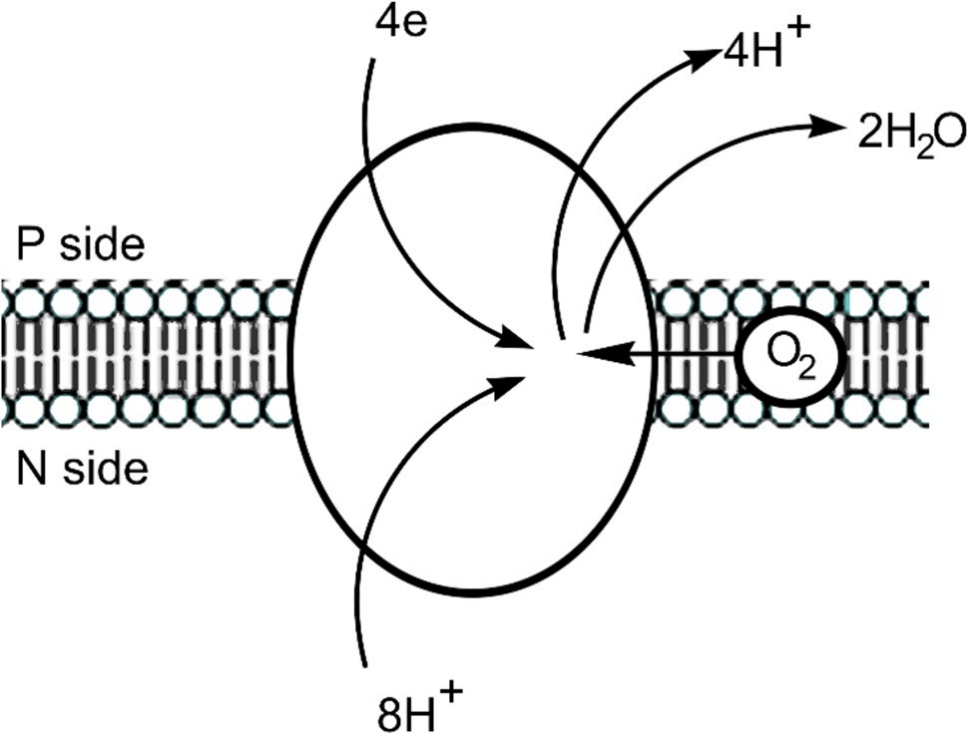
Coupling of O_2_ reduction to proton pumping across the inner mitochondrial or the bacterial cell membrane into the intermembrane space

The entire catalytic cycle is usually described as consisting of six stages, **R**, **A**, **P**, **F**, **O** and **E** [125], although, drawing on the results of DFT calculations, intermediates to these stages have been suggested [126] (Fig. 15). The formation of a tyrosine radical during turnover is important. Each uptake of a proton by the catalytic cytochrome *a*_3_–Cu_B_ centre is (to a first approximation, see [125]) coupled to the transfer of a proton across the membrane from the high pH or negative, N, side to the low pH, or positive, P, side. A schematic representation is shown in Fig. 16 (see also Fig. 1). The stoichiometric efficiency of the translation of the c. 210 kJ release to ATP synthesis is estimated to be between 75% and 90% [125, 127].

Two channels, called the D (named after a conserved Asp) and K (conserved Lys) channels, and possibly a third, H (involving haem *a*), exist for pumping protons from the N surface to the P side, involving amino acids with protonatable side chains and water molecules that transfer the protons using a Grotthuss-type mechanism [128–130]. It is thought [131] that there is direct electrostatic coupling between the transferred electron and the translocated proton, probably a consequence of the dependence of the p*K*_a_ of an ionisable group on the oxidation state of the haems (see Sect. "A note on the iron-sulfur clusters" for discussion of this principle). Synthetic systems have been prepared which display proton-coupled electron transfer via a Grotthuss-type mechanism over distances of up to 16 Å [132]. In cytochrome *c* oxidase, the coupling appears to rely on conformational changes of the complex that occur during electron transfer [133]. There is evidence that the K channel provides the route for one or two of the protons used in reduction of O_2_ to H_2_O, while the D channel provides the route for two or three of the substrate protons, and all four translocated protons [134].

### *Electron* transport and photosynthesis

Another example of an elaborate electron transport chain occurs in the structures used for photosynthesis by green plants, algae and cyanobacteria. In its simplest terms, the reaction that is catalysed using radiant energy is the conversion of CO_2_ to glucose (Eq. 21):

21 $${\text{6CO}}_{{2}} + {\text{ 6H}}_{{2}} {\text{O }} \to {\text{ C}}_{{6}} {\text{H}}_{{{12}}} {\text{O}}_{{6}} + {\text{ 6O}}_{{2}}$$

Even for the most energy-efficient plants, the maximum conversion of solar energy to biomass is only some 6% [135]. (Perhaps the abundance of solar radiation has not incentivised evolution to be more efficient!) Nevertheless, mimicking nature has prompted a great deal of research into photovoltaic devices (for example, [136–146]).

**Fig. 17:**
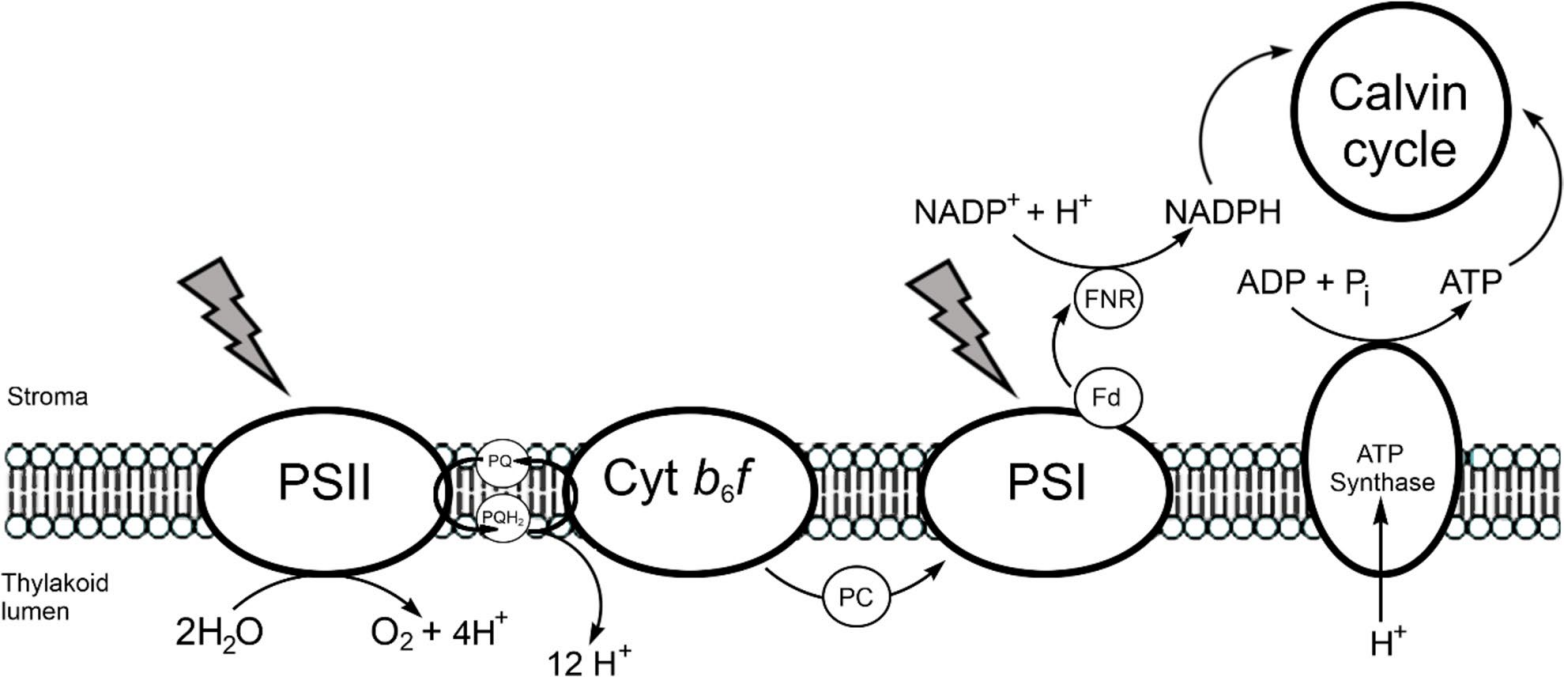
Transfer of electrons from H_2_O to NADP^+^. PQ, plastoquinone; PQH_2_, plastoquinol; PC, plastocyanin; Fd, ferredoxin; FNR, ferredoxin–NADP^+^ reductase. Many variations of this basic scheme exist (see, for example, [150])

**Fig. 18:**
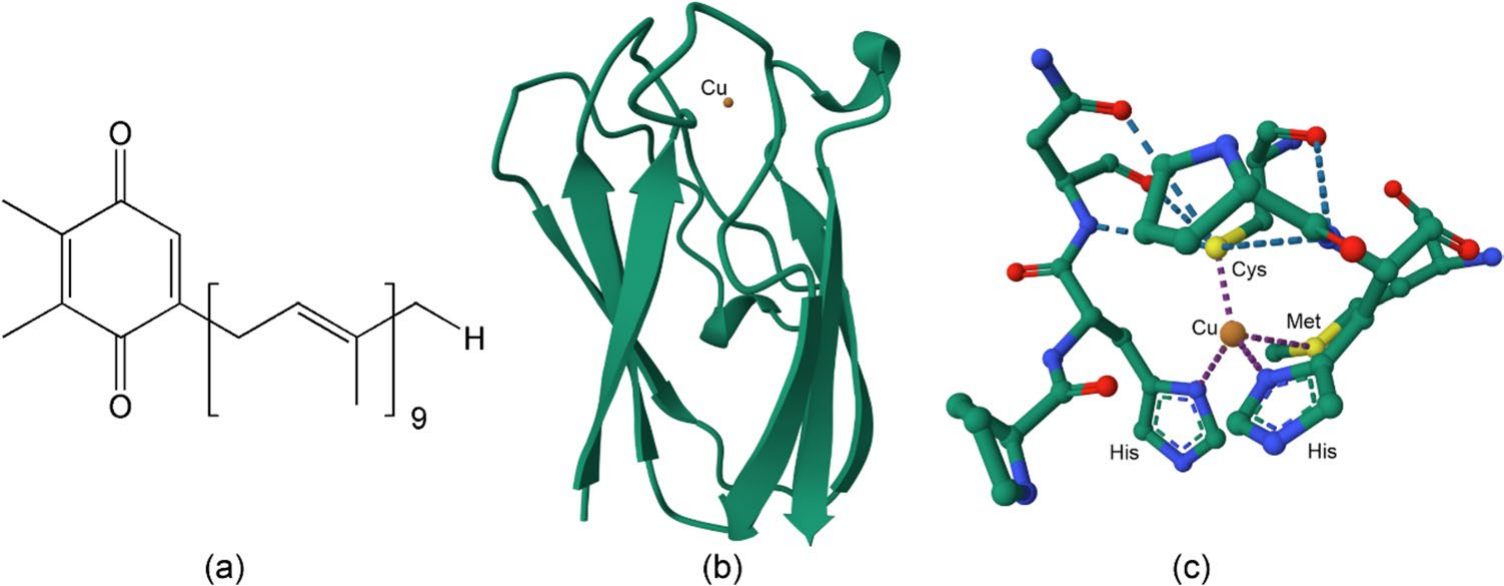
Free electron carriers of photosynthesis. (a) Plastoquinone. **b** Plastocyanin (oxidised PC from *Synechococcus*, PDB code 1BXU [151]). The coordination environment of copper in 1BXU is shown in (c)

Radiation supplies the energy for the flow of electrons from H_2_O to NADP^+^ utilising two photosystems, PSI and PSII in series, embedded in the thylakoid membrane (Fig. 17). The free electron carriers are plastoquinone and a copper-containing protein, plastocyanin (Fig. 18). In the process, protons are pumped into the lumen and the resulting proton-motive force is utilised to synthesise ATP.

**Fig. 19:**
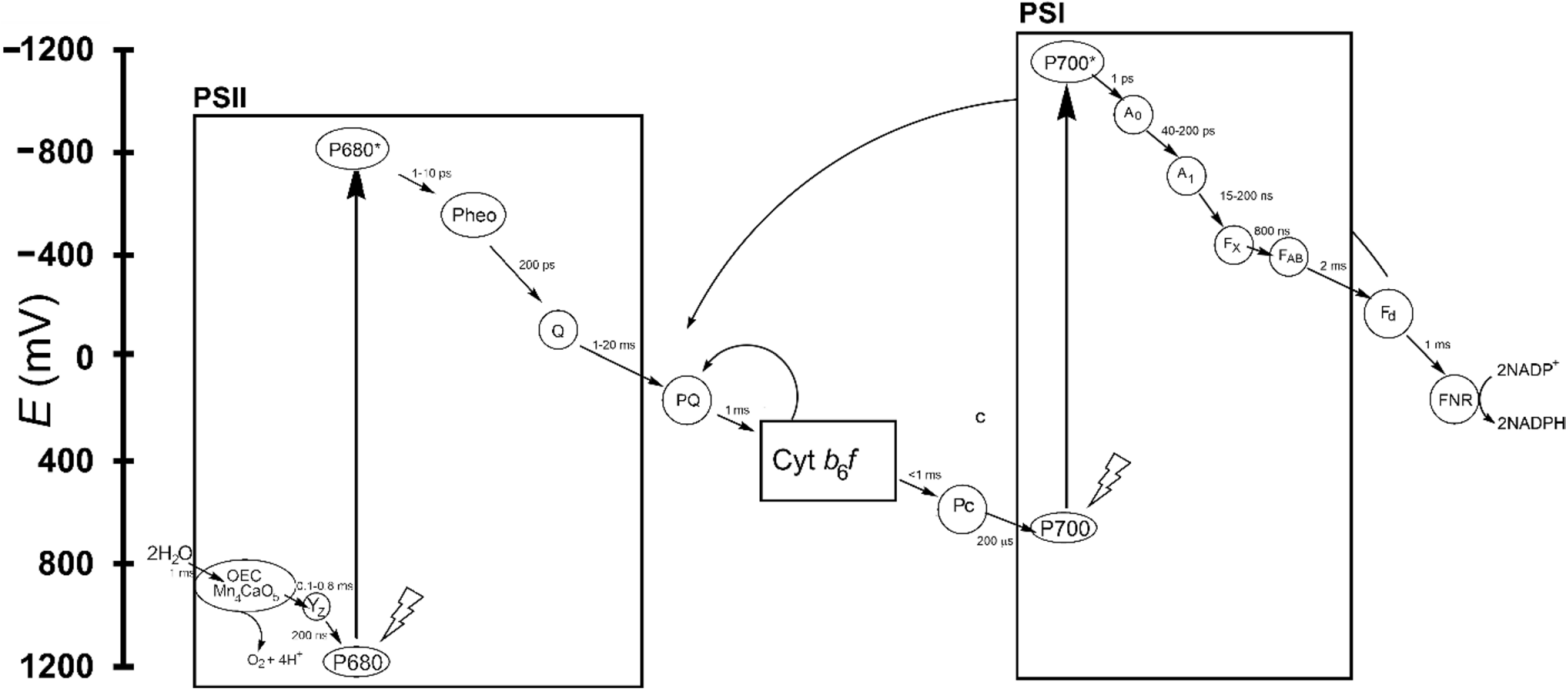
Outline of the Z scheme for linear electron transport (LET) from H_2_O to NADP^+^, adapted from [152, 153]. Approximate times for each step are given. Cyclic electron transport (CET) occurs from ferredoxin (F_d_) to the plastoquinone pool (PQ). There is a second CET cycle, called the Q cycle, of the Cyt *b*_6_*f* complex which cycles electron back to PQ. Abbreviations: OEC, oxygen evolving complex. Y_Z_, tyrosine. P680, the reaction centre of photosystem II (PSII). P680*, excited P680. Q, plastoquinone electron acceptor. The rate determining step in the entire cycle is the rate of reduction of plastoquinone [154]. PQ, plastoquinone pool. Pc, plastocyanine. P700, the reaction centre of photosystem I (PSI). P700*, excited P700. A_0_, primary electron acceptor of PSI. A_1_, secondary electron acceptor of PSI. F_X_, F_A_ and F_B_, iron–sulfur centres. FNR, ferredoxin–NADP^+^ reductase

A variety of sophisticated mechanisms exist to control the rate of electron transfer so as to minimise the formation of ROS when the radiant energy flux is greater than the rate of the metabolic consumption of ATP and NADPH [147]. There is also a quenching process whereby excess radiation not required for electron transport is dissipated as heat [148]. A schematic representation of the so-called Z scheme [149] for photosynthesis is shown in Fig. 19.

#### Photosystem II

The overall process of photosynthesis begins with Photosystem II [155]. PSII consists of two functional parts: the reaction centre (RC) which is highly conserved in organisms that perform oxygenic photosynthesis, and the antenna complexes which collect the radiant energy needed to drive the endothermic reaction given in the following equation:

22 $${\text{2H}}_{{2}} {\text{O }} + {\text{ 2PQ }} + h\upsilon \to {\text{ O}}_{{2}} + {\text{ 2PQH}}_{{2}}$$

**Fig. 20:**
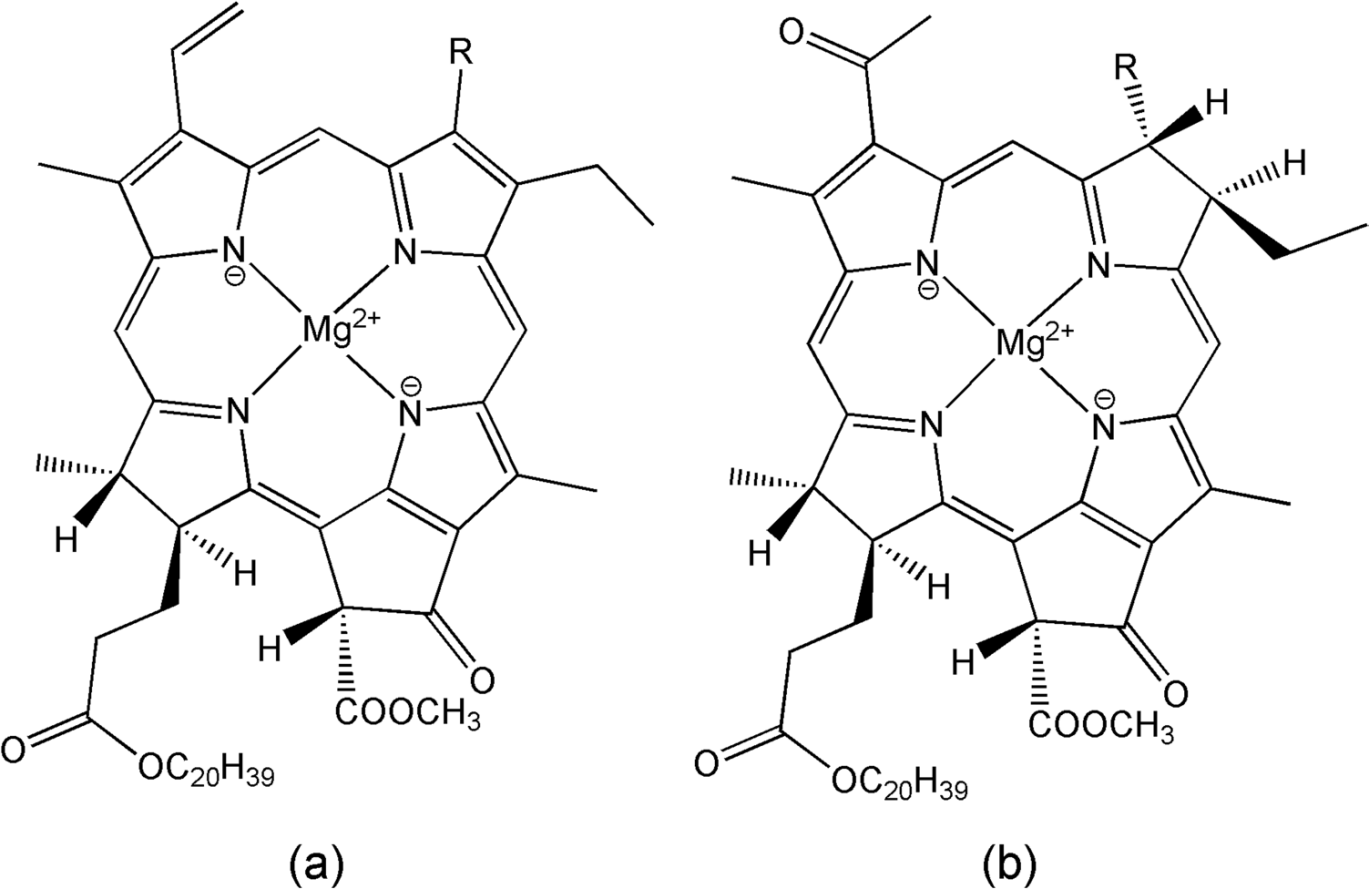
Structure of (a) chlorophylls and (b) bacteriochlorophylls. R = CH_3_ in (bacterio)chlorophyll-a and COH in (bacterio)chlorophyll-b. Mg(II) is absent in the pheophytins

The antenna complexes are highly efficient (*ε* ≈ 10^5^ M^–1^ cm^–1^) and cover a broad range of absorption wavelengths; they vary in organisational structure and composition among organisms and, since the thylakoid membrane is a dynamic system, tend to change with environmental conditions [156]. They typically consist of (bacterio)chlorophylls and (bacterio)pheophytins [157] (Fig. 20). In plants, the antenna complex is usually referred to as a light harvesting complex (LHC). The energy collected by the antenna complexes is transferred to the RC [158].

**Fig. 21:**
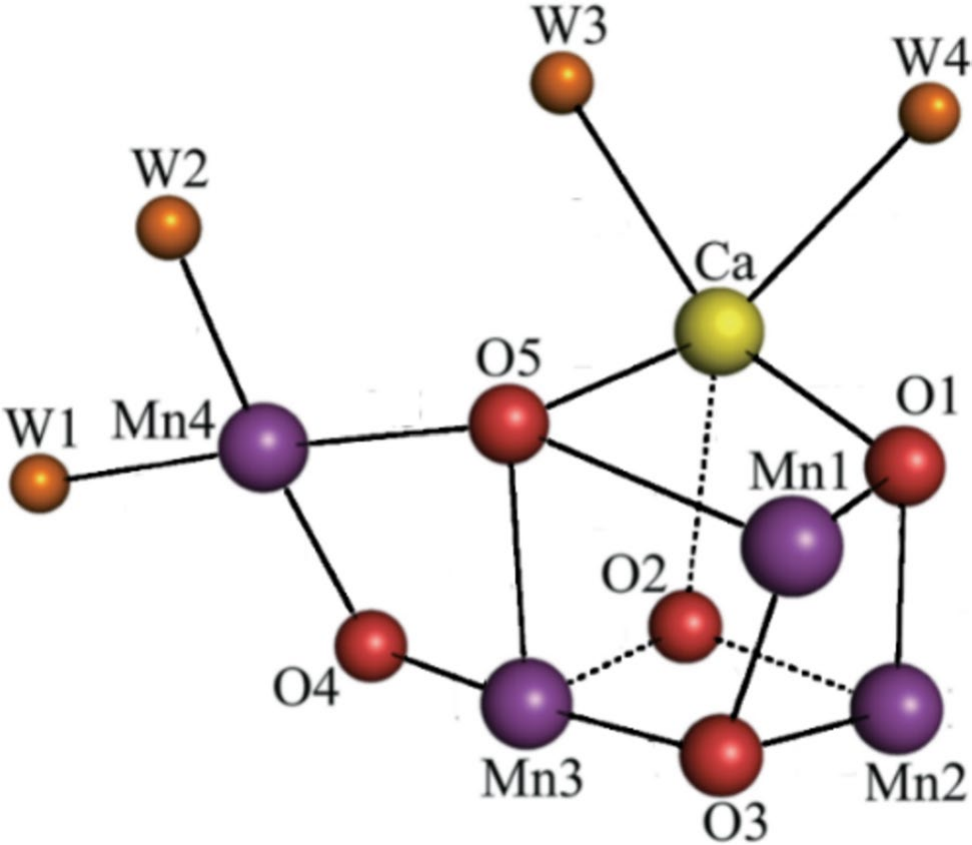
Mn_4_CaO_5_ cluster of the OEC of PSII (from [165], modified). (W = water.)

**Fig. 22:**
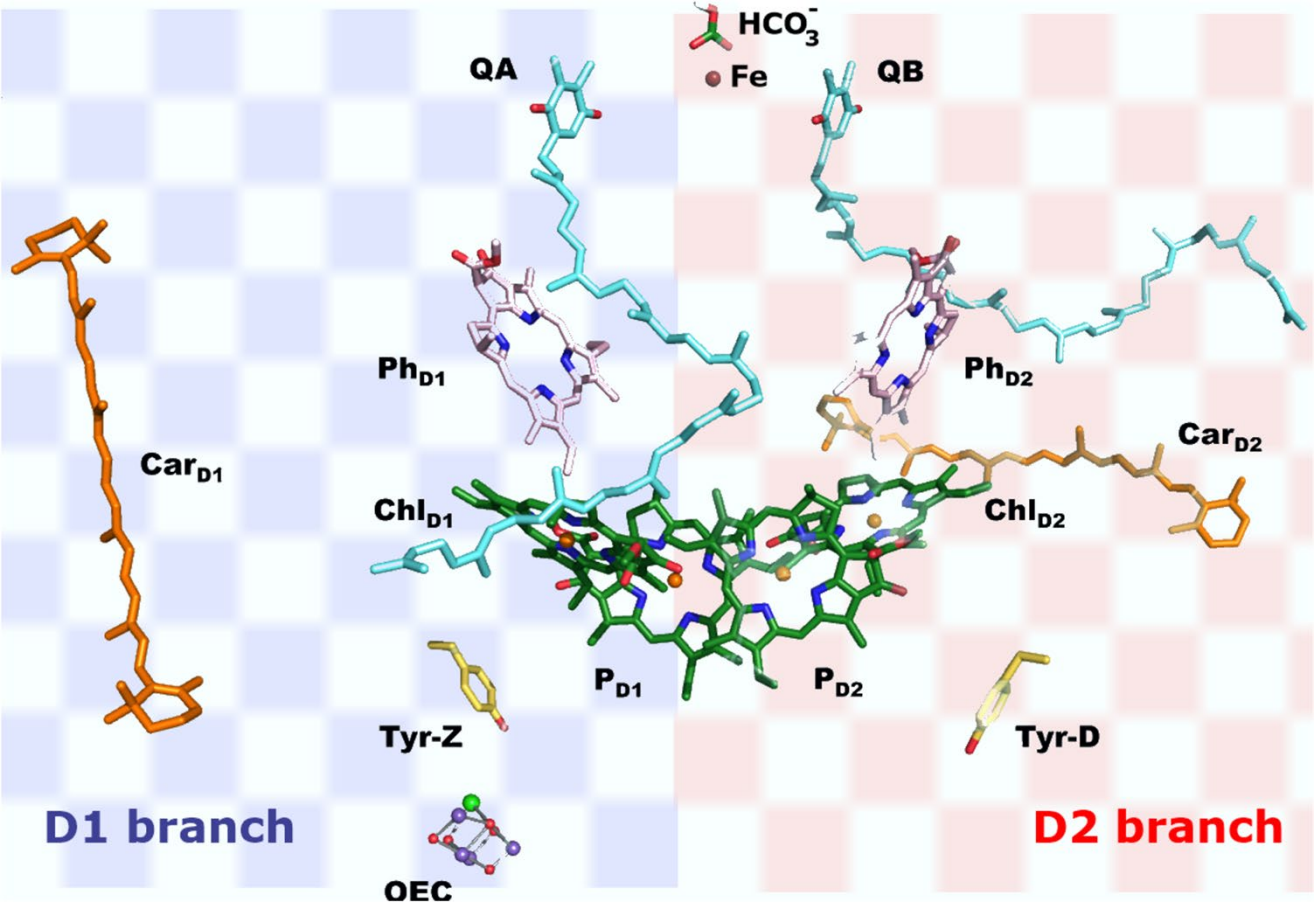
Simulation of the D1 and D2 branches of PSII (adapted from [168])

The cofactors of the RC are organised in two branches, D1 and D2. D1 contains the oxygen evolving complex (OEC), a cluster with empirical formula Mn_4_CaO_5_, that is essentially identical in cyanobacteria, algae and plants and probably dates back to the evolution of photosynthesising organisms that led to the Great Oxidation Event some 2.7 billion years ago [159–161]. The core of the OEC is a cubic Mn_3_CaO structure with an additional Mn appended through a* μ*-oxo bridge (Fig. 21). The structure is surrounded by amino acid residues of the protein including a tyrosine referred to as Tyr Y_Z_, two Cl^–^ ions, and many molecules of water [162–166]. Chloride in the OEC is required for the transfer of an electron from H_2_O to the manganese cluster; in its absence Δ*E*_1/2_ < 0 [167]. In addition to the OEC and the polypeptide chains D1 and D2, the RC consists of a chlorophyll-*a* “special pair” (P_D1_ and P_D2_), the two chlorophylls*-a* (Chl_D1_ and CHl_D2_), two pheophytins-*a* (Ph_D1_ and Ph_D2_), two quinones (Q_A_ and Q_B_), and a bicarbonate ion coordinated to a non-haem iron (Fig. 22). A carotenoid, Car_D1_ is part of the D1 branch.

In the D1 branch, charge separation occurs on Chl_D1_ (usually referred to as P680) on absorption of radiation; photo-excited P680* then reduces pheophytin Phe_D1_ which, via Q_A_ and Q_B_, leads to the reduction of a plastoquinone. P680^+^ is a strong oxidant (*E* ≈ – 1.3 V) and oxidises Tyr Y_Z_. This is the driving force for the cyclical mechanism that oxidises H_2_O to O_2_ in the OEC. The D2 branch does not contain an OEC and its function may be the protection of PSII from uncontrolled oxidative reactions and from photoinhibition [169]. However, due to thermal fluctuations, the excitation of PSII might in fact be delocalised over all the chlorophylls and the pheophytins [168, 170].

**Fig. 23:**
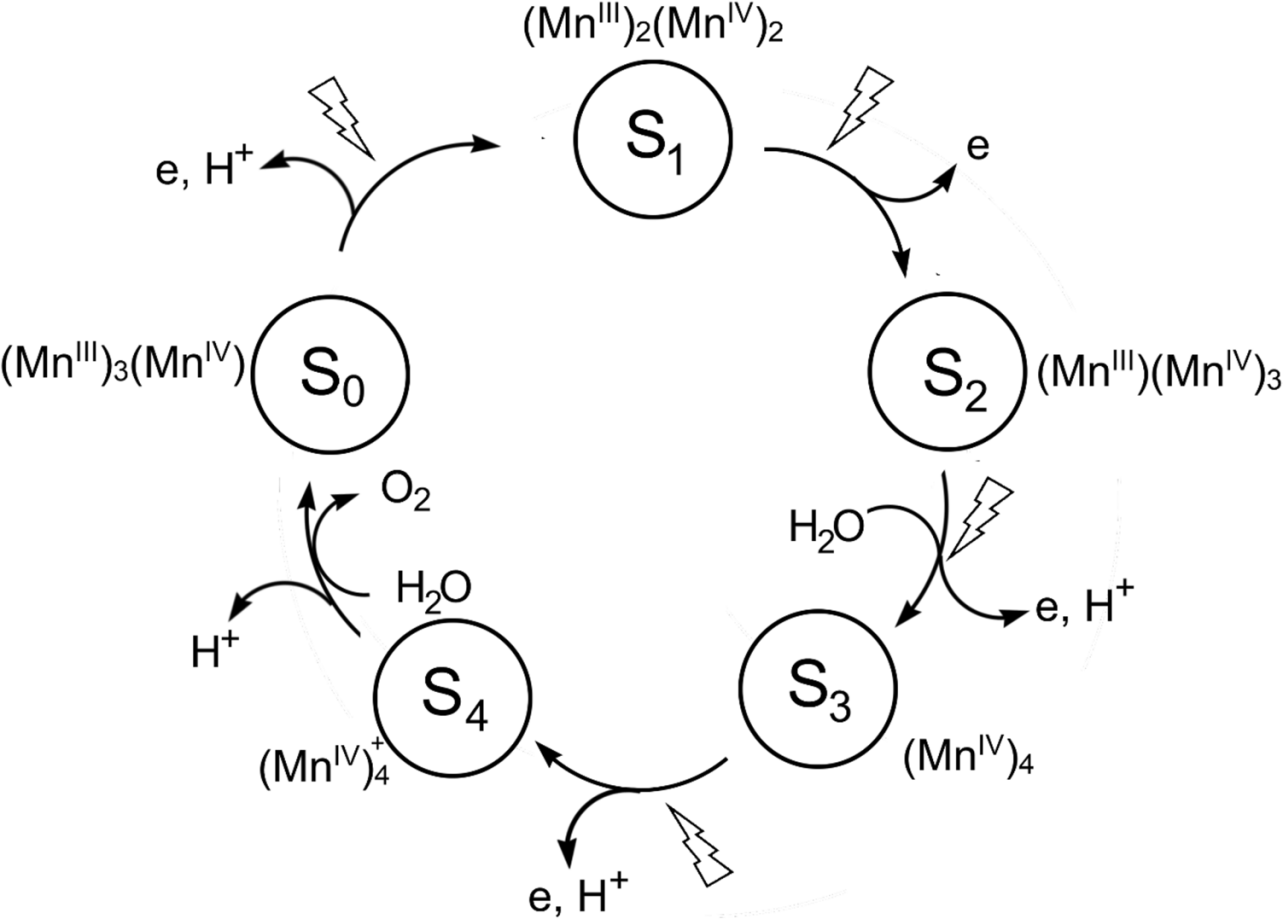
Kok–Joliot cycle of the OEC of PSII. While there is general agreement on the structures of S_0_, S_1_ and S_2_, the structures of S_3_ and S_4_ are uncertain

The cyclical mechanism that leads to the splitting of water and the evolution of oxygen is known as the Kok–Joliot cycle [171] (Fig. 23) and relies on the ability of manganese to access both + 3 and, importantly, the + 4 oxidation states. Of course, in multi-metal complexes the actual oxidation state of each metal is a moot point because of the delocalisation of the charge, as commented on before (see also [172, 173]). Therefore, the splitting of water is driven by the absorption of radiant energy by chlorophylls, pheophytins and carotenoids (as well as phycobiliproteins in cyanobacteria).

The actual mechanism of the OEC is still uncertain. Since the formulation of the Kok–Joliot cycle, there has been much discussion about the mechanism (see, for example, [153, 172–176] and references therein). We will mention three proposals.

**Fig. 24:**
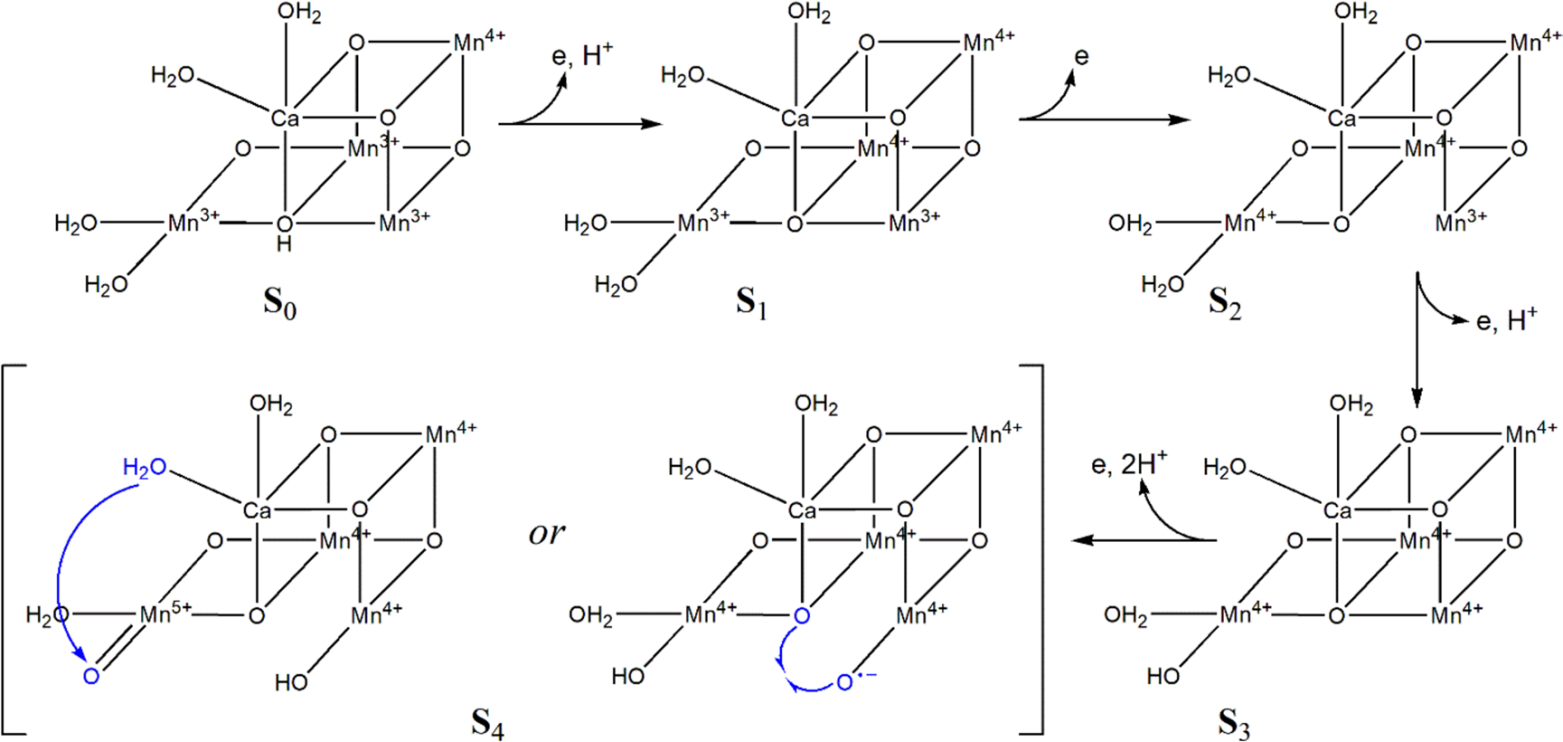
Structures of the intermediates of the OEC cycle. There is no general agreement yet regarding the structure of S_4_ or if there is an intermediate between S_2_ and S_4_. Two proposals for the formation of O_2_ by the OEC in S_4_ are shown. The occurrence of oxo–Mn^4+^ is notable. (Adapted from [177, 178])

The uncertainty arises because of the paucity of experiment evidence for the nature of the S_3_ → S_4_ and S_4_ → S_0_ transitions. As shown in Fig. 24, water acts as an attacking nucleophile on a coordinated oxo ligand; an alternative mechanism envisages the formation of O_2_ occurring through oxo-oxyl radical coupling.

**Fig. 25:**
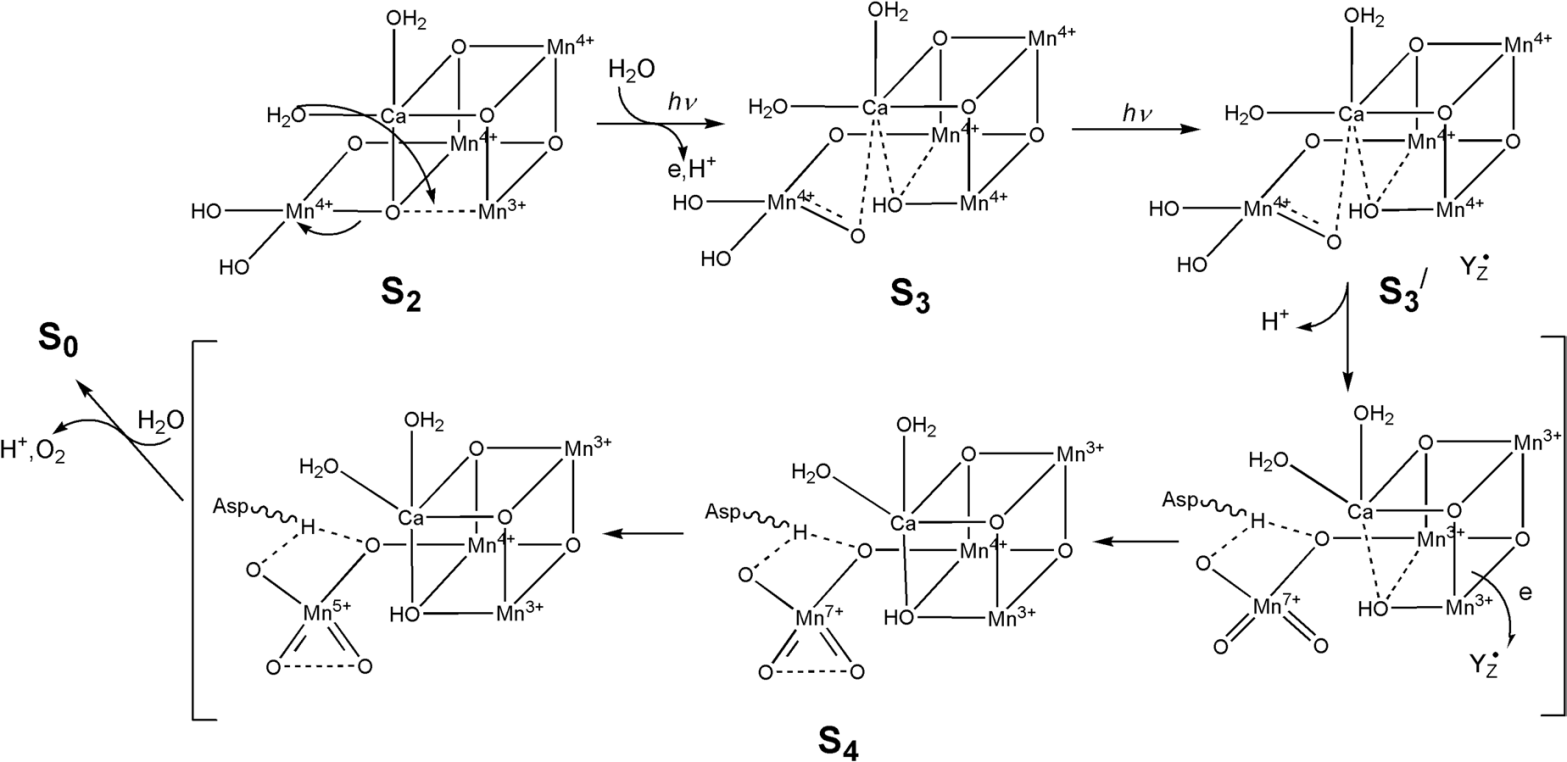
Alternative proposal [178] for the evolution of O_2_ at the OEC

An alternative proposal has been advanced relatively recently by Zhang and Sun [178]. Their proposal involves a Mn^VII^-dioxo intermediate located on the pendant Mn site. Their proposed mechanism, from S_2_ onwards, is shown in Fig. 25. The mechanism requires access to the + 3, + 4 and + 7 oxidation states of Mn, which are all well-established oxidation states of this metal [179]. As they argue, the construction of an OEC using four Mn ion allows for the accumulation of charge needed to oxidise two H_2_O molecules to O_2_. The process from S_2_ onwards is triggered by the absorption of radiation (experimentally known to occur—see Fig. 23) which produces a radical on tyrosine Y_Z_. Charge rearrangement ultimately leads to the formation and release of O_2_ which then, on uptake of H_2_O, regenerates the S_0_ state. They provide precedents from Mn chemistry in support of this proposal [178].

It is thought that the principal function of the calcium ion in the manganese–calcium cluster is to maintain a network of hydrogen bonds around the waters (or hydroxides) that are ultimately released as O_2_, or to act as a Lewis acid, accepting an electron pair on release of O_2_ [175].

#### The cytochrome b_6_f complex and a note on the blue copper proteins

**Fig. 26:**
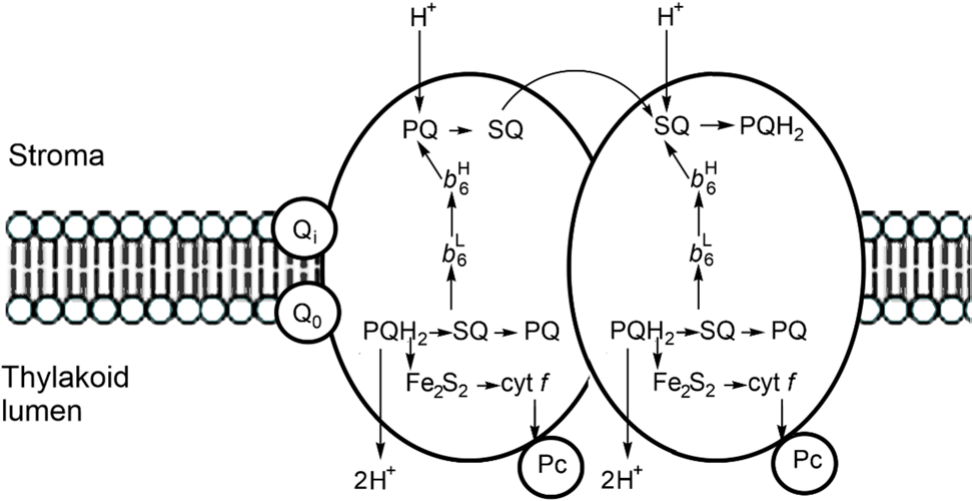
Process of electron transfer in the cytochrome *b*_6_*f* complex. PQH_2_, SQ and PQ refer to the three redox states of the quinone species in the cycle (plastoquinol, semiquinone and plasoquinone)

Between PSII and PSI lies the cytochrome *b*_6_*f* complex (plastoquinol:plastocyanin oxido-reductase), responsible for accepting electrons from plastoquinol and transferring these to the PSI complex (Fig. 26). It mediates electron transfer between PSII and PSI, oxidising PQH_2_ and reducing plastocyanin. It is a homodimer of multiple subunit monomers; the redox-active sites are a cytochrome* f* with a *c*-type cytochrome; a cytochrome *b*_6_ with a low- and high-potential haem group; and a Rieske iron–sulfur protein, [Fe_2_S_2_]. There are two quinone biding sites: Q_0_ (quinol oxidase site) and Q_i_ (quinone reductase).

The oxidation of QH_2_ occurs at the Q_0_ site which is at the interface between the *b*_6_ subunit and [Fe_2_S_2_]. Two protons are released into the thylakoid lumen. Of the two electrons released, one is transferred to Pc through [Fe_2_S_2_] and cyt *f* while the second is transferred through cyt *b*_6_^L^ and cyt *b*_6_^H^ to reduce PC at the Q_i_ site, which is on the stromal side of the membrane [180]. This bifurcated reaction can, therefore, be summarised by Eq. 23 and is coupled to proton transfer across the membrane:

23 $${\text{PQ }} \leftarrow b_{{6}}^{{\text{H}}} \leftarrow b_{{6}}^{{\text{L}}} \leftarrow {\text{ PQH}}^{ \bullet } \leftarrow {\text{ PQH}}_{{2}} \to \, \left[ {{\text{Fe}}_{{2}} {\text{S}}_{{2}} } \right] \, \to {\text{ cyt}}f \to {\text{ Pc}}$$

The reaction at the Q_i_ centre, PQ + 2e + 2H^+^  → PQH_2_, sees protons taken up from the stroma side of the membrane, so there is a net pumping of protons across the membrane, contributing to setting up a proton-motive force.

Plastocyanin is one of the so-called “blue copper proteins” which function widely as electron transfer agents and exploit Cu^2+^|Cu^+^ chemistry for this [182]. The availability of these two oxidation states within or near nature’s electrochemical window is exploited not only by electron transfer proteins, but also by catalysts such as the copper-containing oxidases and the oxygenases.

**Fig. 27:**
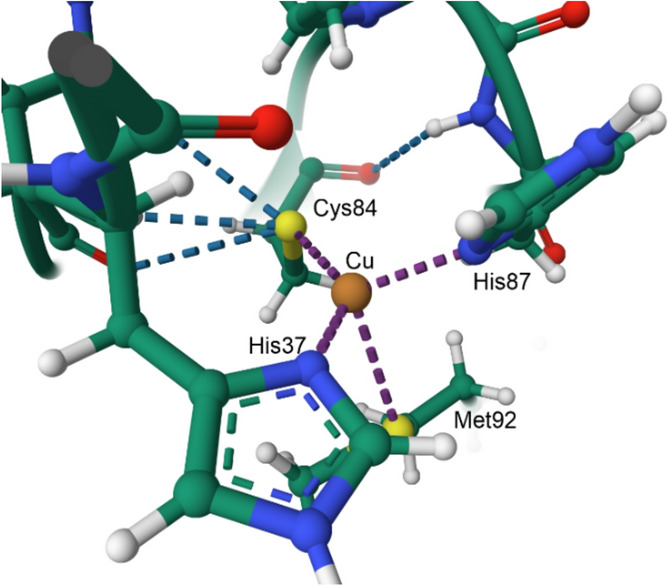
Copper binding site of polar plastocyanin (PDB 1PLC [181])

The blue copper proteins have a bright blue colour, an unusually narrow A_||_ hyperfine coupling constant in their EPR spectra, and high reduction potentials (for plastocyanin *E*_1/2_ = 370–400 mV vs SHE [183]). The copper ion is bound by Cys and two His ligands in an approximately trigonal planar geometry and the coordination sphere also contains a Met residue. The copper binding site of poplar plastocyanin (PDB 1PLC) [181] is shown in Fig. 27. The fourth ligand may be an amide O from the side chain of Asp or Glu (in the stellacyanins). Blue copper proteins such as the azurins have a five-coordinate copper (2 His, Cys, Met and a carbonyl oxygen of, for example, Gly) in a distorted trigonal bipyramidal geometry. Both the protein environment of the active site (inner and outer coordination sphere) as well as its coordination geometry tune its redox potential for efficient electron transfer between the physiological electron donor, the copper centre, and the physiological electron acceptor [184–187].

The bonding between the metal and the two His and Cys ligands is strong, with weaker bonding to Met. The typical Cu–Cys bond length is around 2.1 Å while the Cu–Met bond length is around 2.9 Å. The strong interaction between Cu^2+^ d_x_2_–y_2 and Cys S^–^ p_π_, and which, therefore, has a strong *π* character [189], leads to the intense blue colour (*ε* ≈ 5 mM^–1^ cm^–1^) that arises from a ligand-to-metal charge transfer (LMCT, S(Cys) → Cu^2+^) at 600 nm, and hence the name “blue copper proteins”. The Cu^2+^–Cys bond has significant covalent character so the unpaired electron on copper is partially delocalised onto sulfur, resulting in a significantly smaller A_||_ hyperfine coupling constant in the EPR (c. 60 × 10^–4^ cm^–1^ compared to c. 150 × 10^–4^ cm^–1^ usually observed for Cu^2+^ complexes [184]).

**Fig. 28:**
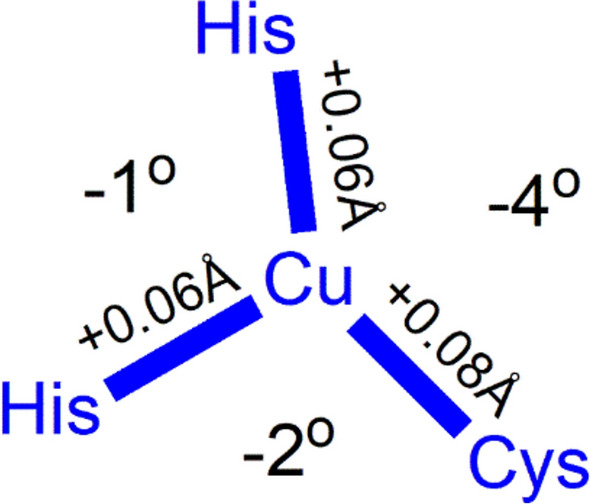
Changes in bond lengths and bond angles in the primary trigonal coordination geometry of plastocyanin that occur on reduction of Cu^2+^ to Cu^+^ [188]

The coordination geometry is intermediate between the tetrahedral geometry preferred by four coordinate d^10^ Cu^+^ and the square planar geometry favoured by four coordinate d^9^ Cu^2+^; the metal is in an entatic state [190–194], a distortion of the coordination geometry of a metal complex to approach that of the transition state of the reaction so as to minimise the reorganisation energy, *λ*, associated with the change in oxidation state, one of the barriers to electron transfer, as discussed above (Sect. "Maximising the rate of outer sphere electron transfer"). With the metal locked in an entatic state, there is a very small structural change associated with a change in the oxidation state of the copper of a blue copper protein [184, 195] (Fig. 28).

The blue copper proteins are a good example of how the structure of the protein can tune the redox potential of a metal couple [196]. Potentials for Cu^2+^|Cu^+^ range from 400 to 800 mV in laccase (2 His, Cys, Met) to 200–350 mV in stellacyanin (2 His, Cys, Asp or Glu) (cf. the standard potential of 159 mV for the Cu^2+^|Cu^+^ couple in aqueous solution [197]). The S(Cys) → Cu^2+^ LMCT at 600 nm is reflective of a ground state wavefunction with a redox active molecular orbital (RAMO) that is key to its ability of act as a rapid long range outer-sphere electron transport protein [184, 198]. The redox potential is controlled not only by the identity of the ligands, but also by the structure of the protein. For example, it has been shown that the structure of plastocyanin tunes the midpoint redox potential by controlling the extent of interaction between the Met ligand and Cu^+^ in the reduced state of the protein [199]; by perturbing the structure of the protein through mutagenesis it was demonstrated that as Met’s interaction with Cu^+^ increases, so does the midpoint redox potential.

#### Photosystem I

**Fig. 29:**
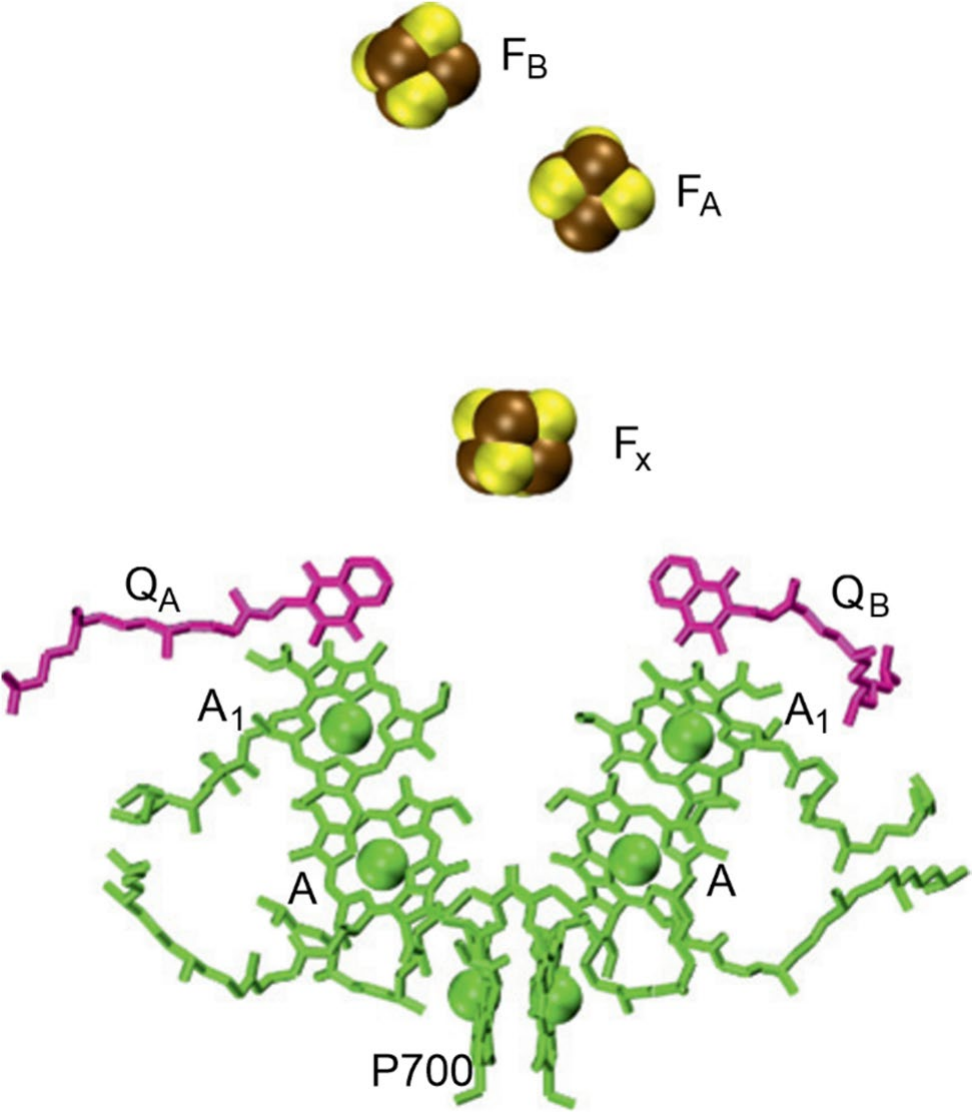
(adapted from [201]). The electron transport chain in PSI. There are two branches, A (left) and B (right). F_B_ is the final electron donor to F_d_

Photosystem I (PSI, plastocyanin–ferredoxin oxidoreductase) transfers electrons from Pc to F_d_ by absorbing radiant energy; in the process it contributes to the proton-motive force across the thylakoid membrane [200]. The electron carriers involved (Fig. 19) are P700, the primary and secondary electron acceptors A_0_ and A_1_, Q_A_ and Q_B_, and the [Fe_4_S_4_] centres, F_x_ and F_AB_. Photoexcitation occurs in the chlorophyll and carotenoids of the antenna complex. The number of these absorbing molecules varies from organism to organism (typically, between about 20 and 100). A_0_ and A_1_ are modified chlorophylls [201]. Pc (or cyt *c*_6_ is most cyanobacteria and algae) is the primary electron donor to PSI and docks on the lumen side of the complex, near P700, the photoexcitation of which prompts electron transfer. The complex extends by some 90 Å into the stromal side of the membrane and this is where F_d_ (or flavodoxin in the event of iron deficiency) docks to receive an electron. The electron transfer to F_d_ is via the iron–sulfur clusters F_A_ and F_B_, which themselves receive the electron from the iron–sulfur cluster F_x_. The electron transport chain of PSI is shown in Fig. 29 (adapted from [201])).

In response to environmental conditions, electron transfer in the photosynthesis chain is regulated by a number of kinases and a phosphatase to prevent photoinhibition through the production of ROS when the radiation flux exceeds the photosynthesis capacity of the system [202–208]. The production of ^1^O_2_ occurs principally in PSII whereas O_2_^•–^ production occurs at the acceptor site of PSI [204, 209]. There are also feedback loops. Q_i_ is involved in a feedback cycle, called the Q cycle, from the acceptor site of PSI via F_d_, FNR and possibly a haem *c*_1_ which is located near Q_i_ on the stromal side of the membrane. One of the functions of the carotenoids appears to be to quench the excited state of chlorophylls to minimise ROS production [210].

In addition to the linear electron transport from H_2_O to NADP^+^, cyclic electron transport (CET) around PSI contributes to the formation of a proton gradient across the thylakoid membrane, and hence ultimately to the synthesis of ATP [152, 211]. This CET occurs between ferredoxin (F_d_) and plastoquinone (PQ)—see Fig. 19—and is probably important in controlling the rate of LET under unfavourable environmental conditions, and hence balancing the ATP/NADPH ratio [150, 152, 212]. Another regulatory mechanism is pseudo-cyclic electron flow (PCEF) [213] in which O_2_ replaces NADP^+^ as the electron acceptor of PSI, thus regenerating the H_2_O split by PSII in what is essentially a water–water cycle.

## Examples of metalloproteins that directly couple *electron* transfer to chemical reactions

**Fig. 30:**
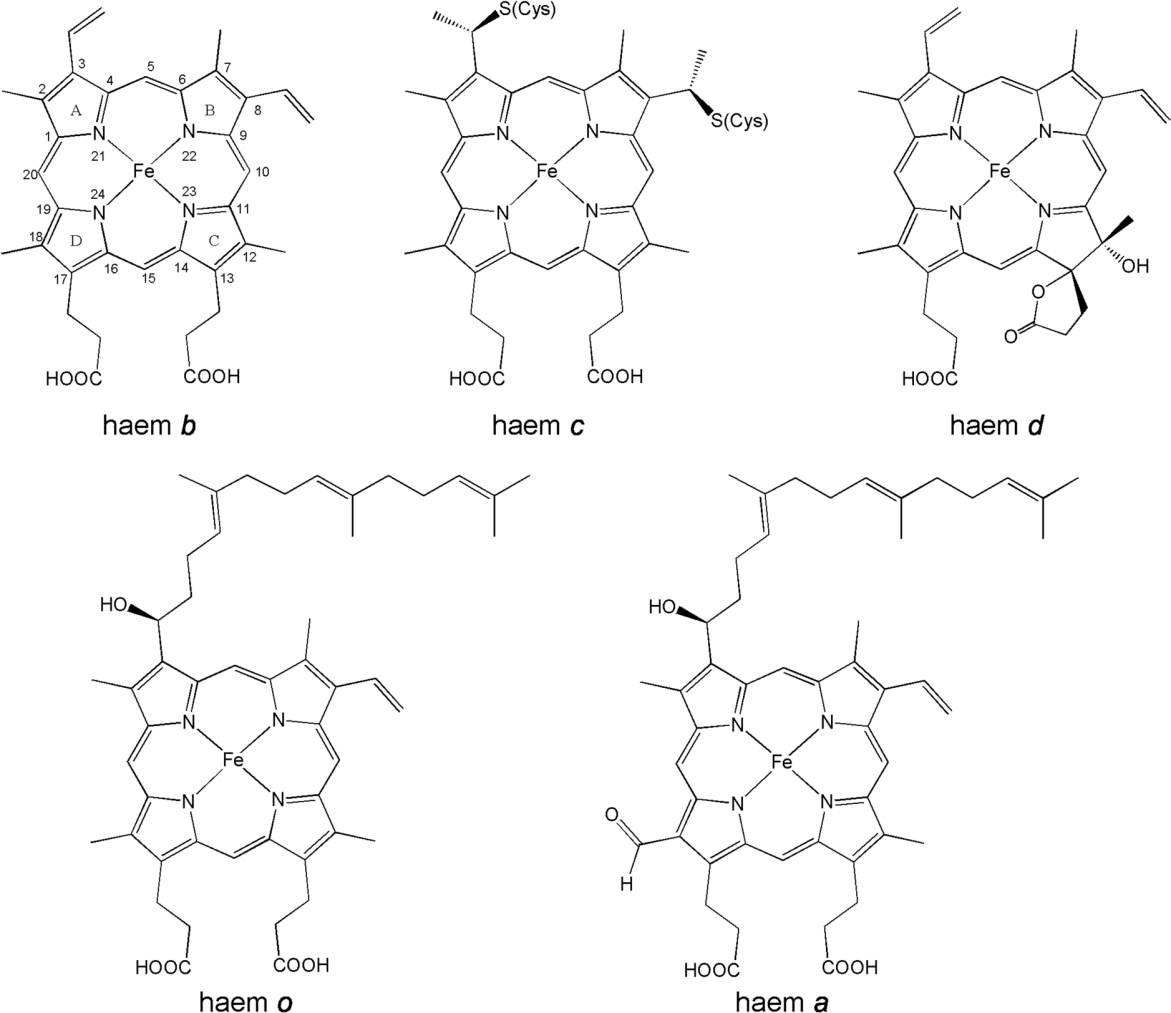
Some of the iron porphyrins, or haems, found in nature. The side chains of the tetrapyrrole, porphyrin, are different in each case. One of the double bonds of haem *d* has been reduced; this is, therefore, a chlorin rather than a porphyrin. The farnesyl group in heme *o* is important for anchoring the haem to the protein [256]

Iron plays a critical role in biology. We have already seen the widespread use of iron in the form of iron–sulfur clusters in electron transport chains (Sect. "Metalloproteins in electron transfer chains"). Another common form of biologically important iron is in the iron porphyrins (Fig. 30). Iron porphyrins fulfil many functions [214–218] including the transport (haemoglobin) and storage (myoglobin) of dioxygen [219–222]; electron transport [223–225]; as redox partners to other enzymes [226–229]; and as catalysts in the oxidases [230–233], peroxidases [231, 234–240], catalases [241–243] and the superfamily of the cytochromes P450 [231, 234, 238, 240, 244]. We have also seen their role in the mitochondrial electron transport chain and in photosynthesis; using siroheme, a porphyrin with reduced A and B rings), they are used in the reduction of nitrite [245–247] and sulfite [247–249]).

**Fig. 31:**
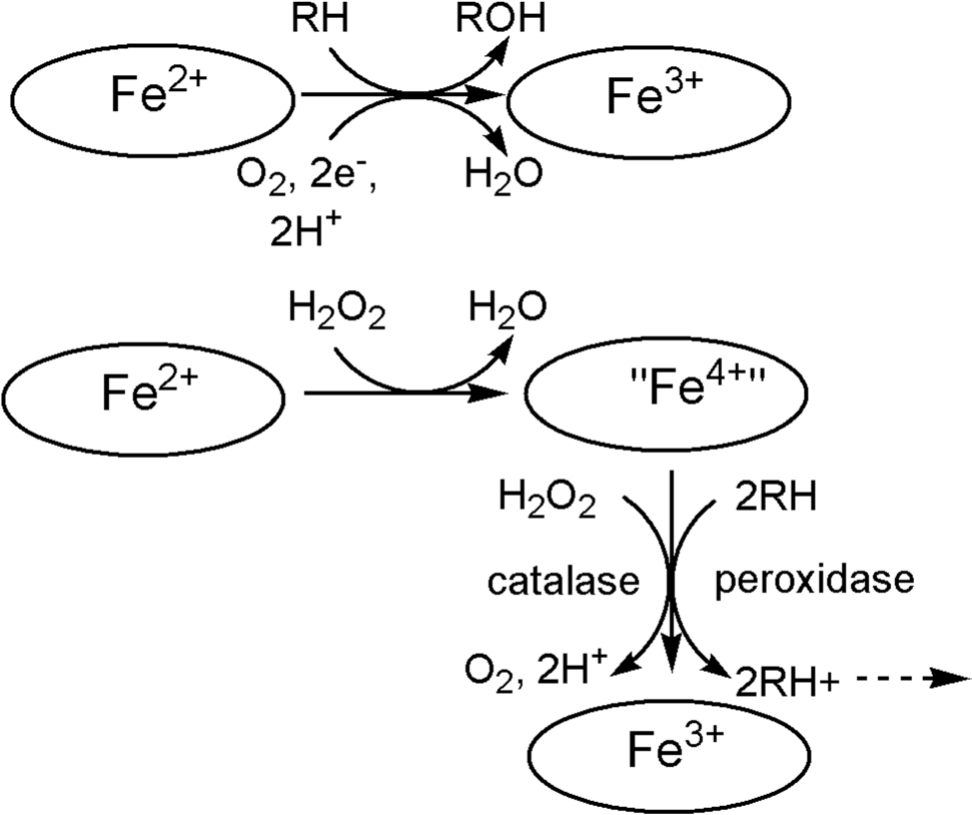
Schematic representation of the reactions catalysed by (top) the cytochromes P450 family and (bottom) the catalases and the peroxidases

We will look at some cases where electron transfer is coupled to a chemical reaction: in the case of iron, the reactions catalysed by the cytochromes P450, and those catalysed by the catalases, peroxidases, superoxide dismutases and superoxide reductases (Fig. 31). These reactions feature oxy compounds of iron porphyrins. Non-haem iron catalysts where electron transfer is coupled to a chemical reaction are also widespread. Examples include the *α*-keto glutarate enzymes [250], the Class I ribonucleotide reductases [251] and methane monooxygenases [252].

Manganese is also a relatively abundant metal ion (a crustal abundance of some 1000 ppm [253]) with a large number of accessible oxidation states. Given its relative abundance and rich redox chemistry, it is unsurprising that Mn is widely used in biological systems [254]. In mammals, Mn is required for normal amino acid, lipid, protein, and carbohydrate metabolism [255]. There are many Mn-dependent enzymes, including oxidoreductases, transferases, hydrolases, lyases, isomerases and ligases. As we have seen (Sect. "Photosystem II"), in plants, the oxygen-evolving complex (OEC) contained within the thylakoid membranes of chloroplasts is responsible for the terminal photooxidation of water during the light-induced reactions of photosynthesis.

As already mentioned, copper is another important redox-active metal. Copper is relatively abundant in the Earth’s crust (27 ppm) [257]. Whilst Cu^+^ would have been tied up as an insoluble sulfide in a reducing atmosphere, Cu^2+^ would have become available in an oxidising atmosphere as it is moderately soluble in mildly acidic solutions (*K*_sp_ of Cu(OH)_2_ = 2.2 × 10^–20^ [258]). Iron biochemistry very likely predated copper biochemistry [259].

There are many enzymes that utilise Cu^2+^|Cu^+^ chemistry [182]. The availability of these two oxidation states within or near nature’s electrochemical window is exploited by electron transfer proteins, as we have seen (Sect. "Metalloproteins in electron transfer chains"), and in catalysts such as the oxidases and the oxygenases.

Metalloenzymes provide a way around the reaction of triplet O_2_ with a singlet substrate, which is a spin-forbidden, and hence a kinetically very slow reaction [260, 261]. The abundance of O_2_ on earth after the great oxidation event and its potency as an oxidant of organic substrates required the evolution of strategies to overcome the barriers to its use as an oxidant. The answer was the activation of O_2_ at transition metal clusters with an open shell electron configuration. We have already seen this exploited by a haem and copper complex in the terminal oxidase of the mitochondrial electron transport chain (Sect. "Complexes III and IV") and the reverse reaction, the oxidation of H_2_O to O_2_, by a manganese cluster in the OEC of PSII (Sect. "Photosystem II"). Inevitably, ROS are also produced, and the catalases, peroxidases, superoxide dismutases and superoxide reductases are part of nature’s defence against oxidative stress that results from the overproduction of ROS.

### The Cytochromes P450

P450 enzymes (their name derives from their prominent absorption at ≈ 450 nm, hence *Pigment 450*) are widespread in nature and are involved in the metabolism of many organic compounds [262]; they can be traced back to primordial life [263]. For example, human cytochrome P450 enzyme, CYP1A2, is an important liver enzyme accounting for some 15% of liver P450 activity and is involved in the metabolism of many drugs and pre-carcinogens [264–266]. P450 is important for the bioremediation of organic pollutants [267, 268] and the metabolism of herbicides in plants [269]. They act as monooxygenases or mixed-function oxidases and typically use pyridine nucleotides as electron donors for the oxidation of organic substrates (Eq. 24, – RH = –CH, – NH or – SH) with the transfer of the two electrons required effected by a flavoprotein or an iron–sulfur protein [270, 271]:

24 $${-}{\text{RH }} + {\text{ O}}_{{2}} + {\text{ 2e }} + {\text{ 2H}}^{ + } \to \, {-}{\text{ROH }} + {\text{ H}}_{{2}} {\text{O}}$$

There are other enzymes that catalyse such reactions (the flavin-dependent monooxygenases such as the styrene monooxygenases, and the non-haem iron monooxygenases such as methane monooxygenase [272, 273]) but none are as widespread or as versatile (insofar as their substrates are concerned) as the P450s. In addition, the P450s catalyse many other reactions, including deaminations and dealkylations, dehalogenations, oxidative desulfurisations, epoxidations, N-oxide reductions, peroxidations, and sulfoxidations [270].

**Fig. 32:**
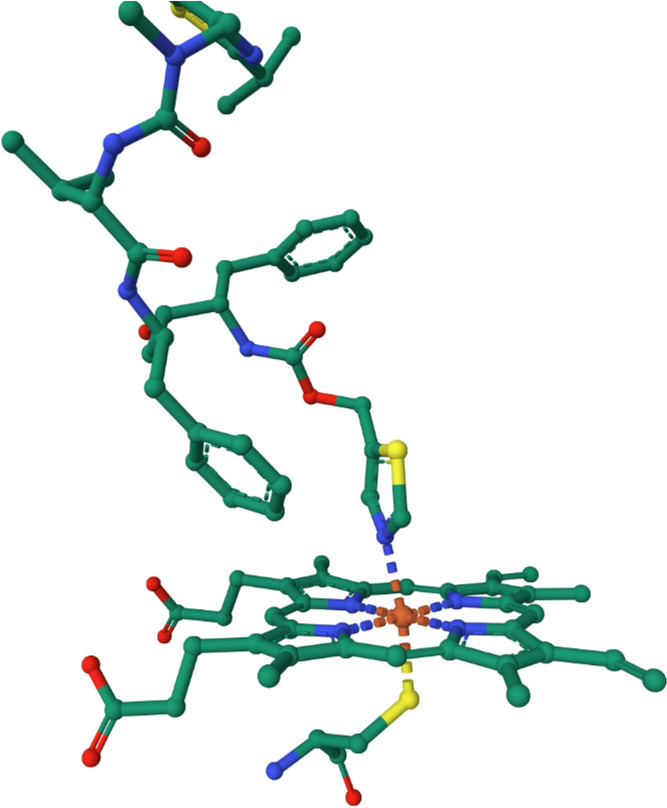
Active site of a P450 with Cys proximal ligand and with ritonavir coordinated in the distal position. (CYP3A5; PDB 5VEU [274])

The proximal axial ligand is the thiolate of a Cys residue and it plays an important role not only in the cleavage of the O–O bond, but also in ensuring the correct folding of the protein, in electron transfer, and in substrate binding [275]. The active site of human P450 3A5 (CYP3A5) with ritonavir coordinated to iron trans to Cys is shown in Fig. 32 (PDB 5VEU [274]).

**Fig. 33:**
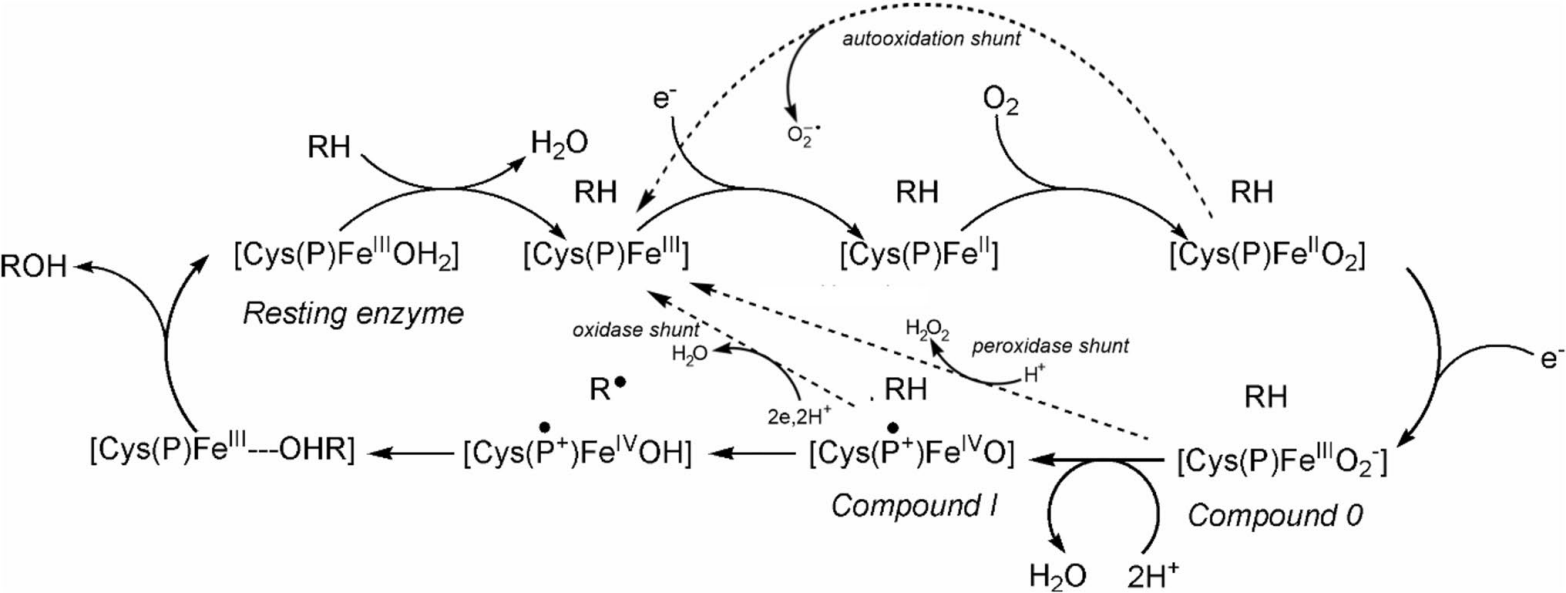
Generalised mechanism of the cytochromes P450

The generalized mechanism of the P450s is shown in Fig. 33. The details will depend on the enzyme involved and, in particular, the substrate RH [270].

The oxy-ferrous intermediate has been crystallised and its structure determined [276]. It is well-established that Compound 0 occurs in the cycle [277] and the presence of Compound I has been spectroscopically and kinetically demonstrated [278]. The entire catalytic cycle is remarkably choreographed by the protein on substrate binding, as has been demonstrated by molecular dynamics simulations [279, 280], and the interaction between the enzyme and the redox partner that provides the reducing equivalents (the [Fe_2_S_2_] protein putidaredoxin in the case of P450cam from *Pseudomonas putida*) is also important, priming the enzyme for its catalytic role [271]. A peroxide shunt uses peroxides to generate Compound I directly [281].

The reactive oxidant is Compound I, Fe^4+^=O with a porphyrin radical (see the earlier discussion of the oxo wall in Sect. "The mitochondrial electron transport chain"). A similar oxidant occurs in the peroxidase reaction cycle (see below). The key difference is that Compound I in the P450 cycle can abstract an H atom even from an unactivated C–H bond, as in R_1_R_2_R_3_CH → R_1_R_2_R_3_COH. This is because of the difference in the axial ligation of the P450 and the peroxidase enzymes: Cys in the first, His in the second. The much softer thiolate ligand increases the basicity of the ferryl O, overcoming the barrier of breaking a C–H bond [282–284]. This is the key feature of the P450 catalytic cycle.

There is a potential problem with producing such a reactive species as Compound I: if substrate oxidation occurs more slowly than Compound I is formed, Compound I could damage the enzyme. For example, the human liver enzyme CYP3A4 metabolises many therapeutic drugs but with fairly low efficiency [285]. The P450 enzymes have a redox chain comprising Tyr and Trp residues which guide the oxidising equivalents (i.e., “hole” migration) away from the active site to the enzyme’s surface to be scavenged by soluble reductants if substrate oxidation is slow [286, 287].

In addition to the cleavage of O_2_ and the binding of the substrate, the efficiency of the P450s depends on receiving electrons from the donor (usually NAD(P)H) through the participation of redox partners (RPs) [288]. The electron transfer chain is, therefore, given by the following equation:

25 $${\text{NAD}}\left( {\text{P}} \right){\text{H }} \to {\text{ RPs }} \to {\text{ haem }} \to {\text{ O}}_{{2}}$$

The rate of electron transfer (determined by the potentials of the redox centres and the distance between them [289]) is often the rate limiting step in enzyme turnover. Some P450s have their redox partner(s) fused into the same polypeptide, an obvious advantage to ensure efficient electron transfer; others are multicomponent systems that have to solicit their redox partners to affect their catalytic function. Another factor that controls the turnover rate is the efficiency with which the enzyme binds the substrate. For example, P450cam binds camphor very efficiently but styrene much more poorly [290].

**Fig. 34:**
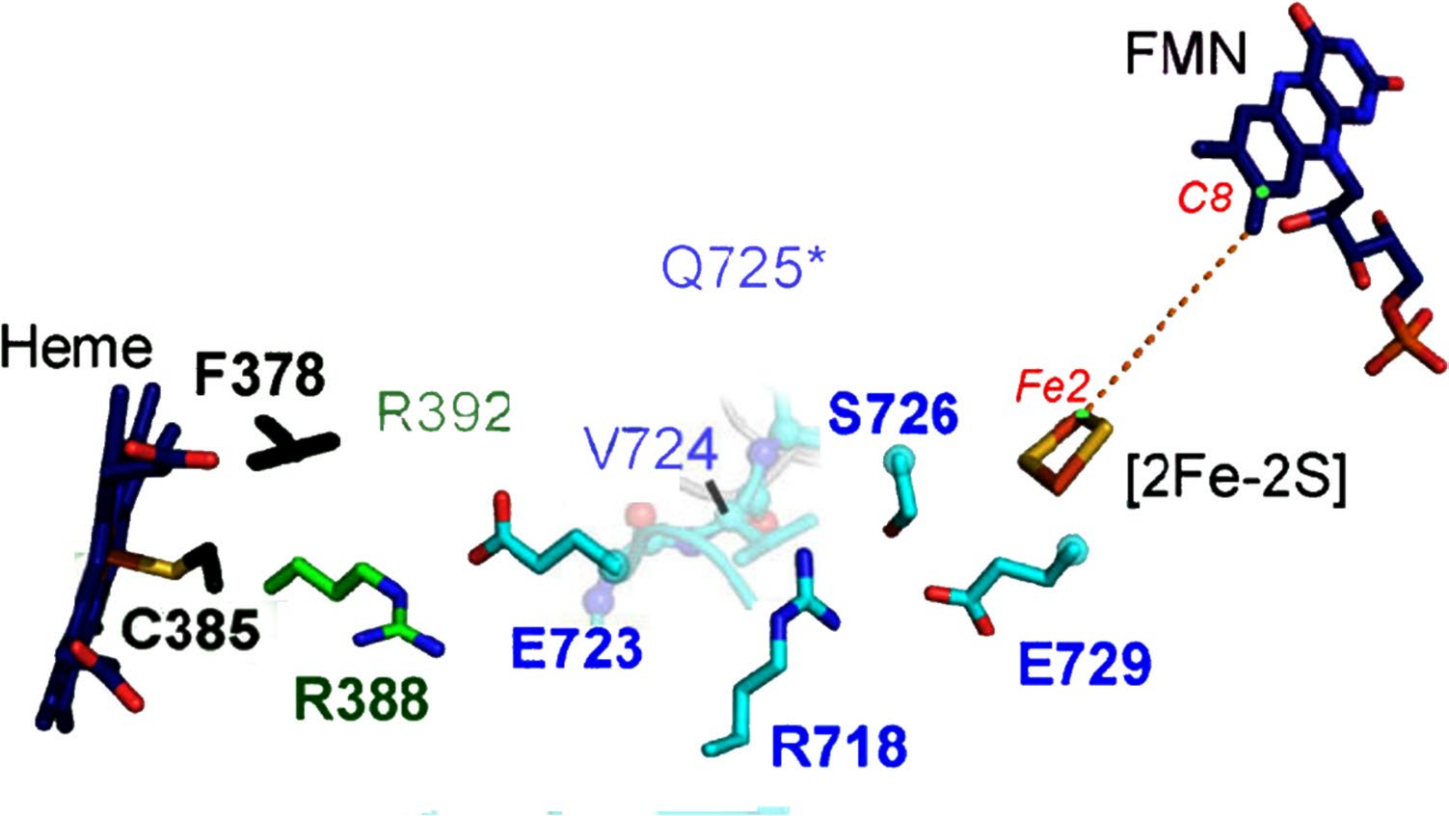
Probable electron transport chain NAD(P)H → FMN → [Fe_2_S_2_] → haem of the self-sufficient P450, CYP116B46, from *T. thermophiles.* Reproduced with modifications from [291]

As mentioned, the P450 family is very large, and P450 classes (Class I to Class X) are defined based on the redox partners used [288]. For example, Class I P450s prokaryotic enzymes are multicomponent enzymes that use a flavin adenine dinucleotide (FAD)-containing ferredoxin reductase (FdR) to oxidise NAD(P)H; the electrons are then transferred to [Fe_2_S_2_] ferredoxin protein (Fdx) which is the source of electrons for the haem group of P450. The [Fe_2_S_2_] centre shuttles between FdR and the P450 domain, binding 12–14 Å from the proximal face of the porphyrin. The Class VI enzymes also use a FdR as the NAD(P)H oxidant; the electrons are then transferred to a flavin mononucleotide (FMN)-containing flavodoxin (Fld) in a domain that is fused with the P450 domain. Class VII P450s are so-called self-sufficient P450 systems where the RP components are fused with the P450 domain to provide the electron transfer chain NAD(P)H → FAD → FMN → haem, with electrons thought to be transferred through a chain of amino acids residues [291] (Fig. 34).

### Non-haem *iron* enzymes

**Fig. 35:**
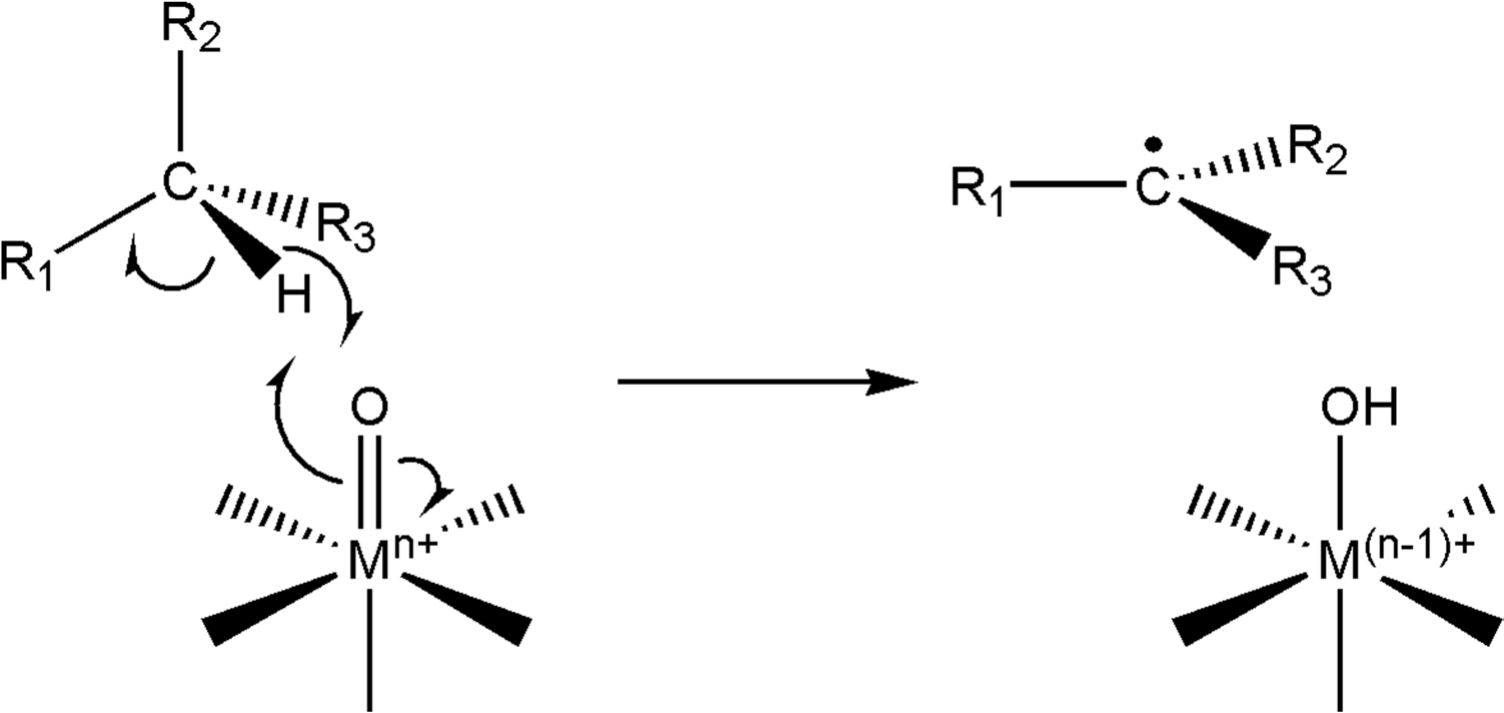
Homolytic cleavage of a C–H bond by a metal–oxo complex in which the metal is in a high oxidation state, conferring on the oxo ligand an electrophilic character

In addition to the P450 enzymes, iron, manganese or copper enzymes are also capable of functionalising C–H bonds [292, 293]. As mentioned in Sect. "The mitochondrial electron transport chain", metal–oxo (and metal hydroxo) complexes in which the metal is in a high oxidation state (for example, Fe^4+^=O) have electrophilic oxo (or hydroxo) ligands. There are various routes that lead to the formation of these intermediates [294]. One of the reactions they can perform is the homolytic cleavage of a C–H bond [293, 294] in a proton-coupled electron transfer, PCET (also called a concerted proton–electron transfer, CPET), with the electron and the proton of the C–H bond ending up on different atoms (Fig. 35). We have already seen (Sect. "The Cytochromes P450") how in the cytochromes P450, cleavage of the dioxygen bond leads to the formation of Compound I (P^•^Fe^4+^=O) and, after C–H bond cleavage, Compound II (Fe^4+^–OH).

The iron-dependent *α*-ketoglutarate dioxygenases use α-ketoglutarate as a cofactor that binds to a Fe^2+^ centre to catalyse the reaction with O_2_ as the terminal oxidant (Eq. 26); [295] and references therein). The enzymes are found in many living systems (over 1000 have been identified, including some 60 such oxygenases in humans).26

One of the key reactions catalysed by these enzymes is hydroxylation, but there are many others, including halogenations, epoxidations, ring expansion, and ring formation, desaturation, N-atom oxidation, and endoperoxidation [296–298].

**Fig. 36:**
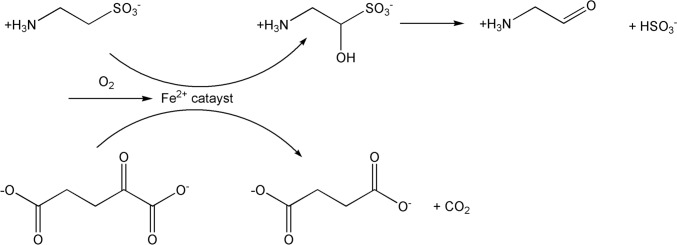
Reaction of taurine and α-ketoglutarate with O_2_ catalysed by taurine deoxygenase

Taurine dioxygenase is an example and catalyses the reaction shown in Fig. 36; taurine, R = NH_2_CH_2_CH_2_SO_3_^–^ in Eq. AAA, is a non-proteinogenic amino sulfonic acid found in animal tissues that plays several roles in humans, including the regulation of glucose, in lipid metabolism and in the development of skeletal muscle.

**Fig. 37:**
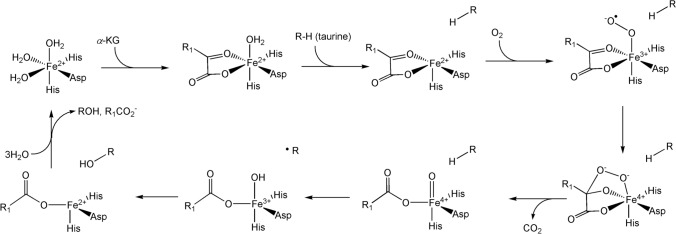
Likely reaction mechanism of taurine deoxygenase [293, 299, 300]

In the resting enzyme, Fe^2+^ is facially coordinated by two His residues and the carboxylate of Asp or Glu, and by three H_2_O molecules (Fig. 37). This is a common structural motif used by the non-haem iron enzymes for the activation of O_2_ [298]. The reaction begins with the binding of *α*-ketoglutarate to Fe^2+^, displacing two of the H_2_O ligands. Taurine (R–H) docks proximally to the active site and the third H_2_O ligand is displaced. O_2_ binds to Fe^2+^ in an end-on fashion and the non-coordinated O attacks the carboxylate of *α*-ketoglutarate leading to the formation of the Fe^4+^=O species. This then initiates the homolysis of a C–H bond of taurine producing a radical and Fe^3+^–OH. The transfer of OH to the taurine radical forms hydroxylated taurine. Release of this and succinate regenerates the resting enzyme [299]. (Hydroxylated taurine decomposes to aminoacetaldehyde and sulfite, Fig. 36.)

### Ribonucleotide Reductases (RNRs).

**Fig. 38:**
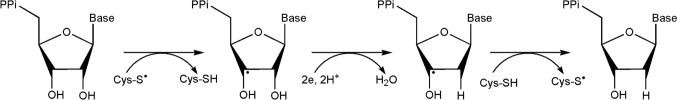
Reaction mechanism of a RNR. A cysteinyl radical initiates the reaction abstracting the 3’-H of the substrate (so the reaction is initiated by a one-electron oxidation). Ribose 2’-OH is released as water on a two-electron reduction. The return of the abstracted H, present on Cys, is returned to the 3’ position and the cysteinyl radical is regenerated

Ribonucleotide reductases catalyse the conversion of nucleotides to deoxynucleotides, precursors for DNA synthesis and repair [301, 302]. The reactions involve the generating of a cysteinyl radical by some route, and all RNRs basically follow the same reaction mechanism (Fig. 38) [303]. The capacity to produce the reactive Cys^•^ is stored elsewhere on the enzyme, away from the relatively open active site, and differs among the three classes of RNRs [304, 305].

The class I RNRs contain a bimetallic cluster that initiates radical production. They are made up of two units; the first, larger, unit (variously referred to as R_1_, NrdA or NrdI) is where the reduction of ribonucleotides take place; the second, smaller, unit (R_2_, NrdB or NrdF), is responsible for radical generation, a stable tyrosyl radical close to a binuclear metal centre that then generates the required Cys^•^. The class I RNRs are further subdivided into subclasses, depending on the identity of the bimetallic site: a 2Fe cluster (class Ia), a 2Mn cluster (Ib, Id), or a FeMn cluster (Ic) [306].

In the class Ia RNRs, the 2Fe^2+^ core reacts with O_2_ to produce O_2_^2–^ bridging two Fe^3+^ centres. Electron transfer and scission of the O–O bond yields bis-*μ*-oxo-Fe^3+^Fe^4+^ which is the oxidant that generates Tyr^•^ and 2Fe^3+^. Reduction by, for example, thioredoxin or glutaredoxin, which are themselves re-reduced by thioredoxin reducatse and NADPH, generates the active 2Fe^2+^ state [308, 309].

**Fig. 39:**
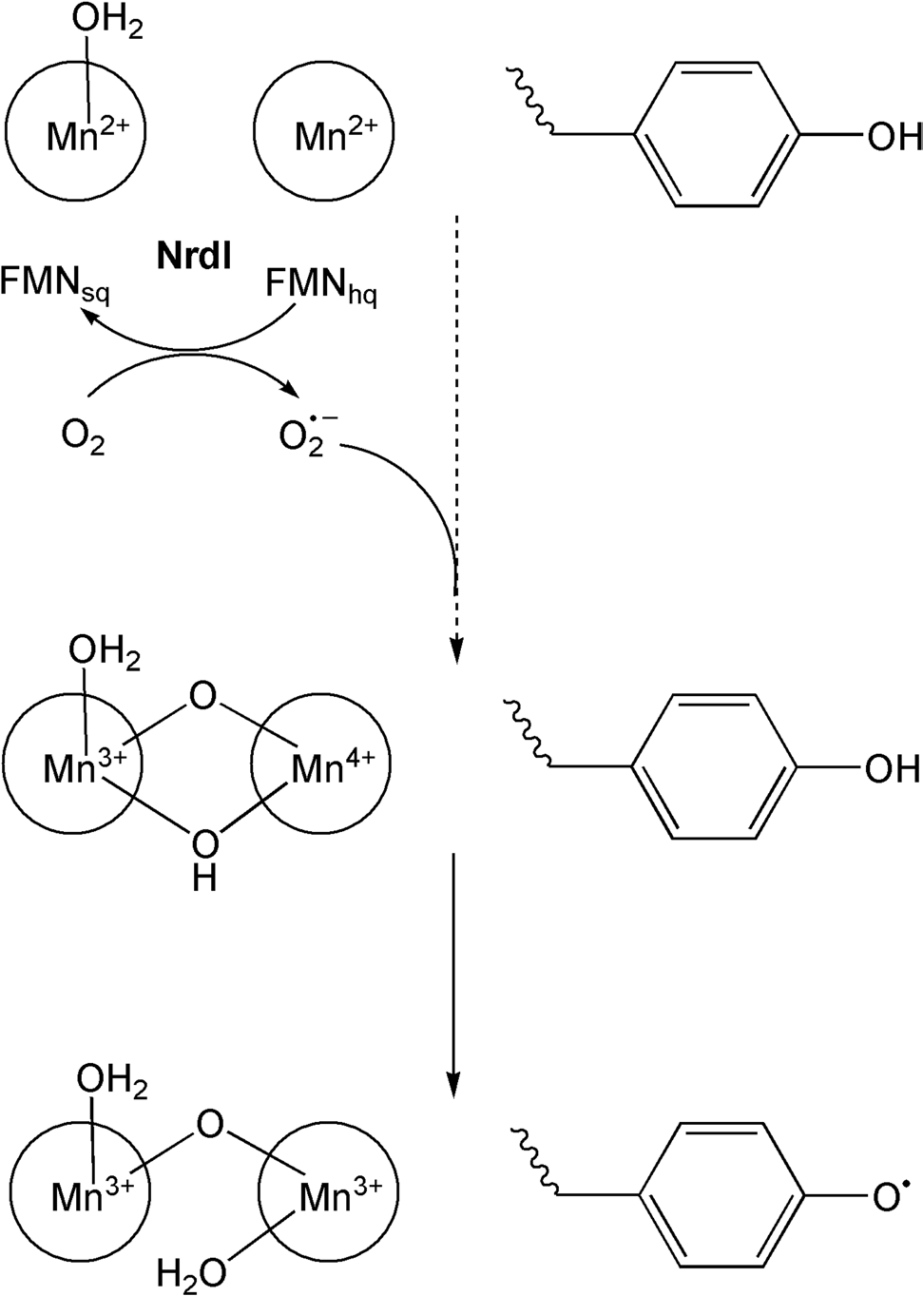
Tyrosyl radical is generated near a di-Mn centre in the class Ib RNRs. This initiates the formation of Cys^•^ (Fig. 38). Adapted from [307]

The generating of the tyrosyl radical near a di-manganese centre in the class Ib RNRs from the gram-positive bacterium *Bacillus subtilis* [310] is shown schematically in Fig. 39 as an example. Unlike the di-iron class RNRs, the di-manganese enzymes do not react with O_2_ but instead require the reduced form of a flavodoxin-type protein, NrdI_hq_ (hq = hydroquinone) which is oxidized to NrdI_sq_ (sq = semiquinone) and reduces O_2_ to O_2_^•–^. O_2_^•–^ in turn reacts with the bimetallic centre. Electron transfer and O–O bond cleavage produces a Mn^3+^–O–Mn^4+^ intermediate that oxidises Tyr to Tyr^•^.

There are many variations. For example, the class Ic RNR from the bacterium *Chlamydia trachomatis* has a Phe in place of Tyr near the bimetallic site [311]. It generates Cys^•^ by means of a Mn and Fe bimetallic site [312], with the oxidized Mn^4+^/Fe^3+^ capable of directly oxidising Cys to produce Cys^•^ [312]. The class Id RNRs from, for example, the bacteria *Facklamia ignava* and *Leeuwenhoekiella blandensis*, use a similar strategy, but with a Mn^3+^/Mn^4+^ cluster to directly generate Cys^•^ [313]. The class Id RNR’s also do not react directly with O_2_, but instead with one equivalent of O_2_^•–^ or two equivalents of H_2_O_2_ in addition to a one electron reduction.

### Multi-copper oxidases

Multicopper oxidases (MCOs) oxidise a wide variety of substrates while (usually) reducing O_2_ to H_2_O [182, 314–316]. Examples include laccase (found in fungi and plants, and which oxidises diamines and phenols), ascorbate oxidase (found in higher plants, which catalyses the oxidation of ascorbic acid to L-dehydroascorbic acid) and nitrite reductase (a bacterial and fungal enzyme, but one that reduces NO_2_^–^ to NO and H_2_O, rather than reducing O_2_).

**Fig. 40:**
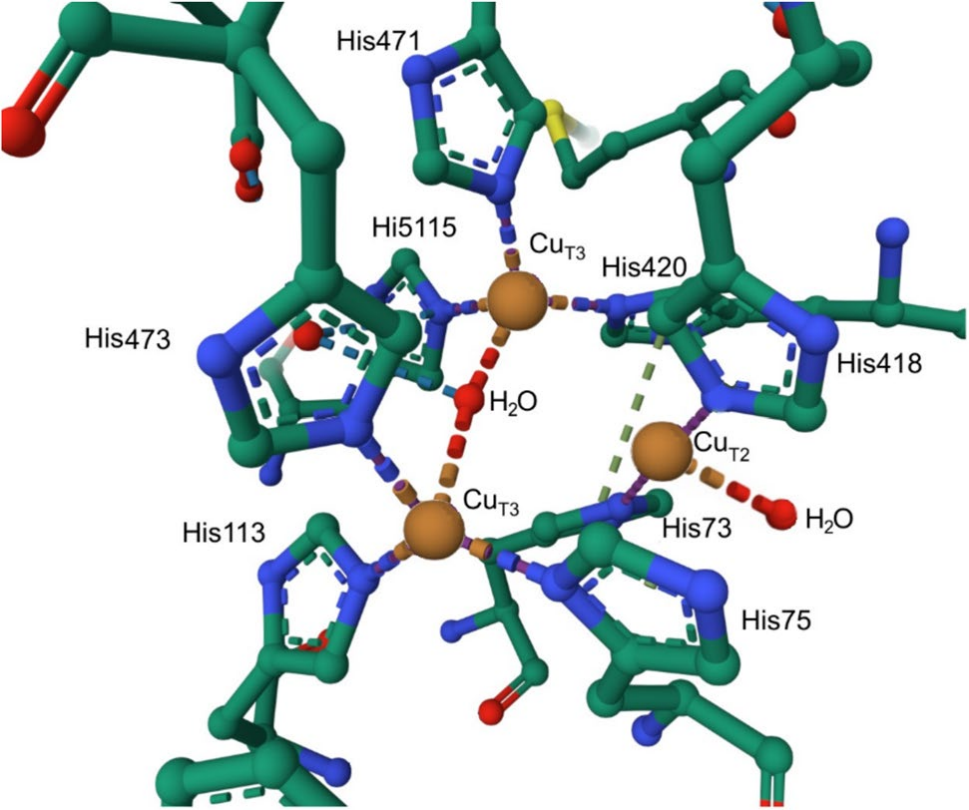
Trinuclear copper site of the multicopper oxidase CueO from *E. coli* (PDB 1KV7 [123]). In the oxidised state of the enzyme all copper ions are in the + 2 oxidation state. Initiation of the catalytic cycle requires the uptake of three electrons, one at a time, from the mononuclear copper site to reduce all Cu^2+^ to Cu^+^. The H_2_O bridging the two Cu_T3_ ions is released in the process. (Also see Fig. 41)

Typically, electrons are accepted from the substrate at a mononuclear copper site, [Cu(His_2_CysMet)], see Sect. "The cytochrome b6f complex – and a note on the blue copper proteins", and pass through a His–Cys–His pathway [317] to a tri-copper site some 13 Å from it where reduction of O_2_ (or N_2_O) occurs. The trinuclear cluster of the multicopper oxidase CueO from *E. coli* is shown in Fig. 40 [123]. The enzyme is involved in copper homeostasis [318]. The tri-copper site contains two Cu ions coordinated to three His ligands and solvent H_2_O (or OH^–^) (termed Cu_T3_) and a Cu ion coordinated to two His residues and a solvent H_2_O (or OH^–^), termed Cu_T2_.

**Fig. 41:**
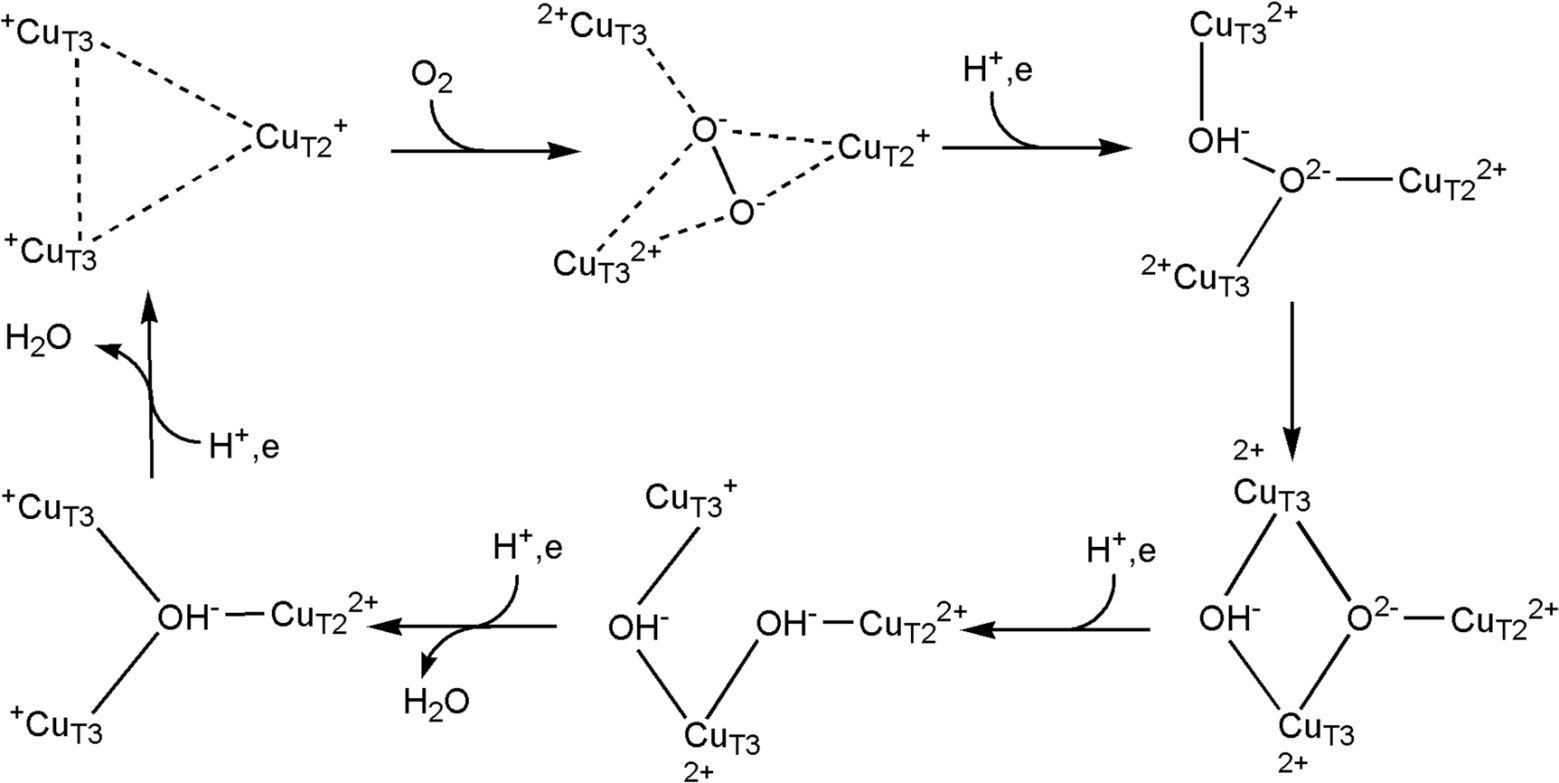
Possible catalytic cycle for the MCOs. In the reduced state of the enzyme all copper ions are in the + 1 oxidation state. This is the form on the enzyme that binds O_2_. The first step (*k* ≈ 10^6^ M^–1^ s^–1^) is the formation of a peroxide intermediate, O_2_^2–^, with two of the copper ions oxidised to the + 2 state [321]. Bound peroxide interacts with all copper ions [322]. The uptake of the first electron results in cleavage of the O–O bond of peroxide with a barrier of a modest 60–65 kJ mol^–1^ [314, 320, 323], the largest energy barrier of the entire cycle [320]. The successive uptake of 3 further electrons and four protons, and release of 2 H_2_O, regenerates the reduced form of the enzyme and completes the catalytic cycle

Based on extensive computational studies and experimental evidence, a reaction mechanism for the reduction of O_2_ to H_2_O by the multicopper oxidases, shown in Fig. 41, has been proposed [319, 320]. The presence the three Cu ions interacting with O_2_ ensures the ability of the enzyme to, initially, effect the two-electron reduction of O_2_ to O_2_^2–^, overcoming the spin-forbidden reduction of *S* = 1 O_2_ by an organic substrate (*S* = 0); this is then followed by four one-electron transfers coupled to proton uptake and the release of two H_2_O molecules.

### The Catalases, Peroxidases, Superoxide Dismutases and Superoxide Reductases

The terminal step of the mitochondrial electron transport chain (see Part 2) sees the reduction of O_2_ to H_2_O with electrons derived from NADH. However, as we have seen, the partial reduction of O_2_ leads to a series of ROS which must be defended against (Eq. 27):

27 $${\text{O}}_{2} \overset{\text{e}} \longrightarrow 
 {\text{O}}_{2}^{ \bullet - } \overset{\text{e}} \longrightarrow  {\text{H}}_{2} {\text{O}}_{2} \overset{\text{e}} \longrightarrow  {\text{OH}}^{ \bullet } \overset{\text{e}} \longrightarrow  {\text{H}}_{2} {\text{O}}$$

The oxidoreductases such as the catalases, peroxidases, superoxide dismutases and superoxide reductases serve important functions in providing such a defence [324, 325]. There are many other enzymes (for example, glutathione peroxidases [326], lignin peroxidase [327] and the haloperoxidases [328]) that are important in defence against ROS. Knowledge about catalase goes back to 1900 [329] and bovine liver catalase was crystallised in 1937 [330]. Current interest in the peroxidases includes their use as bioremediation catalysts [331, 332].

Low levels of ROS are desirable as they are involved in the regulation of a variety of biochemical transformations and the modulation of signal transduction pathways [54, 333–336]. As already mentioned, increased levels of ROS are harmful, leading to a condition termed oxidative stress that causes, *inter alia*, DNA base oxidation, lipid peroxidation and protein decarbonylation [337]. Oxidative stress has been implicated in many conditions including cancer, cardiovascular diseases, neurological disorders, psychiatric diseases and ageing [54, 338]. ROS may arise from mitochondrial processes [339], NAD(P)H oxidases, or as a consequence of external factors such as exposure to radiation or pollution.

Attention is often focussed on species such as H_2_O_2_, ^1^O_2_, O_2_^•–^ and OH^•^, but nitrogen, carbon, sulfur, selenium and halogen radicals are also important sources of oxidative stress [333, 336, 340–343] (also see Sect. "Reactive Oxygen (and Other) Species"). In addition to these defensive enzymes, antioxidants such as vitamin C, vitamin E and carotenoids, are important in controlling ROS levels.

Superoxide can be formed by the autoxidation of, for example, Fe.^2+^–O_2_ haem complexes (see Fig. 33 for an example). Another important source arises as a consequence of radical repair by thiols [344] (Eqs. 28–30):

28 $${\text{R}}^{ \prime \bullet } + {\text{ RSH }} \to {\text{ R}}^{\prime} {\text{H }} + {\text{ RS}}^{ \bullet }$$

29 $${\text{RS}}^{ \bullet } + {\text{ RS}}^{-} \to {\text{ RSSR}}^{{ \bullet {-}}}$$

30 $${\text{RSSR}}^{{ \bullet {-}}} + {\text{ O}}_{{2}} \to {\text{ RSSR }} + {\text{ O}}_{{2}}^{{ \bullet {-}}}$$

H_2_O_2_ can arise from the two electron reduction of O_2_ by oxidases such as xanthine oxidase [345] and glucose oxidase [346] and from the dismutation of superoxide catalysed by superoxide dismutase [347] (Eq. 31):

31 $${\text{2O}}_{{2}}^{{ \bullet {-}}} + {\text{ 2H}}^{ + } \to {\text{ H}}_{{2}} {\text{O}}_{{2}} + {\text{ O}}_{{2}}$$

**Fig. 42:**
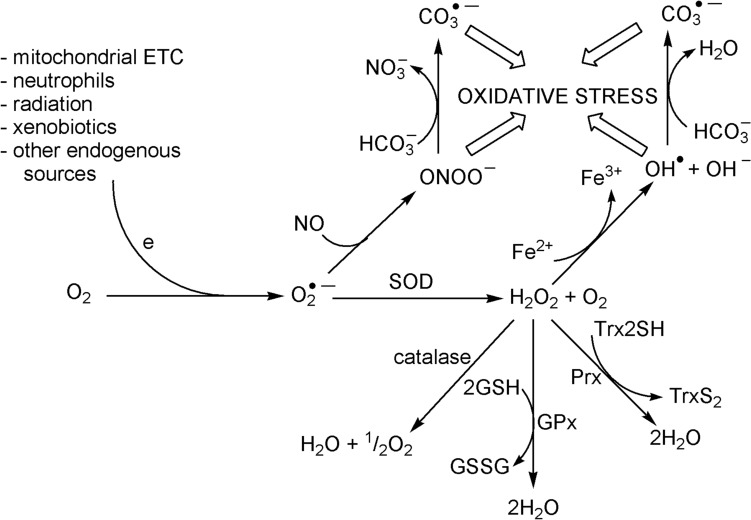
Formation of O_2_^•–^, its consequences, and its control. SOD, superoxide dismutase; GPx, glutathione peroxidase; GSH, glutathione; GSSH, glutathione disulfide; Prx, thioredoxin (Trx)-dependent peroxiredoxin; TrxSH_2_, reduced thioredoxin; TrxS_2_, oxidised thioredoxin. (Thioredoxin is a small redox protein; SH, Cys)

Superoxide is fairly unreactive towards amino acids (except for Cys and Met) [348] and it reacts slowly with biomolecules such as DNA, as does H_2_O_2_ [349]. Superoxide does target proteins containing iron–sulfur clusters such as dehydratases and hydrolases [350, 351]. Control of superoxide and H_2_O_2_ concentration in vivo is important to minimise the formation of OH^•^ (see Sect. "Metalloproteins in electron transfer chains") which rapidly and indiscriminately reacts with many biomolecules, including DNA, membranes and proteins [352]. As already discussed, Sect. "Reactive Oxygen (and Other) Species", also important are the formation of the carbonate radical CO_3_^•–^ and peroxynitrite, ONOO^–^. A simplified view of the electron transfer pathway that generates O_2_^•–^, and its control, is shown in Fig. 42.

O_2_^•–^/HO_2_^•^ (p*K*_a_ = 4.8) undergoes spontaneous disproportionation in a pH-dependent manner (*k*_max_ = 2 × 10^7^ M^–1^ s^–1^, pH 4.8; *k* < 10^6^ M^–1^ s^–1^ at pH 7 [353]), but this is orders of magnitude slower than the disproportionation reaction catalysed by Cu,Zn-SOD (2 × 10^9^ M^–1^ s^–1^, pH 7), or Mn-SOD and Ni-SOD (*k* ≈ 4 × 10^9^ M^–1^ s^–1^, pH 7).

Superoxide dismutases are metalloproteins. The SOD1 enzymes are Cu,Zn enzymes found in the cytosol and the mitochondrial intermembrane space. SOD2 is a Mn enzyme found in the matrix and inner membrane of mitochondria. SOD3 is a Cu,Zn enzyme found in the extracellular compartment. Other known SODs include Fe-SOD, found in prokaryotes such as *E. coli*, some eukaryotes such as *Vignia unguiculata*, in chloroplasts, and in anaerobes [354, 355]. Ni-SOD is found predominantly in prokaryotes and cyanobacteria [356].

The basic mechanism of all SODs is given by Eqs. 31 and 32, the first an oxidation of superoxide, and the second its reduction:

32 $${\text{M}}^{{\left( {{\text{n}} + {1}} \right) + }} + {\text{ O}}_{{2}}^{{ \bullet {-}}} \to {\text{ M}}^{{{\text{n}} + }} +_{{\phantom{a}}} {\text{O}}_{{2}}$$

33 $${\text{M}}^{{{\text{n}} + }} + {\text{ O}}_{{2}}^{{ \bullet {-}}} + {\text{ 2H}}^{ + } \to {\text{ M}}^{{\left( {{\text{n}} + {1}} \right) + }} + {\text{ H}}_{{2}} {\text{O}}_{{2}}$$

Thus, Fe-SOD cycles between Fe^3+^ and Fe^2+^; Mn-SOD between Mn^3+^ and Mn^2+^; and Ni-SOD between Ni^3+^ and Ni^2+^.

**Fig. 43:**
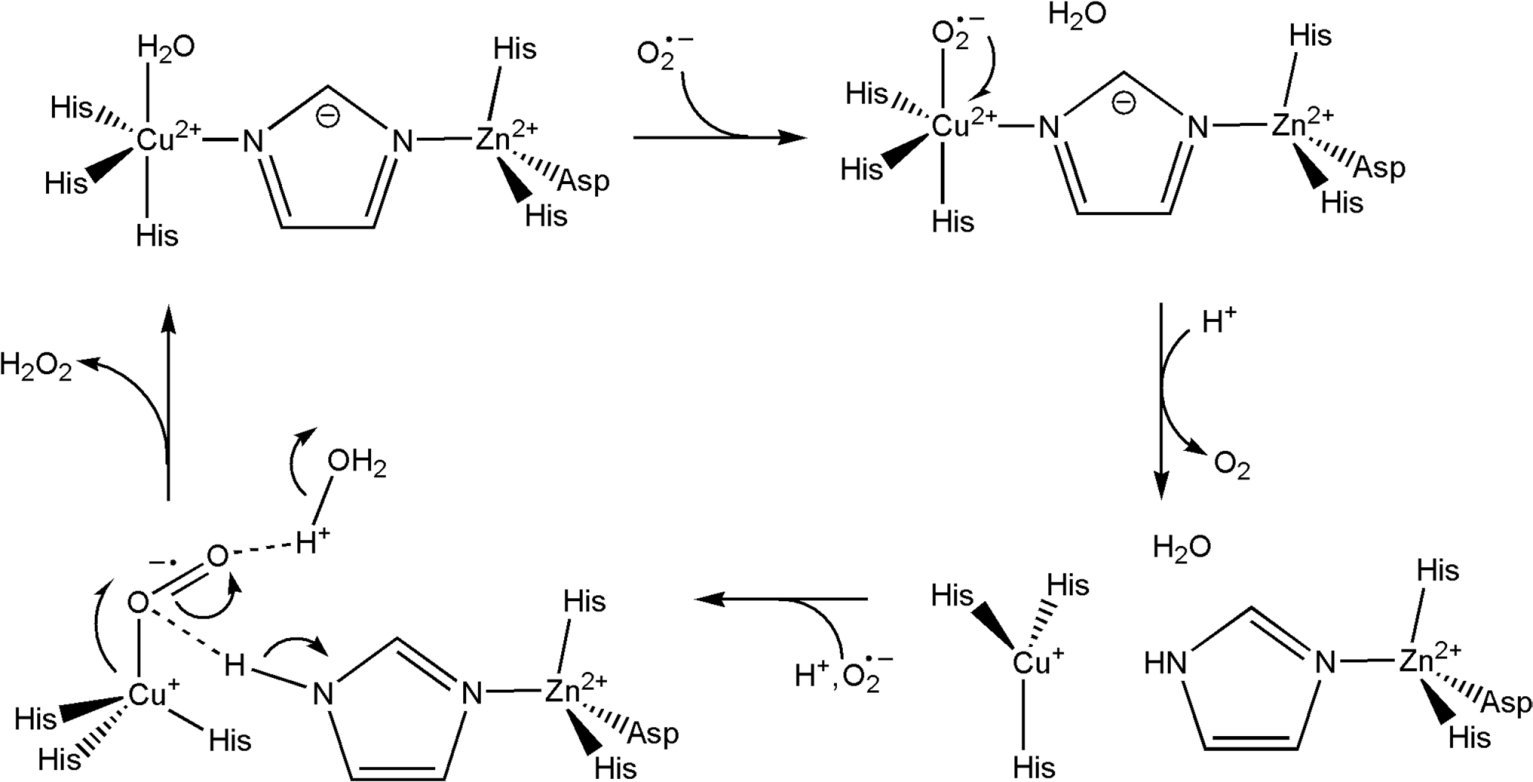
Dismutation of superoxide by Cu,Zn-SOD

In the resting state of Cu,Zn-SOD, His^–^ bridges Cu^2+^ and Zn^2+^. When O_2_^•–^ is coordinated by Cu^2+^, reducing it to Cu^+^ both H_2_O and the bridging His are lost, producing a three-coordinate Cu^+^ complex which acts as the reductant of a second superoxide ion to produce H_2_O_2_ (Fig. 43 [357, 358]). The reaction is very fast (*k* = 2 × 10^9^ M^–1^ s^–1^, pH 7).

In many SOD enzymes, the substrate is guided to the binding site by electric field vectors set up by conserved amino acid residues [359, 360], largely negatively charged near the enzyme surface at the entrance to a channel that leads to the active site, and positively charged near the active site, augmenting the positive charge on the metal.

Other enzymes capable of removing superoxide are the superoxide reductases, SORs, [361, 362] which reduce (rather than dismutate) O_2_^•–^ to H_2_O_2_ (Eq. 34):

34 $${\text{M}}^{{\left( {{\text{n}} - {1}} \right) + }} + {\text{ O}}_{{2}}^{{ \bullet {-}}} + {\text{ 2H}}^{ + } \to {\text{ M}}^{{{\text{n}} + }} + {\text{ H}}_{{2}} {\text{O}}_{{2}}$$

**Fig. 44:**
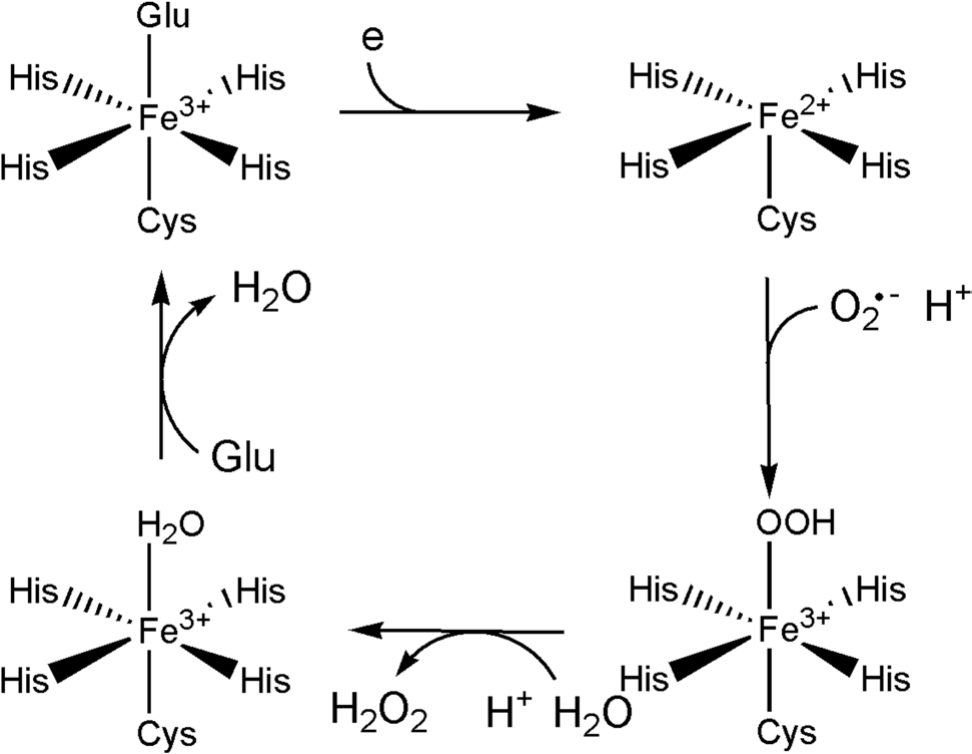
Reaction catalysed by centre II of the SORs [361]

There are two types of these enzymes, one containing a one-iron centre and the other two one-iron centres. The iron centre common to both SORs is termed centre II. In the ferric form, the iron is octahedrally coordinated by four His residues in the equatorial plane, and axially coordinated by Cys and Glu. In the ferrous form, the metal is five-coordinate, Glu having been displaced. In centre I, Fe is coordinated by four Cys residues. Iron is high spin in both the oxidised (*S* = ^5^/_2_) and reduced (*S* = 2) forms. The mechanism of the reaction is outlined in Fig. 44. The electron donor is often a reduced rubredoxin, in its turn reduced by a NAD(P)H-dependent oxidoreductase, but other cellular reductants are clearly operative in organisms that lack a gene for coding a rubredoxin.

**Fig. 45:**
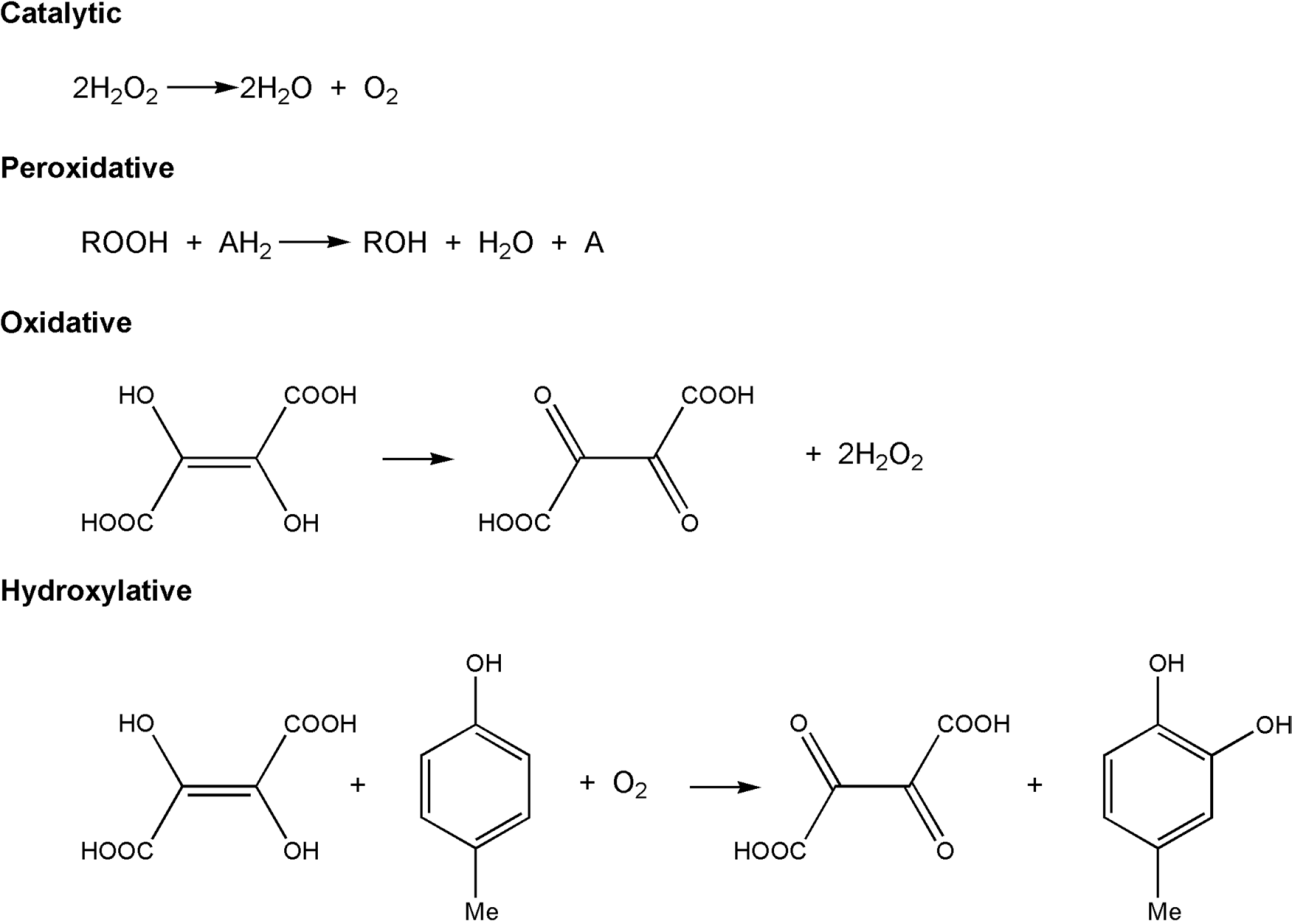
Reactions catalysed by the peroxidases

Key enzymes in the protection against ROS are the catalases and peroxidases. The peroxidases are widespread in nature, with four haem peroxidase superfamilies that arose independently in evolution. They differ in overall fold, active site architecture and enzymatic activity [363–365]. Typical reducing substrates, AH_2_, in the peroxidative reactions include aromatic phenols, phenolic acids, indoles, amines and sulfonates [366]. They decompose peroxides, including H_2_O_2_, oxidising organic or inorganic substrates in the process, and play an important role in mammalian biochemistry [367]. The reactions catalysed by the peroxidases have been classed as catalytic, peroxidative, oxidative and hydroxylative (Fig. 45) [368–370]. The oxidative properties of the peroxidases have been exploited in many fields, including medical, technological and industrial applications [371].

In addition to their role in converting H_2_O_2_ to O_2_ (Eq. 34), catalases are involved in a wide range of functions [372]. They occur in virtually all eukaryotes and are found within peroxisomes, in the mitochondria and in the nucleus [373]. The exceptional catalytic efficiency of the catalases has been utilised for the degradation of environmental pollutants [374]. Many catalases have a haem group as the active site. Non-haem catalases are found in bacteria; they contain two Mn ions at the active site [375]:

35 $${\text{2H}}_{{2}} {\text{O}}_{{2}} \to {\text{ 2H}}_{{2}} {\text{O }} + {\text{ O}}_{{2}}$$

The catalytic cycle involves the initial coordination of H_2_O_2_ and reduction of Mn^3+^ to Mn^2+^ with release of O_2_ (Eq. 35) followed by oxidation of Mn^2+^ to Mn^3+^ by a second molecule of H_2_O_2_ (Eq. 36):

36 $${\text{Mn}}^{{{3} + }} ,{\text{Mn}}^{{{3} + }} + {\text{ H}}_{{2}} {\text{O}}_{{2}} \to {\text{ Mn}}^{{{2} + }} ,{\text{Mn}}^{{{2} + }} + {\text{ 2H}}^{ + } + {\text{ O}}_{{2}}$$

37 $${\text{Mn}}^{{{2} + }} ,{\text{Mn}}^{{{2} + }} + {\text{ H}}_{{2}} {\text{O}}_{{2}} + {\text{ 2H}}^{ + } \to {\text{ Mn}}^{{{3} + }} ,{\text{Mn}}^{{{3} + }} + {\text{ 2H}}_{{2}} {\text{O}}$$

The kinetics are much slower than for the haem-containing catalases (typical *k*_cat_ of the order of 10^5^ and 10^7^ M^–1^ s^–1^, respectively [376, 377]). A possible mechanism for the reaction has been proposed [378].

**Fig. 46:**
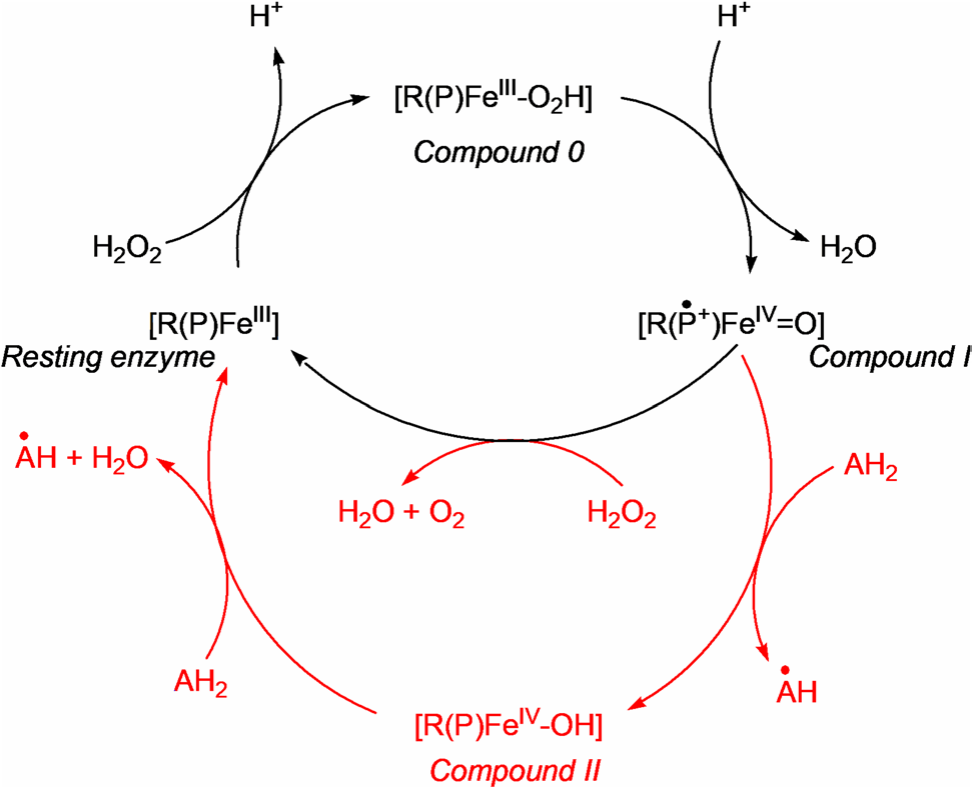
Generalised scheme for the reactions catalysed by the haem catalases (black) and peroxidases (red). The proximal axial ligand, R, is Tyr in beef liver catalase and His in horseradish peroxidase. (P) = porphyrin. Reaction of Compound I of catalase with the donor of a single electron will produce the hydroxyferryl Compound II [390]. Catalase trapped as Compound II slowly reverts back to the resting enzyme [391]. Catalase can act as a peroxidase, albeit relatively slowly, for example, in the reaction of ethanol with compound I to produce acetaldehyde [392]

A schematic of the reactions catalysed by the haem catalases and peroxidases in shown in Fig. 46 The axial ligand in the catalases is Tyr; in the peroxidases it is His, itself hydrogen bonded to an Asp residue which confers on His a significant imidazolate character. The anionic character of the axial ligands is important in stabilising the (formally) Fe^4+^ character of Compound I and Compound II.

Compound 0 is a transient intermediate. In the presence of excess peroxide, compound III, [R(P)Fe^III^O_2_^•–^] is formed [366, 379]. If the reductant AH_2_ is absent, then the peroxidases are capable (not very efficiently) of converting H_2_O_2_ into H_2_O and O_2_ [380]. Under conditions of low concentrations of H_2_O_2_, catalases use an alternative reductant, usually NADPH, or a Tyr residue, to escape entrapment as Compound I [243, 381–384]. (Catalase has a high affinity for binding NADPH [385].) Both Compound I and Compound II are powerful oxidants with *E*^o^ ≈ 1 V.

**Fig. 47:**
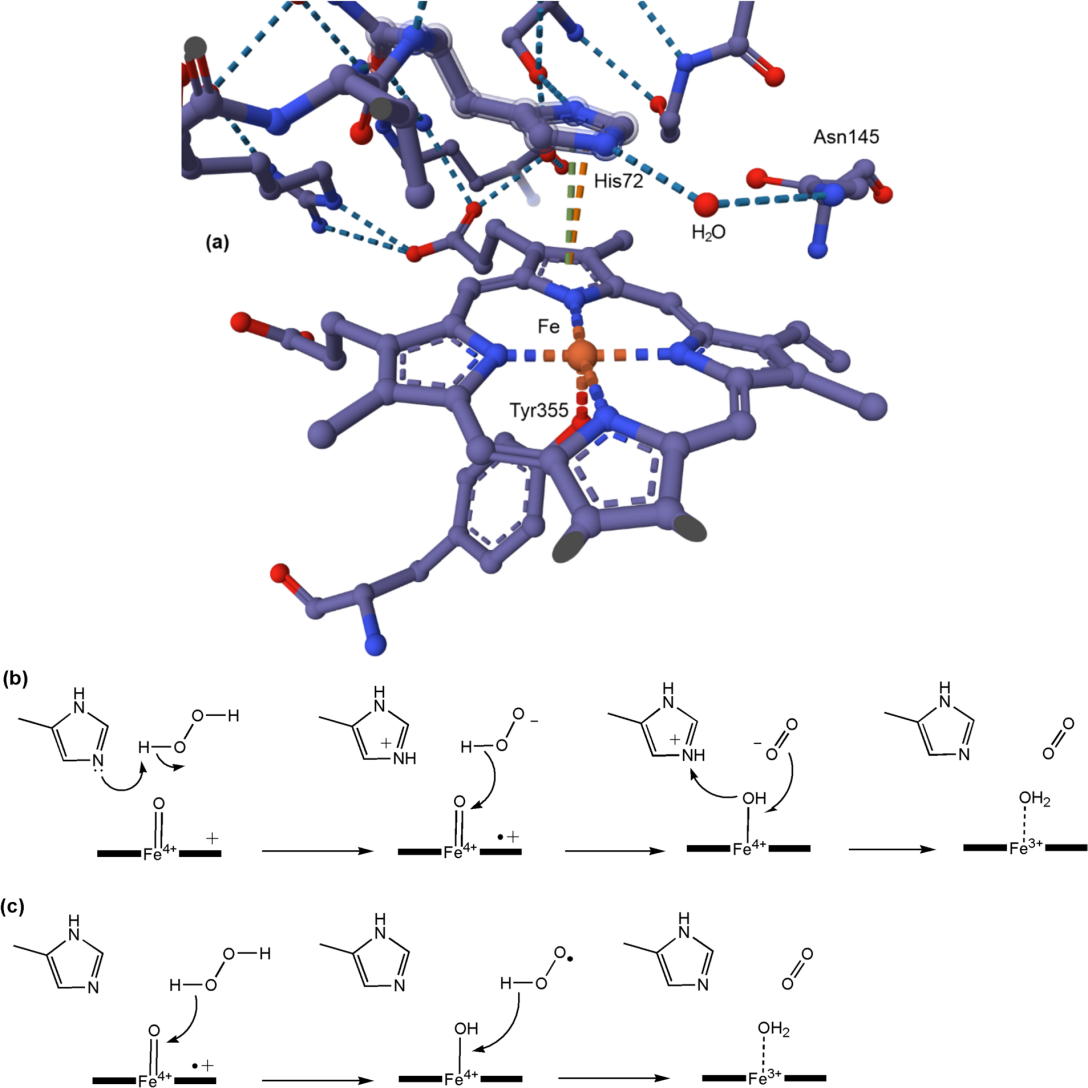
**a** Active site of human erythrocyte catalase (PDB code 1DGB) showing the distal His residue (here, His72) [384]. **b**, **c** Two feasible mechanisms for the reaction of Compound I with H_2_O_2_ in the second step of the catalase reaction cycle [387, 388]

In the catalase reaction, both oxygen atoms in the O_2_ product come from the same H_2_O_2_ substrate [386]. Catalases contain a distal His residue (Fig. 47a). This serves as an acid–base catalyst and the two hydrogens of H_2_O_2_ are sequentially transferred to the oxyferryl unit of Compound I [387] (Fig. 47b). A direct mechanism (Fig. 47c) is also possible [388]. This will come into operation if the distal His residue is mutated out. In either case, the reaction involves in effect the intermediate formation of a Compound II-like species.

**Fig. 48:**
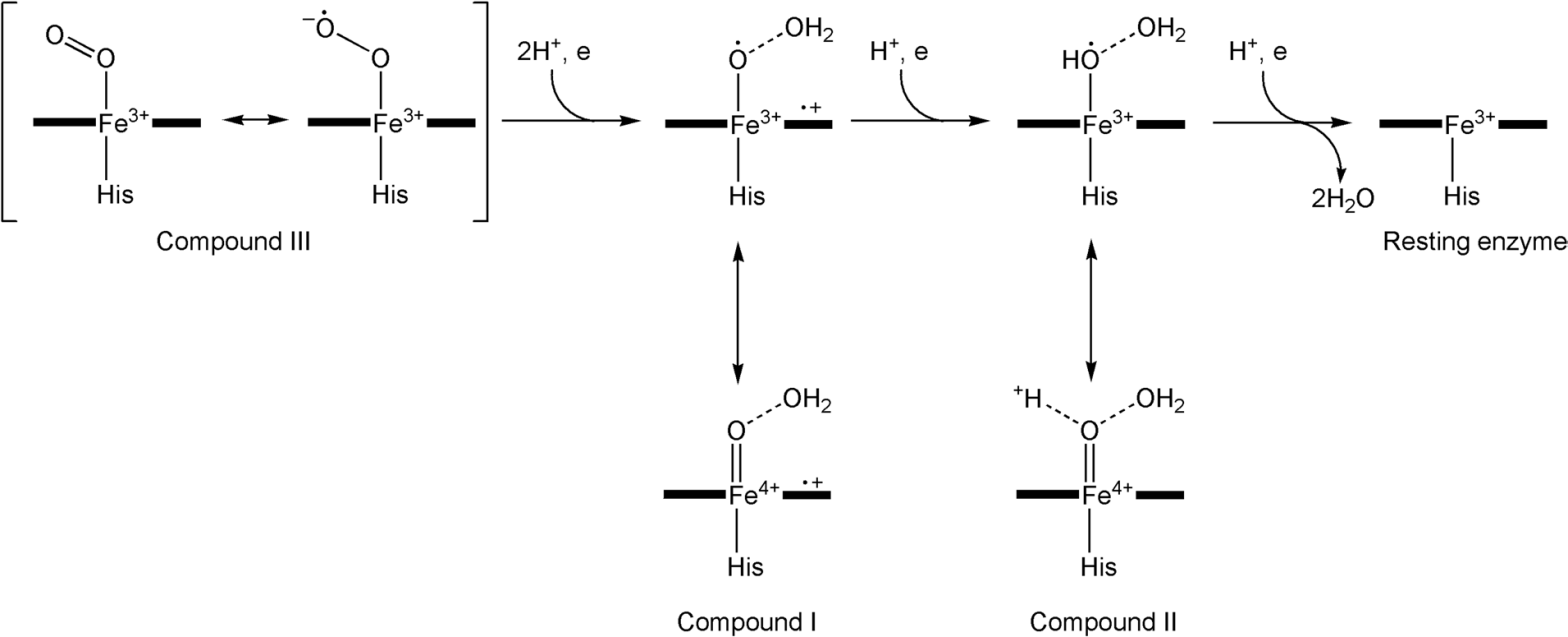
Reduction of Compound III of the peroxidase to form two equivalents of H_2_O (adapted from [389].)

A detailed description of the reduction of bound O_2_ in horseradish peroxidase obtained by X-ray diffraction analysis, with electrons liberated during irradiation, has been proposed [389]. Compound III was shown to have a very similar structure to oxyhaemoglobin or oxymyoglobin, probably best described as a hybrid of Fe^II^O_2_ and Fe^III^O_2_^•–^ with significant character of the latter. The probable reaction sequence [389], which produces two equivalents of H_2_O, is shown in Fig. 48.

## Extracellular *electron* transport

Organisms such as some bacteria use extracellular electron transport (EET) to generate energy for their growth and maintenance [3]. Electrons generated through the tricarboxylic acid cycle in the cytoplasm are transported through the cellular membrane to an electron acceptor, or imported from an electron donor through the cellular membrane to the cytoplasm [393]. Fe^2+^ can be used as a source of electrons, and Fe^3+^ as the electron acceptor, allowing microbial communities to perform cyclical iron oxidation and reduction in subsurface environments [394]. EET also occurs in the mammalian gut [395].

There is considerable interest in EET as it forms the basis of sustainable biotechnological systems such as microbial electrosynthesis cells [396, 397]. The underlying mechanism is the transfer of electrons, produced by cellular metabolism, along an electron transport chain usually using multi-haem cytochromes, through the cell surface to an electrode [398]. The alternative route, from electrode to the cell is also possible. In nature, the organisms accept electrons from donors or deliver electrons to acceptors that do not permeate the cell boundary [399]. Examples are the bacteria of the genera *Shewanellacea* and *Geobacteracea* that reduce a wide range of extracellular substrates, including Fe^3+^ and Mn^4+^ minerals, oxidising organic compounds to CO_2_ (in its simplest form, Eq. 37) [400]:

38 $${\text{4Fe}}\left( {\text{O}} \right){\text{OH }} + \, \left[ {{\text{CH}}_{{2}} {\text{O}}} \right] \, + {\text{ 8H}}^{ + } \to {\text{ 4Fe}}^{{{2} + }} + {\text{ CO}}_{{2}} + {\text{ 7H}}_{{2}} {\text{O}} \Delta G^{{\text{o}}} < \, 0$$

Fe^2+^ oxidising microorganisms couple the oxidation of Fe^2+^ to the reduction of O_2_, CO_2_ and NO_3_^–^ [401].

**Fig. 49:**
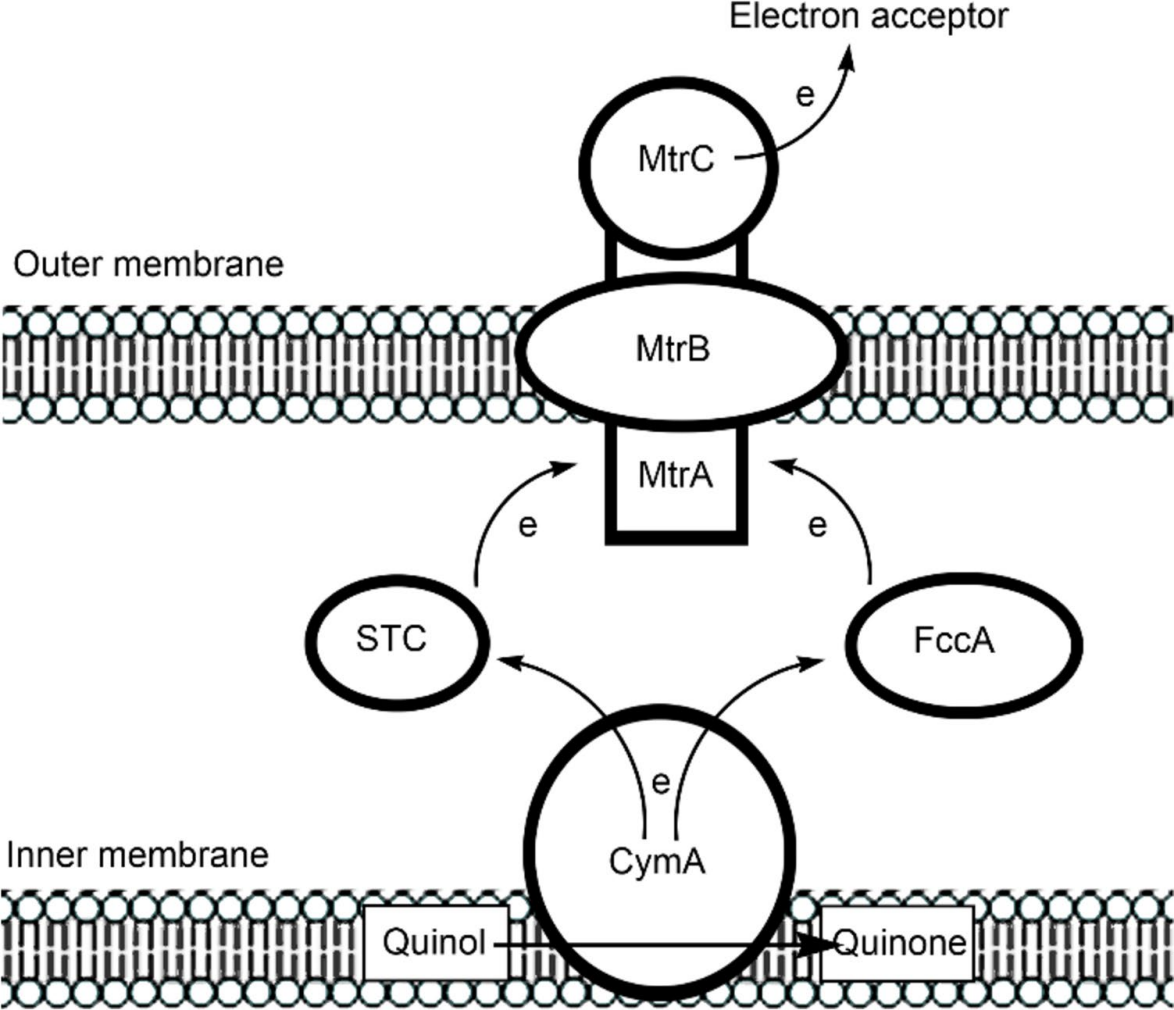
Outline of the electron transport chain of iron-reducing bacteria (see text for abbreviations)

The challenges that these organism face are [402] (i) the transfer of electrons from the quinol pool in the cytoplasmic membrane to electron carriers in the periplasm; (ii) the transfer of electrons across the outer membrane; and (iii) the transfer of electrons from cytochromes on the surface of the cell to the extracellular electron acceptors (Fig. 49). The quinol dehydrogenase CymA is a *c-*type tetrahaem protein [403]. It oxidises reduced quinols such as metaquinone-7 and transfers electrons to periplasmic cytochromes, including a small tetrahaem cytochrome (STC). (In *Shewanella oneidensis* strain MR1, for example, STC contains 91 residues and 4 haem binding sites [404].) Alternatively, electrons are transferred to fumarate reductase (FccA) [405, 406]. Electrons are transported by STC and FccA to a complex found in the outer membrane. The marine bacterium *Shewanella* uses a 20-haem protein complex MtrCAB as its outer membrane complex whereas *Geobacter*, found in anaerobic soils and aquatic sediments, uses a 6-haem protein, OmcS (see [407] and references therein). OmcS in an order of magnitude more efficient at electron transfer than MtrCAB because of larger haem–haem couplings and better insulation of the haem centres from the solvent [407]. MtrCAB consists of three complexes (MtrA, MtB and MtrC). While the MtrAB complex can transfer electrons to soluble oxidants on the cell surface, MtrC is required to transfer electrons to solid phase electron acceptors such as iron oxides [408]. In addition to MtrC, species within the genus *Shewanellacea* have other multi-haem cytochromes that serve as the reducing site for external electron acceptors [402]. In addition, *Shewanella* secretes flavins into the extracellular environment. They may serve as redox shuttles between surface cellular cytochromes and electron acceptors located too distant for direct electron transfer [409, 410].

**Fig. 50:**
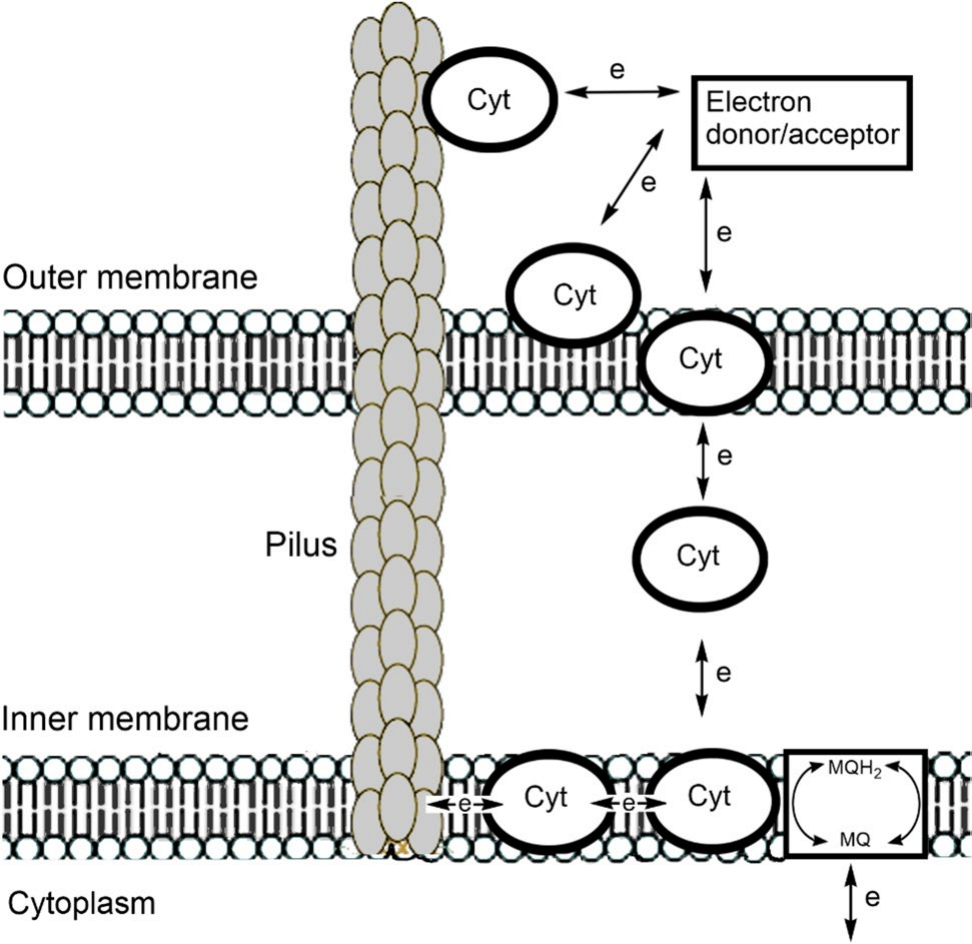
EET in *Geobacter.* MQ, menaquinone. MQH_2_, menaquinol. Ct, multi-haem cytochromes. Adapted from [393]

The electron transfer chain of *Geobacter* is different and complex and uses both electrically conductive pili (e-pili), which are conductive filaments, and cytochromes to effect electron transport [393, 411, 412]. E-pili are long helical structures composed of protein subunits (pilins) which form long, hair-like appendages on the surface of the cell. Their conductivity is due to the presence of aromatic amino acids (especially tryptophan) in the pilin proteins, which facilitate the movement of electrons along the length of the pilus. *Geobacter* contains numerous c-type cytochromes, many of which are multi-heme proteins, distributed across the inner membrane, periplasm, and outer membrane [413]. Current knowledge of the structure and distribution of these cytochromes has been reviewed [393]. A minimal model for the electron transport in *Geobacter sulfurreducens* is shown in Fig. 50.

Other mechanisms for long range electron transfer are known. For example, the *Desulfobulbaceae* family of thermosulfobacteriota [414] are anaerobic bacteria that connect one by one to form a “cable” in surface sediment (so are often referred to as filamentous or cable bacteria) to couple oxidation of H_2_S in the interior, anaerobic, sediment region to the reduction of O_2_ in the oxic region of the sediment [415]. Electron transfer is effected by over 1 cm. Another strategy is to use electron shuttles excreted by the cell (flavocytochromes, phenazines, quinines, flavins) or naturally occurring humic acids to transfer electrons from the cell wall to a distant electron acceptor [416, 417].

## Concluding Remarks

The transport of electrons is fundamental to numerous biological processes, as highlighted by examples in this article. While many others exist, ATP production, cellular respiration, photosynthesis, and redox processes are arguably among the most significant. Moreover, what we learn from biology has immense value in various industrial and commercial applications. A prime example is bioremediation, where microorganisms are harnessed to oxidize or reduce pollutants such as pesticides and petroleum products [267–269, 374, 418–421]. Biological redox reactions also play a key role in the production of biofuels like biodiesel and ethanol [422–425], and biocatalysts are extensively used in the synthesis of pharmaceuticals [426], fine chemicals [427] and agricultural products [428], and other medical, technological and industrial applications [371]. These reactions are integral to biofuel cells, which convert chemical energy directly into electrical energy using enzymes as catalysts [429], microbial electrosynthesis cells [396, 397] and in biosensors [430, 431]. Furthermore, leveraging or mimicking biological transformations for harnessing solar energy will become increasingly important as we—and we must!—transition from fossil fuels to renewable, cleaner energy sources [136–146, 432–434]. While we've gained significant insights from nature, there remain many gaps in our understanding, as noted in several instances. This presents abundant opportunities for groundbreaking research and, crucially, for its broader societal application.

## Data Availability

No original data were used in the article.
